# Engineering mesoporous silica nanoparticles for drug delivery: where are we after two decades?

**DOI:** 10.1039/d1cs00659b

**Published:** 2022-06-01

**Authors:** María Vallet-Regí, Ferdi Schüth, Daniel Lozano, Montserrat Colilla, Miguel Manzano

**Affiliations:** Chemistry in Pharmaceutical Sciences, School of Pharmacy, Universidad Complutense de Madrid, Research Institute Hospital 12 de Octubre (i + 12) Pz/Ramón y Cajal s/n Madrid 28040 Spain vallet@ucm.es; Networking Research Centre on Bioengineering, Biomaterials and Nanomedicine (CIBER-BBN) Madrid 28029 Spain; Department of Heterogeneous Catalysis, Max-Planck-Institut für Kohlenforschung Kaiser-Wilhelm-Platz 1 D-45470 Mülheim an der Ruhr Germany

## Abstract

The present review details a chronological description of the events that took place during the development of mesoporous materials, their different synthetic routes and their use as drug delivery systems. The outstanding textural properties of these materials quickly inspired their translation to the nanoscale dimension leading to mesoporous silica nanoparticles (MSNs). The different aspects of introducing pharmaceutical agents into the pores of these nanocarriers, together with their possible biodistribution and clearance routes, would be described here. The development of smart nanocarriers that are able to release a high local concentration of the therapeutic cargo on-demand after the application of certain stimuli would be reviewed here, together with their ability to deliver the therapeutic cargo to precise locations in the body. The huge progress in the design and development of MSNs for biomedical applications, including the potential treatment of different diseases, during the last 20 years will be collated here, together with the required work that still needs to be done to achieve the clinical translation of these materials. This review was conceived to stand out from past reports since it aims to tell the story of the development of mesoporous materials and their use as drug delivery systems by some of the story makers, who could be considered to be among the pioneers in this area.

## Abstract 2

This review details the huge progress in the design and development of mesoporous silica nanoparticles for biomedical applications during the last two decades.

## Graphical abstract

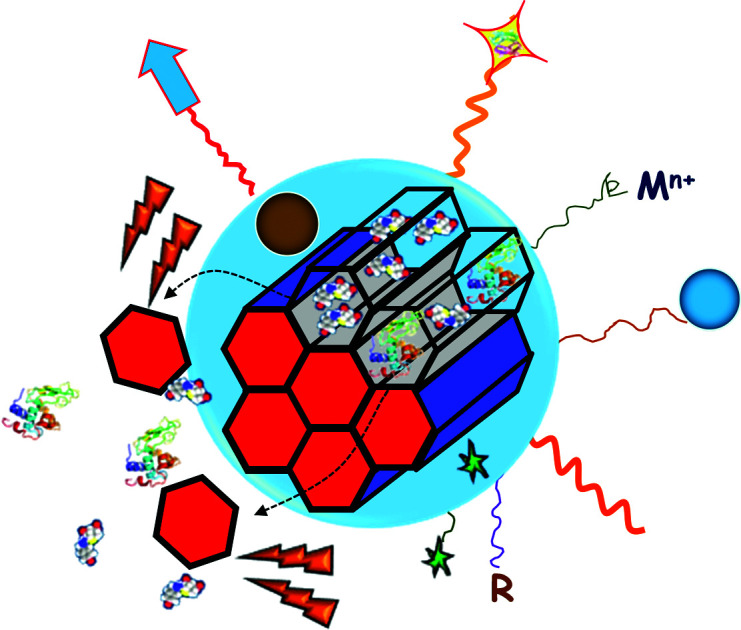

## 1. Introduction

### 1.1. Historical background of the synthesis of ordered mesoporous materials

Carriers for controlled drug delivery had traditionally been based on biopolymers or synthetic polymers,^1^ but in the 1990s, silicas, especially in the form of nanoparticles, moved into the field of interest for scientists working on drug delivery;^2–5^ early silica-based systems were porous glasses or disordered silica gels. However, at about the same time, ordered mesoporous silicas, based on mesostructuring using surfactants, were discovered independently by one of Kazuyuki Kuroda's groups^6^ and by scientists at Mobil Oil Corp.^7^ Since both fields were in their very infancy in the 1990s, it took about 10 years for these separate fields of research to come together. As far as we know, the first potential use of ordered mesoporous materials for drug delivery is mentioned, just as a “buzzword”, in the abstract of a little known paper from 1998 by one of the authors of this review, with no further explanation or discussion of this possible application in the main text of that publication.^8^ The first real description of such silicas as drug delivery matrices was given by Vallet-Regí *et al.*^9^ This seminal paper initiated the broad research field of biomedical applications of ordered mesoporous silicas, a field with very high current activity and more than a thousand publications appearing each year.

Ordered mesoporous silicas have a number of features, which make them highly suitable for drug delivery applications:^10^

An ordered porous network, which is very homogeneous in size and allows fine control of the drug load and release kinetics;

A high pore volume to host the required amount of pharmaceuticals;

A high surface area, which implies high potential for drug adsorption;

A silanol-containing surface that can be functionalized to allow better control over drug loading and release.

**Fig. 1:**
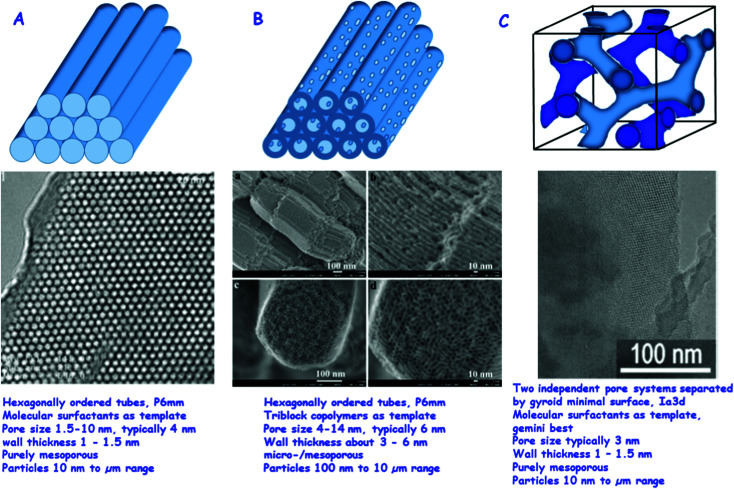
Most relevant ordered mesoporous materials for drug delivery: (A) MCM-41, top: schematic of the hexagonally ordered system of pores, middle: TEM of a high quality MCM-41 sample (reproduced with permission from ref. 11), bottom: typical parameters of MCM-41. (B) SBA-15, top: schematic of the hexagonally ordered system of pores with the micropores in the walls connecting the ordered mesopores, middle: HR SEM images of SBA-15, also here micropores are visible (reproduced with permission from: ref. 12), bottom: typical parameters of SBA-15. (C) MCM-48, top: schematic of the two independent pore systems separated by a gyroid minimal surface, middle: TEM image of MCM-48 (reproduced with permission from: ref. 34), bottom: typical parameters of MCM-48.^11,12,34^ Adapted with permission from ref. 11, 12 and 34. Copyright 2002, 2008 and 2009, Wiley, ACS, Elsevier Ltd.

Fig. 1 gives an overview of the more important ordered mesoporous silicas, which are being used as drug release vectors.^11,12^

Some of these features can also be realized with other materials, such as silica xerogels or aerogels or porous glasses, but all of these properties simultaneously exist only in ordered mesoporous materials. Moreover, to date, various facile methods for the synthesis of ordered mesoporous silica in the nanoparticulate form have been developed, which have several physiological advantages as carriers for bioactive agents. In the following, we will first discuss the early work on ordered mesoporous materials preceding the first publication on drug release and then cover the different pathways for the synthesis of such materials, with a focus on those most relevant for drug delivery. In the final part of the synthesis section, we will address approaches to create ordered mesoporous silica nanoparticles (MSNs), which is the form in which they are best applicable in the biomedicine field.

Surfactants, the key ingredients in the synthesis of ordered mesoporous materials, had been used in silica chemistry before for various purposes, and it is thus not completely surprising that materials resembling ordered mesoporous silicas disclosed in the early 1990s had been synthesized before. Probably the first synthesis of an ordered mesoporous material was described as early as 1971 in a patent,^13^ since an exact repetition of the patent procedure led to the formation of an ordered material having similar features to MCM-41.^14^ However, this initial patented procedure cannot be considered as the discovery of the material, since except for a low bulk density no specific properties are reported in the patent, and therefore the remarkable features of these surfactant-templated materials, such as their regular and ordered pore systems, were not observed. Moreover – and perhaps more importantly from a fundamental point of view – no ideas concerning supramolecular templating as a new synthetic concept were formulated.

The real discovery of the materials and the processes leading to these materials can be dated back to the end of the 1980s/beginning of the 1990s. The first description of a material having linear pores in the size range of 4 nm, which were hexagonally ordered, was published by Kuroda and coworkers.^6^ This paper, however, initially went largely unnoticed, attracting only six citations in the first four years until 1993 (according to the Web of Science, the current citation count is above 1500). This was both due to the journal in which it was published, which is not widely read, and due to the limited scope of the synthetic pathway: the intercalation of sheet silicates and subsequent transformation to ordered mesoporous solids did not seem to be a general route to a large variety of different materials.

This was different from the work published by the Mobil group in 1992;^7,15^ it had previously been patented and also been presented as a recent research report poster at the 9th International Zeolite Conference in Montreal. This poster was probably the most intensively discussed and photographed poster at any conference the authors are aware of, and also some of the authors of this review took photos to immediately replicate the synthesis in their laboratories. In the written form, this work was published in high profile journals, and especially the very comprehensive *J. Am. Chem. Soc.* paper^15^ has a density of new information, which is rarely seen in publications. The extremely high long-term impact of these papers (together around 25 000 citations to date) was probably not due to the description of a material with unusual textural properties; the decisive point appears to be the formulation of possible liquid crystal templating mechanisms, even if they were at that initial stage in a rather general and not very specific form. This introduced a novel concept for the creation of highly ordered, porous inorganic materials beyond the molecular scale, using supramolecular arrays as templates. An account of the history of discovery from the perspective of the Mobil-scientists was given in 2013.^16^

Following this seminal series of papers, important work expanding these discoveries was published. A more detailed formulation of the mechanism was put forward by the group of G. Stucky,^17^ who proposed that under most synthesis conditions the organic/inorganic mesostructure forms cooperatively *via* the interaction between multiply charged silicate oligomers and the positively charged surfactants. Subsequently, in a joint paper by Stucky's group and Schüth's group, the concept was generalized to other conditions, and, maybe more importantly, to other compositions.^18^ A major conceptual development with respect to the mechanism which helps to rationalize many of the synthetic pathways was also introduced by Stucky's group, first only briefly mentioned and not strongly elaborated on in 1995,^19^ but fully developed in a subsequent paper one year later.^20^ It is based on the surfactant packing parameter introduced into surfactant science by Israelachvili,^21^ and allows the prediction of the development of certain phases based on simple geometric arguments.

Another major development line, which started in the middle of the 1990s, was the use of polymeric surfactants. Pinnavaia's group pioneered this approach with poly(ethyleneoxide) based surfactants,^22^ using dilute concentrations, while Attard *et al.*^23^ used similar surfactants, but at such high concentrations that a liquid crystalline phase was present before the addition of an inorganic precursor. This so-called “true liquid crystal templating” (TLCT) introduced a new concept into the synthesis of ordered mesoporous materials. However, the element of predictability, which Attard *et al.* mentioned in their contribution, does not seem to be fully exploitable, since the addition of a precursor to the inorganic material in many cases seems to destroy the liquid crystalline phase, which subsequently reforms as an organic–inorganic composite. The pathway introduced by Pinnavaia and coworkers using more dilute surfactant systems was thus the more influential one.

The use of polymeric surfactants culminated in the discovery of SBA-15 in the Santa Barbara groups of Stucky and Chmelka.^24^ The synthesis of SBA-15 is probably the most important single breakthrough after the original synthesis published in 1992. The properties of the material, such as the tunability of the pore sizes over a wider range than for MCM-41, thicker walls and correspondingly enhanced stability, and the connections between the mesopores through the micropores in the walls, make the material more promising than the original MCM-41 for most applications. This is the reason why over recent years SBA-15 and related materials seem to have been used perhaps even more than MCM-41, judging from the personal impression of the authors and also citation data.

Relying on pre-formed ordered mesoporous materials, Ryoo introduced a novel concept for the negative replication of such systems, first for the formation of a MCM-48 structure as a carbon replica material (CMK-1),^25^ following a related, but not quite as successful, attempt at replicating the pore structure of a zeolite.^26^ This approach has now substantially been broadened, and many different materials are accessible *via* this nanocasting process.

Surface modification of silica is a highly important feature for biomedical applications. In fact, it was back in 1990 when the Japanese Research Group headed by Kuroda reported for the first time the reaction of kanemite-derived mesoporous silica with a trimethylsilylating reagent to form a trimethylsilylated derivative.^27^ Due to calcination of the silylated material at 700 °C, this functionalization was lost, though, in the final porous silica. Later on, a similar functionalization strategy was also applied by the Mobil team to perform the trimethylsilylation of the surface of MCM-41 pores,^15^ resulting in trimethylsilylated porous silica. This method established pathways for the silylation of mesoporous silica. For conventional silicas, also the synthesis of organosilicas from organically bridged silsesquioxanes was well established.^28^ Based on this well-developed chemistry, also ordered mesoporous organosilicas, where each silicon atom in the structure is connected *via* an organic molecule to another silicon center, were created. Such materials are synthesized from organically bridged bis(trialkoxysilane) precursors. The process was independently discovered by three groups: Inagaki *et al.*,^29^ Stein *et al.*^30^ and Ozin *et al.*^31^ Inagaki subsequently also found that organosilicas bridged with phenylene groups had partly ordered wall structures,^32^ a feature which had been searched for essentially from the beginning of the research on ordered mesoporous materials, and only partly been achieved in the synthesis of block copolymer templated SBA-15 type materials, where the walls in some cases consist of nanocrystalline metal oxide domains.^33,34^

### 1.2. Synthetic pathways for the production of ordered mesoporous materials

After the discussion of the major development lines in the early days of this research field, which had established the main classes of ordered mesoporous materials, especially silicas, we will take a closer look at the synthetic pathways available. The discussion will focus on silica, since this is the major matrix for biomedical applications. We will also highlight the options to modify and adapt properties towards specific application fields, such as for drug delivery or other biomedical applications. For the synthesis of specific ordered mesoporous silica, the collection of verified syntheses by Meynen, Cool and Vansant^35^ is a great resource. This compilation does not specifically address the use of ordered mesoporous silicas for biomedical applications, but it is a perfect starting point for obtaining insight into the more often studied structures, their characterization and their synthesis. Moreover, many reviews that focus on the synthesis of ordered mesoporous materials exist; however, we would like to mention only a few focusing on different aspects.^36–39^ Such a deep level of treatment as in these dedicated reviews cannot be given in an article focusing on drug delivery, but the following section serves as an introduction to the more important aspects in the synthesis of ordered mesoporous silica, and provides access to more detailed accounts in the literature.

#### 1.2.1. Creation of the mesostructure

The production of ordered mesoporous materials can conceptually be divided into three different steps: (i) creation of the mesostructure, (ii) creation of porosity, and (iii) functionalization of the mesoporous solid. While the steps cannot always be completely separated, such as, for instance, in the controlled condensation of organosilane precursors to organosilicas, the separation is helpful for the subsequent discussion. However, before the details of the more important synthesis pathways will be discussed, it appears to be helpful to briefly discuss the aqueous chemistry of silicon and the basics of surfactant behaviour in an aqueous solution. The discussion will focus on water as the solvent, since this is the most often used system, although often alcohol is present, from the hydrolysis of silicon alkoxides or because it was deliberately added. In addition, also non-aqueous systems, in which only reagent amounts of water were used to induce hydrolysis and condensation of the silica precursors, have been reported in the literature.^40^ Those non-aqueous syntheses are reviewed elsewhere.^39,41^

#### 1.2.2. Silica and silicates in solution

**Fig. 2:**
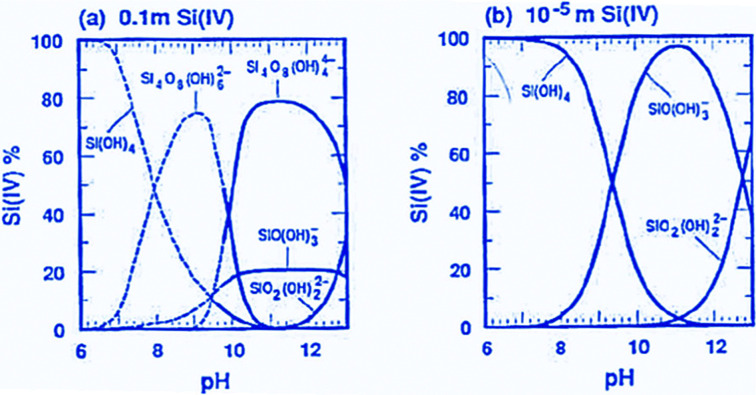
Speciation of silicates in solution at different concentrations over pH (reproduced with permission from Brinker and Scherer, *Sol–Gel Science*, Academic Press, Boston 1990).^43^ Adapted with permission from ref. 43, Copyright 1990, Academic Press.

Silicates condense under a wide range of conditions, including different pH values, temperatures, concentrations, additional electrolytes, and others. The chemistry of silica and silicates has been covered extensively in two excellent monographs: the classical book of Iler,^42^ and a broader treatment by Brinker and Scherer,^43^ which also includes a treatment of the sol–gel chemistry of oxides other than silica. In addition, also a review article by Brinker provides a great overview of the dependencies of the different types of reactions on the reaction conditions.^44^ Silica has a point of zero charge at around pH = 2, which also approximately is the isoelectric point. This means that silica is negatively charged at pH values higher than the point of zero charge, so that silanol groups are deprotonated, and at pH values lower than about two, silica surfaces are partially protonated and thus positively charged. Silicic acid or monomeric/small oligomeric silicates are less acidic, so that a higher fraction of negatively charged small silicon containing units only become predominant around neutral pH values (Fig. 2).

As a first guideline, it is thus useful to distinguish ordered mesoporous silica syntheses proceeding under alkaline conditions, where silicate species are negatively charged in solution and thus have a direct Coulomb-interaction with positively charged surfactants. On the other hand, in the pH range below 7, at least the smaller species only carry little charge, so that more hydrogen bonding interactions between silicic acid species and neutral surfactants predominate in the mesophase formation. Only under very strongly acidic conditions, the species and surfaces are protonated to an appreciable extent, and thus positively charged. For silicas to be applied in drug release or related applications, the point of zero charge around pH = 2 should be kept in mind, if charged bioactive species should be loaded into them. A proper pH range has to be selected, so that electrostatic repulsion is suppressed. On the other hand, too strong electrostatic attraction may lead to difficulties in releasing the adsorbed species. Similar considerations hold for silicate species in solution, but here one has to keep in mind that at low pH, silica gels form rapidly, and solution concentrations of silicon-containing species are rather low.

**Fig. 3:**
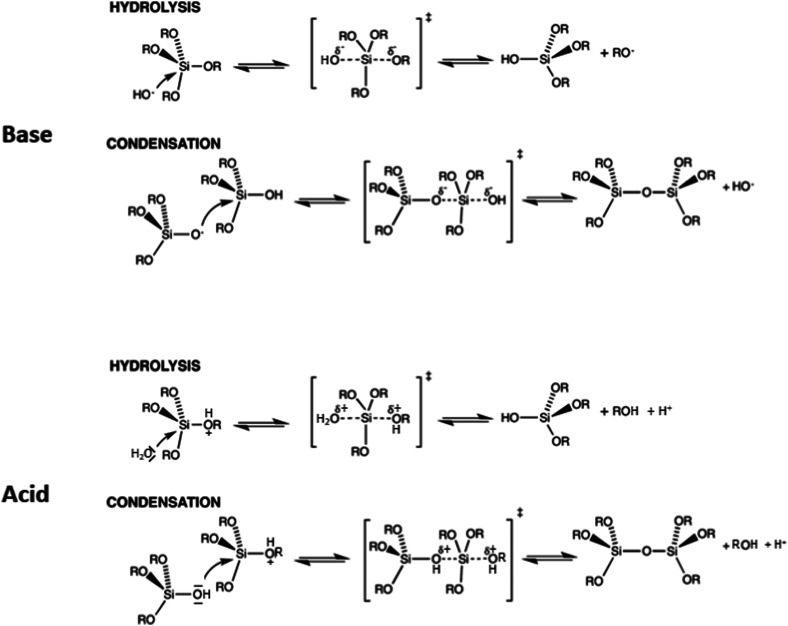
Hydrolysis and condensation reactions of silicon alkoxides and condensation under (top) basic conditions and (bottom) acidic conditions.

As in most solid-state structures of silicates, silicate chemistry in solution is governed by silicon atoms in tetrahedral oxygen coordination. Both hydrolysis and condensation reactions proceed *via* nucleophilic substitution, typically following S_N_2-type reactions *via* a pentacoordinated transition state, although different variants of such reactions are discussed.^44^Fig. 3 gives an overview of the major types of reactions that occur. Under alkaline conditions, OH^−^ typically acts as a nucleophile in the hydrolysis reactions of precursor species, such as alkoxysilanes, with alcoholate species as leaving groups, which are directly protonated after being cleaved off the silicon atom. If highly condensed silica is exposed to alkaline conditions, the nucleophilic attack of an OH^−^ ion can lead to cleavage of silioxane bonds, leading eventually to the dissolution of silica to oligomeric and monomeric species, depending on the pH-value.

**Fig. 4:**
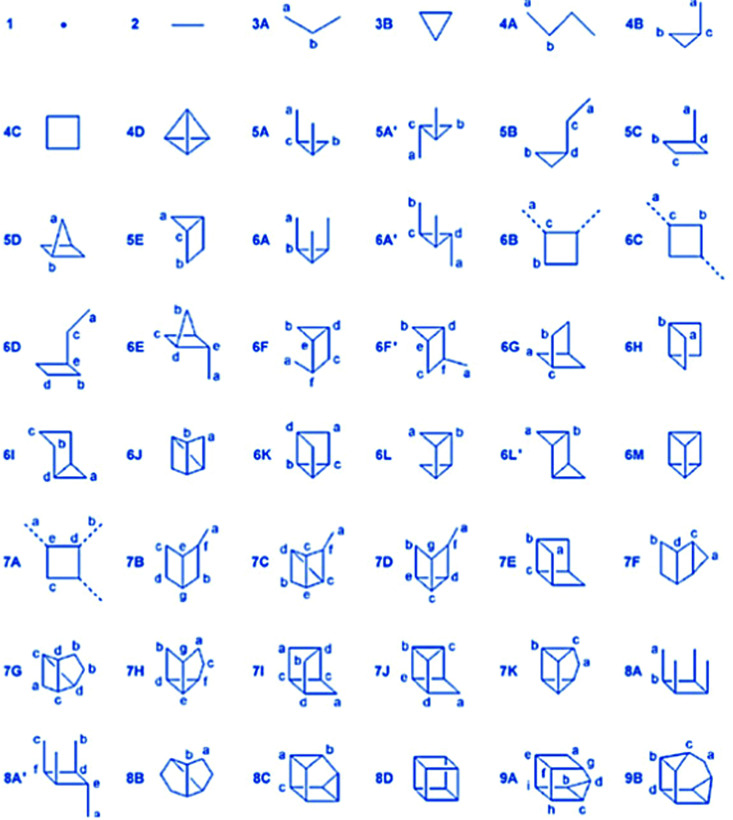
Structure of silicate ions identified in alkaline solution by ^29^Si NMR spectroscopy (reproduced with permission from ref. 48). Adapted with permission from ref. 48, Copyright 2007, Wiley-VCH GmbH.

Silicate species formed by the initial hydrolysis reaction under alkaline conditions are typically deprotonated, and these species are able to initiate a nucleophilic attack on other hydrolysed silica species, leading to the condensation to a siloxane bond. Since more highly condensed silicates are more acidic than monomeric species, they are preferably deprotonated and attack less highly condensed/monomeric species. The systems are dynamic, and depending on alkalinity or other solution conditions, an equilibrium between different silicate monomers and oligomers is established. Fig. 4 summarizes the structures of silicate oligomers, which were detected by ^29^Si NMR spectroscopy under alkaline conditions.

The reactions are different under acidic conditions. Here, potential leaving groups (alkoxy or hydroxyl) are protonated, which makes them more electron withdrawing and easier to be replaced. Under such conditions, the basicity of water is sufficient to allow efficient nucleophilic attack, leading to hydrolysis of the alkoxide group. If the silanol of a monomeric or oligomeric silicic acid is the nucleophile, then a new siloxane bond is formed, leading to condensation to more extended oligomers. A major difference between alkaline and acidic conditions is the fact that siloxane bonds are essentially stable under acidic conditions, *i.e.* oligomers and solid polymeric species are not dissolved, and silica formation is irreversible, while under alkaline conditions, hydrolysis/recondensation reactions prevail, and even for extended silica, exchange reactions proceed to a substantial extent on the surfaces.

For immobilization of drug molecules, the use of more hydrophobic silica could be advantageous. Such silicas are accessible *via* condensation of functional silanes, with pending or bridging organic groups. Organic substituents on the silicon atoms reduce the acidity of silanol groups, and thus they would slow down reactions under alkaline conditions, where silicate anions are the nucleophiles in the condensation reactions, while under strongly acidic conditions, the presence of alkyl groups accelerates the reactions, due to the higher basicity of the oxygen in the silicates. These factors are important in controlling the homogeneity of the alkyl functionalization, since too strong differences in the condensation rates of silicates and partially organically substituted silicates can lead to preferential reaction of one over the other in different stages of condensation reactions, and thus to inhomogeneity in the distribution of the organic groups.

#### 1.2.3. Surfactants in solution

Surfactants are the key ingredients in the synthesis of ordered mesoporous silicas. While initial studies have focused on alkylammonium surfactants, typically with three short chain substituents on the nitrogen atom (mostly trimethyl) and one long chain alkyl group (hexadecyl as the prototypical example), currently, there is a plethora of different surfactants, which have been used for the creation of mesostructured silicas. Ref. 45 gives a great overview not only of the more common types of surfactants that are useful in the synthesis of different ordered mesoporous materials, but also of surfactants useful for other purposes, which could give some inspiration also in the field of mesostructured silica. On a very general level, the charge in the hydrophilic part of the surfactants (positive, negative, zwitterionic, or neutral) governs the type of interaction with the silicon-containing precursors, while the overall geometry of the molecules is important in controlling the type of mesostructure formed. However, eventually the overall conditions of the synthesis are decisive, and the control of the synthesis can hardly be reduced to a single factor.

At low concentrations, surfactants are located at interfaces and in aqueous bulk solution in the monomeric form. At a certain threshold concentration (critical micelle concentration (cmc)), they typically form micelles, mostly spherical in shape. For ionic surfactants, the typical concentration range is 10^−3^–10^−2^ mol L^−1^, while for non-ionic surfactants, micelles already form at about one order of magnitude lower concentrations. At higher concentrations, the spherical micelles may undergo transitions to other shapes, such as elongated or rod-like micelles, and/or they may assemble into two- or three-dimensional ordered structures. A simple and intuitive conceptual framework for the prediction of the kind of structures, which form, is provided by the surfactant packing parameter concept developed by Israelachvili.^21^ Structures of micelles are predicted based on geometric constraints, and certain threshold-values for the parameter *g* = *V*/(*a*_0_ × *l*) indicate the transition points between the structures, where *V* is the volume of the surfactant hydrophobic chain, *l* is the length of the extended hydrophobic chain, and *a*_0_ is the surfactant headgroup area per molecule at the surface of the hydrophobic core. With increasing packing parameter, the expected sequence of structures is shown in Table 1.^20^

**Table 1:** Description of micelle types depending on the packing parameter

Micelle type	Mesostructure	Packing parameter
Spherical	Cubic (*Pm*3*n*)	1/3
Rod-like	Hexagonal (*P*6*m*)	1/2
Complex	Cubic (*Ia*3*d*)	1/2–2/3
Lamellar	Layered	1

These values give indications as to which molecular parameters of the surfactant should be changed if the synthesis should be directed from one structure to another under otherwise identical conditions (if this is possible). While this concept has proven to be very powerful in rationalizing and predicting the formation of certain mesostructures, one should keep in mind that it has originally been developed for equilibrium systems, and even under equilibrium conditions, other solution parameters can strongly influence the formation of specific micelles.^46,47^ A more important point, however, is the fact that the formation of ordered mesostructured materials is normally a kinetic phenomenon, in which the structure forms cooperatively between silicates and surfactants. So, trends can often be well predicted, while precise numerical values for transitions between structures are more difficult to derive.

##### Alkaline pathways

Both the initial pathways for ordered mesoporous materials, the one described by Yanagisawa *et al.*^6^ and the one introduced by the Mobil team,^7^ proceed under alkaline conditions, albeit at rather different levels of alkalinity. While the latter uses fairly high concentrations of OH^−^ (approximately 0.1 M, the OH is introduced with the trimethylammonium surfactant), the conversion of Kanemite takes place at a pH in the range of 8–9. While this difference on first sight does not appear to be very substantial, with respect to silicate chemistry these conditions lead to totally different speciation: at high pH, silicates are typically present in monomeric or small oligomeric form, with a high number of different species structurally identified by ^29^Si NMR spectroscopy.^48^ Under relatively high alkalinity, these species convert rapidly between each other, and there is a condensation/hydrolysis equilibrium, which is dependent on various parameters, such as pH, concentration of silicate, additional ions and species present in solution, and temperature.^42,43^ In contrast, at a pH in the range of 8–9, siloxane bonds are relatively stable, and depolymerisation of condensed silica phases is very slow. This particular feature of silicate chemistry led to the formulation of the model of layer buckling for the conversion of intercalated kanemite to ordered hexagonal structures, as opposed to a dissolution/reprecipitation mechanism, which might prevail at a higher pH.^49^ Due to the sensitivity of silicate chemistry – and thus the synthesis of ordered mesoporous silica – on pH, pH control during formation of the mesostructure can be highly important to influence the quality of the final product. This has first been introduced by Ryoo and colleagues,^50^ who adjusted the pH to 11 for optimum mesostructuring under the conditions used, and subsequently in various publications. Overall, the pH value for synthesis under alkaline conditions mostly seems to be in the range between approximately pH = 9 and pH = 12.

There is one class of ordered mesoporous silicas, which are synthesized at relatively low alkalinity, almost close to neutral pH, which are the anionic surfactant templated mesoporous silicas (AMS) introduced by Che *et al.*^51^ and reviewed by her in 2013^52^ and described in more detail in a recent book.^53^ Up to the initial publication on AMS, synthesis attempts using anionic surfactants had not been very successful. Under acidic conditions, where at least weakly positively charged silicon-containing species exist in solution, the anionic surfactants are typically protonated and thus neutral, resulting only in weak interactions. Under basic conditions, the anionic surfactants are indeed anionic, but also the silicate species carry negative charges. Any charge interaction thus needs to be mediated by cations, also resulting in interactions too weak for the formation of a well-developed mesostructure. A successful solution to this problem was the use of a dual-functional co-structure directing agent (CSDA). This on the one hand can link to the silicate precursor, and on the other hand provides a charge interaction with the anionic surfactant. A prototypical example for such species is 3-aminopropyltrimethoxylsilane, which reacts in a “neutralization” reaction with an acidic precursor of an anionic surfactant on the one hand and co-condenses with tetraethoxysilane with the silane moiety of the CSDA. Alternatively, the positive charge interacting with the anionic surfactant can directly be introduced into the CSDA, for instance in *N*-trimethoxysilylpropyl-*N*,*N*,*N*-trimethylammonium chloride; the trimethoxysilyl-part again co-condenses with the TEOS. A broad range of different materials with various structures can be formed *via* this pathway, and the materials include those with mesoscopic chirality.^52^ AMS type materials have also been studied with respect to their drug release properties, with the advantage that some anionic surfactants are physiologically less critical than most other surfactants and may thus be left in the material to control release.^54^

As opposed to acidic and near neutral conditions, more strongly alkaline conditions allow a greater variability in the nature of the silica precursor. Under acidic conditions, solid silica or silica sols essentially do not dissolve and are thus not suitable as precursors; hence, typically alkoxysilanes are used as the silica source. Under alkaline conditions, such silanes are suitable precursor species as well, but also basic silicate solutions with different cations (alkali ions, tetraalkylammonium), fumed or colloidal silicas as well as silica gels can be converted to ordered mesoporous materials. In fact, in the original synthesis reported by the Mobil team, a mixture of tetramethylammonium silicate and precipitated silica was employed,^7^ but already in the broader follow-up publication, a wider range of silica precursors was studied. The nature of the silica precursor certainly does have an influence on the synthesis and needs to be taken into account, but under suitable conditions, it seems that almost any silica precursor can be converted to ordered mesoporous silica. Care has to be taken that highly condensed silica is depolymerized at least to some extent before the mesophase formation, and complex precursors should at least be hydrolysed to some extent, but then addition of the surfactant and adjustment of pH will induce mesophase formation under the right conditions, which is only little dependent on the silica source.

The key element to structure the mesophase is the surfactant, but its influence is moderated by the synthesis conditions, *i.e.*, temperature and ratios of the concentrations of silicate, surfactant and alkalinity. In the Mobil publications, two structures were introduced, MCM-41 and MCM-48, in addition to the layered MCM-50, which is unstable upon calcination and will not be further considered here. Both were synthesized using cetyltrimethylammonium bromide (CTAB), but cubic *Ia*3*d* MCM-48 formed at CTAB/Si ratios exceeding 1 under precisely defined conditions, while the hexagonal tubular MCM-41 formed at lower CTAB/Si ratios over a broader range of conditions. This, however, is also dependent on the surfactant chain length and other synthesis parameters.^55^ MCM-48 is substantially more difficult to synthesize than its hexagonal MCM-41 counterpart. It seems that the best approach for its controlled synthesis is the use of gemini surfactants instead of the CTAB and related surfactants, since this allows better control over the surfactant packing factor^18,56^ in controlling drug release and thus important to be controlled for this and related applications.^57^ Over a relatively small range, the pore size of the resulting silica can be adjusted by the length of the hydrophobic chain of the surfactant, with a longer surfactant tail leading to bigger pores. However, above a certain threshold around C_20_/C_22_ high quality hexagonal mesophases do not form any more, and there are only scattered reports in the literature in using such long-chain surfactants, which demonstrate that precise control of conditions is required to obtain the desired mesostructure.^58^ Further expansion of pore sizes has to be achieved by adding auxiliary organics, such as trimethylbenzene, which is incorporated in the hydrophobic part of the micelles and thus leads to swelling. This allows expansion of pore sizes for the hexagonal material to about 10 nm, although control becomes increasingly difficult towards the higher end of the pore size. By judicious choice of conditions, additives, and hydrothermal conditions, pore sizes can be controlled fairly precisely for MCM-41-type materials between approximately 1.5 nm and close to 10 nm.^59^

For biomedical applications, one highly important factor is the control of size and shape of the ordered mesoporous silica, since this governs on the one hand the transport of silica in the organism, and on the other hand it is important – among other factors – for the kinetics of, for instance, drug release. However, since this is a crucial factor in the context of this review, it will not be discussed here, but a separate section below will be devoted to this aspect.

##### Acidic pathways

Acidic synthesis pathways were introduced in the publication of Huo *et al.* in 1994.^18^ Generally, under acidic conditions the synthesis temperature tends to be somewhat lower than that of alkaline systems; often the synthesis is carried out at room temperature or even below, while under alkaline conditions, the temperature is often above room temperature up to about 120 °C. In the publication by Huo *et al.*, in addition to the S^+^I^−^ and S^−^I^+^ pathways (S surfactant, I inorganic species) for the formation of mesostructured materials, two mediated pathways (S^+^X^−^I^+^ and S^−^M^+^I^−^) were introduced, in which anions, such as halogenides, or metal cations mediate the Coulomb-interaction between surfactant and condensable species. For the synthesis of silica *via* the S^+^X^−^I^+^ pathway, the reaction was carried out at very low pH (1–7 M HCl or HBr) so that silicon containing species are positively charged. In addition to the hexagonal phase and the lamellar phase also obtained under basic conditions, a cubic *Pm*3̄*n* mesophase was formed (in a later study labelled SBA-1)^20^ with surfactants with a larger headgroup (alkyltriethylammonium, cetylethylpiperidinium) to adjust the packing parameter towards high curvature structures. The *Pm*3̄*n* phase was known also for the pure surfactant systems in formamide, but it could be created as a surfactant–silica composite under acidic conditions.^60^ Support for the mediated pathway comes from the facts that the chlorine to surfactant ratio is one, and the surfactant can easily be removed by washing in ethanol. In addition – as generally valid for silica formation under acidic conditions – the synthesis proceeds with TEOS or SiCl_4_, but not with condensed silica, such as Cab–O–Sil, because this does not depolymerize under acidic conditions, so that insufficient silicon containing oligomers are in the solution to form the mesophase. With tailored surfactants, under highly acidic conditions also a hexagonal phase termed SBA-2 is obtained, which can be described as a hexagonally close packed array of surfactant silicate micelles with the *P*6_3_/*mmc* space group.^20^ This is one of the surfactant–silica mesophases which do not have a corresponding pure surfactant analogue – at least as yet, although recently indications of the presence of a *P*6_3_/*mmc* phase were reported in a glycerol monooleate/oleic acid system.^61^ Overall, however, SBA-1 and SBA-2 have not been explored as extensively as many other ordered mesoporous silica, although the structures are interesting and they can be obtained in the form of well-developed single crystals of different morphology.^62^ A recent, comprehensive survey on ordered mesoporous silica synthesized under acidic conditions *via* the S^+^X^−^I^+^ pathway has been given by Jarmolinska *et al.*^63^

A very important – and by now possibly most often used – class of ordered mesoporous silicas is synthesized using non-ionic surfactants. At moderate acidity or near neutrality, silicon-containing oligomers carry almost no charge, and thus hydrogen-bonding interactions with the surfactants are playing the major role in structure direction. The use of non-ionic surfactants under such conditions (S^0^I^0^) was pioneered by Pinnavaia's group, who first used long-chain amine surfactants^64^ to synthesize materials resembling MCM-41, but with thicker pore walls. Template molecules could be extracted, because they are more weakly bonded to the silicate. While this synthesis proceeds at pH slightly above neutral due to the weakly basic properties of the alkylamine, the process using polyethyleneoxide-based neutral surfactants, also introduced by Pinnavaia's group only a few months later, proceeds at very mild acidity at around pH = 6.^22^ Here, hydrogen bonding interactions are the dominating force, and interestingly, the hydrolysis of the TEOS precursor proceeds rapidly within a few minutes in the presence of the surfactant, while it takes hours without it. The products, labelled MSU-1, are less ordered than the other ordered mesoporous silicas described above and rather have wormhole structures, but pore size distribution is almost as sharp and well defined as in MCM-41.

Possibly the most versatile synthetic routes make use of block copolymer templates, with different types of blocks, and most of these syntheses are carried out again under strongly acidic conditions. Under these conditions, it is assumed that a combination of Coulomb interactions, hydrogen bonding interactions, and van der Waals interactions is responsible for the formation of the mesophase. In the shorthand notion this is labelled as (S^0^H^+^)(X^−^I^+^), *i.e.* the alkylene oxide moieties are partially protonated under strongly acidic conditions, the positively charged silicon-containing units have halogenide or other anions coordinated to them, and the superposition of different interactions controls the formation of the mesostructure.^65^ The details of the contributions of each of them is certainly governed by the exact synthesis conditions.

Most often used are probably the block copolymers of the Pluronic™ type, (IUPAC name: poly(ethylene glycol)-*block*-poly(propylene glycol)-*block*-poly(ethylene glycol), often, however, labelled as poly(ethyleneoxide)poly(propyleneoxide)poly(ethyleneoxide), and abbreviated as PEO–PPO–PEO. These types of surfactants were introduced in the field of ordered mesoporous silica by Zhao *et al*.^24,65^ The compositions, which have been created using block copolymer surfactants, are excellently reviewed in ref. 39, but the most often used one is certainly Pluronic P123 (EO_20_PO_70_EO_20_; note that compositions are only approximate). The use of this surfactant results very reliably in the formation of the hexagonally ordered SBA-15 over a wide range of synthesis conditions at pH-values below 2. Also KIT-6, the block copolymer-templated analogue of *Ia*3*d* MCM-48 and another highly popular material for many applications, can be synthesized using P123 by modifying the synthesis conditions, especially by adding *n*-butanol.^66^ By adjusting the block length in these block copolymers, other structures are synthesized. Pluronics F127 (EO_101_PO_56_EO_101_), for instance, leads to the formation of SBA-16, a cubic cage structure with the space group *Im*3̄*m*. The comprehensive publication by the Santa Barbara team^65^ describes a wide range of different polymer surfactant types, with several members tested for each of these. In particular, for applications in drug release, control of the channel length can be important, and the verified synthesis publication by Meynen *et al.*^35^ gives guidelines how to produce especially SBA-15 with short channels.

At first sight, MCM-41 and SBA-15 (and analogous other structure pairs, such as MCM-48 and KIT-6) appear to be rather similar, with the exception of the wall thickness. However, there is an additional difference, which is less obvious, and this is the presence of micropores in the walls of the block copolymer templated materials, connecting the mesopores, which was first inferred from nitrogen sorption analysis.^67^ Later these micropores were directly imaged as bridges between the mesopores after replication of the pore structures using platinum.^68^ These micropores are formed, because the poly(ethyleneoxide) moieties of the block copolymer surfactants as the more hydrophilic part are embedded in the pore walls, and upon calcination are removed to result in micropores connecting the mesopores.^69^ These micropores in the walls of block copolymer templated silicas are important with respect to the replication of the pore system, but also for potential application as matrix for drug release, since the adsorption in these micropores can be stronger than that in the mesopores, provided that they are sufficiently big for adsorption of the payload.

**Fig. 5:**
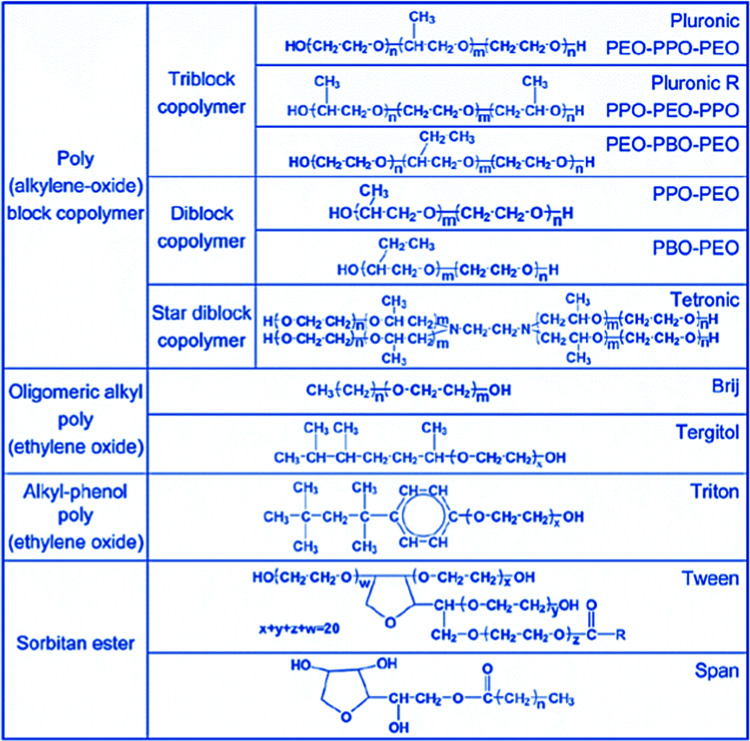
More important polymeric surfactants used for the synthesis of ordered mesoporous materials. Reproduced with permission from ref. 39, Copyright 2007, The Royal Society of Chemistry.

In addition to block copolymers, alkyl poly(ethyleneoxide) surfactants were also found to be highly versatile in this and many other studies. Fig. 5, taken from ref. 39, gives an overview of the type of non-ionic surfactants used in the synthesis of ordered mesoporous silica. By now, syntheses under acidic conditions using polymeric and block polymeric surfactants seem to have superseded the original alkaline synthesis routes, at least judging from citation numbers (WoS for the last five years: SBA-15: 4357 citations, MCM-41: 3143 citations, KIT-6: 496 citations, and MCM-48: 263 citations).

#### 1.2.4. Nanocasting

The nanocasting pathway is mentioned here only for completeness. In nanocasting, initially formed ordered mesoporous materials synthesized by solution methods are used as hard templates for the replication of the pore system by another solid phase. This has first been demonstrated in the synthesis of CMK-1 by Ryoo's group,^70^ in which the pore structure of MCM-48 was replicated as a carbon material after infiltration with a suitable carbon precursor. There are several very useful reviews and books available, in which nanocasting is treated in detail.^71–77^ However, for applications in drug delivery, nanocasting is of limited use, since it is mostly used for the synthesis of non-siliceous materials, with mesoporous silica as a hard template. Non-silica materials are not well suited for drug delivery, since most compositions are physiologically less favourable than those with silicas and have thus only little been used for such purposes. Nanocasting can indeed be used for the synthesis of ordered mesoporous silica, for instance by repeated nanocasting, where first a carbon replica is obtained from a silica, and the carbon in turn is replicated as silica again.^78^ However, although the repeated nanocasting does result in replicas with somewhat altered properties compared to the original template, access to such silica by direct synthesis is mostly much more straightforward and easier. Thus, while nanocasting is highly valuable for the synthesis of non-silica mesostructures, its relevance for drug delivery applications is very low.

#### 1.2.5. Template removal

For drug delivery applications, the drug needs to be loaded into the carrier, in the case discussed here into ordered mesostructured silica, and for this, the template typically has to be removed from the mesostructure first. In principle, it is possible to incorporate the payload directly during synthesis, if the drug molecules are not degraded during the synthesis of the mesostructure – which is more probable for syntheses close to neutral pH. However, even then regulatory issues with potential degradation products may arise, and thus the *in situ* incorporation does not seem to be a practical method for drug delivery applications. Moreover, if the surfactant template is not removed, one has to consider the physiological effects of the surfactant as well. There are isolated reports that for innocuous surfactants they can remain in the material also for drug release applications,^54^ but such approaches do not seem to be widespread.

Thus, in order for mesoporous silica to be useful as a carrier for drugs, the templates have to be removed from the pore system, before the drug is loaded into the carrier. Conventionally, this is done by calcination, typically at temperatures exceeding 500 °C, at which essentially all organic matter present in the material is combusted. Final temperatures are normally reached by controlled heating ramps, typically 1 K min^−1^. Also potential residues from different anions are removed, for instance, chloride as HCl.^79^ Depending on the exact nature of the mesostructured material and the nature of the template present, different processes, like Hoffmann degradation of ammonium or amine species, oxidation, carbonization, or oxidation of carbonaceous species, may occur and may be superimposed in different temperature regimes.^79^ While calcination leads to the removal of the surfactant template, also the silica is changing. Typically, the degree of condensation increases, as visible in a change in the Q^3^/Q^4^ ratio detected by ^29^Si NMR spectroscopy, which often leads to substantial shrinkage of the unit cell parameter. Also, the population of silanol groups on the surface is a function of the calcination temperature. Since this is an important parameter for the possibility to adjust the surface chemistry, for instance by alklysilylation, one should keep this factor in mind, since often calcination is uncritically used following standard protocols.

In order to allow – at least thermally – more gentle calcination, some additional protocols have been developed, already early highlighted by Patarin.^80^ Better control is reported for template removal by glow discharge,^81^ non-thermal plasma treatment,^82^ or dielectric barrier discharge plasma,^83^ which results in higher silanol surface population and lower shrinkage of the mesostructure. Surprisingly, also microwave treatment seems to work for template removal. While first only a combination of solvent extraction and microwave treatment was shown to work,^84^ later microwave treatment alone was found to be successful in removing the P123 template. About 95% of the template was reported to be removed by treatment for 40 min at 700 W power and 2.45 GHz microwave frequency.^85^ The resulting detemplated material had substantially increased pore volume and surface area.

Another method for milder template removal is the use of ozone instead of air in the calcination process.^86–88^ Conventional thermal treatment usually results in a highly exothermic uncontrollable reaction, but treatment with  ozone was possible, leading to larger pores than for conventional calcination. Additionally, a higher silanol density was retained.

Recently, an ozone treatment protocol was described, which avoids the problem of the vigorous reaction, so that also dry, powdered ordered mesoporous materials could be calcined under gentle conditions.^88^ Both cetyltrimethylammonium and Pluronic P123 could be removed by ozone treatment at temperatures around 80 °C to reach carbon levels in the lower single digit percent range.

An alternative to thermal removal of the templates from ordered mesoporous silica is extraction or reactive extraction, *i.e.* destruction of the template species in the pores, followed by extractive removal from the pores. Moreover, also partial destruction of the templates in the pores by treatment with a specific reagent, followed by a thermal treatment step under more gentle conditions than those of a single calcination step, is an option.^89^ This method was used to remove the P123 surfactant first from the mesopores of SBA-15, leaving the micropores in the walls blocked, then removing the template from the micropores by milder calcination. Such a process makes selective functionalization of the walls of the mesopores and the walls of the micropores possible.

Extractive template removal had already been attempted early on in the field of mesoporous silica. For MCM-41 and related systems, this is difficult, since there is a Coulomb interaction between the positively charged surfactant and the silicate wall. Thus, a solvent alone is normally insufficient, and an additional charge compensation has to be supplied, for instance in the form of protons. This was clearly demonstrated by a removal level of only about 20% of the surfactant after stirring at 78 °C in ethanol or isopropanol, while around 70% were extracted, when the solvent contained additional ions, such as protons, ammonium, or sodium.^90^ Also in follow-up studies it became clear that template removal was not complete in an extraction process and small amounts of template still remained in the sample.^91^ The latter study also included attempts at reactive extraction, and H_2_O_2_ was used for degradation/extraction of the template. The use of H_2_O_2_ had been introduced earlier for template removal from SBA-15, also in combination with UV irradiation.^92^ Neither UV nor H_2_O_2_ treatment alone was sufficient, but the combination was claimed to remove all template species from the material.

Overall, it seems easier to remove templates extractively, if the original ordered mesoporous silica had been prepared under acidic conditions either *via* the S^+^X^−^I^+^ pathway or with neutral templates, such as long chain amines or polyethyleneoxide based ones. This had already been described in the early publications on these materials^18,22^ Extraction of the Pluronic block copolymer surfactant, on the other hand, is more difficult, because the ethyleneoxide blocks are embedded in the silica walls and thus relatively tightly anchored there. Nevertheless, substantial removal of the P123 template was reported after extraction with ethanol-water 1 : 1 (in the same paper, for MCM-41 an ethanol/aqueous HCl mixture had been used).^93^

Reactive (*i.e.* oxidative) extraction is able to reduce the template content of different ordered mesoporous silica to very low levels, while retaining good textural properties and a high concentration of silanol groups. Tian *et al.*^94^ used microwave heating of SBA-15, SBA-16, FDU-1, or MCM-41 suspended in highly concentrated HNO_3_/H_2_O_2_ to degrade the surfactant to carbon contents below the detection limit of 0.3%, while essentially retaining the silanol groups of the as-synthesized material. A related version, but using reflux at 80 °C instead of microwave heating, was used to produce high-silanol group containing mesoporous silica to be used as hard templates.^95^

A direct comparison of different template removal methods (calcination, extraction with ethanol/HCl, and H_2_O_2_ treatment) from SBA-15 was recently provided by Barczak.^96^ It can be clearly observed that the properties of the resulting material with respect to porosity, surface area, and silanol content were rather different, with the calcination method resulting in a strong loss of silanol groups, while the two other methods resulted in high silanol concentrations, with the oxidative treatment even inducing a substantial increase as compared to the as-synthesized material.

Overall, if a silanol-rich pore surface is desired, either for more hydrophilic behaviour or subsequent functionalization, extraction or reactive extraction seem to be the better options for template removal. In addition, extractive methods are clearly preferable, if template should be removed from organically functionalized silicas obtained by co-condensation with organosilanes. While it may be possible to leave the incorporated or pending organic groups in or on the silica basically untouched by carefully adjusted calcination protocols,^97^ or a combination of short extraction and mild calcination, extraction is a more generally applicable method.

#### 1.2.6. Surface modification

The surface properties of ordered mesoporous silica can be modified by the incorporation of inorganic species, or by grafting or incorporating various organic groups. However, while silica is physiologically not problematic, this does not hold for many metal ions which may be used to modify silica, and thus inorganic surface modification will not be discussed here; further discussion will focus on organic groups.

In the previous section, the different concentrations of silanol groups after using different ways to remove the surfactant template from the pores of ordered mesoporous silicas have already been discussed. This is of high relevance for the use of ordered mesoporous silicas as a drug delivery agent, since surface functionality is decisive for the interaction of potential drug molecules with the surface, and thus loadability and release kinetics of the drug. Surface properties already differ as a consequence of different silanol densities, but beyond the difference in silanol density surface properties can be adjusted by grafting of specific groups or co-condensation with functional silanes.^98,99^

There are different types of silanol groups on the surface of silica, *i.e.* isolated SiOH groups, geminal groups (two OH-groups on one silicon atom), and vicinal groups, (two OH-groups close enough to form a hydrogen bridge between them, normally on neighboring silicon atoms). The population of these silanols is temperature dependent.^100^ Hydroxylated amorphous silica has – basically independent of the source of the silica and the surface area – a silanol density of around 5 nm^−2^ of these groups, as has been shown in an extensive study by Zhuravlev, in which a high number of different silicas resulting from different syntheses and with widely differing surface areas had been analyzed.^101,102^ Incidentally, this number approximately agrees with the density of silicon atoms on the octahedral face of β-cristobalite,^102^ and thus appears to be in the right range. There are reports in the literature claiming higher concentrations up to 8 nm^−2^, and the deviations were at least partly explained by a possibly overestimated surface area in the work of Zhuravlev.^103^ Also other explanations for discrepancies of reported data are discussed in the literature.^93^ There is agreement in any case that thermal treatment – such as calcination – leads to a reduction of the silanol density, with the density being reduced to about half of the original value by vacuum treatment at 400 °C,^102^ and it is thus no surprise that ordered mesoporous silicas detemplated by different types of low temperature processes have higher silanol densities and are thus more hydrophilic than calcined samples.

There are indications that calcined MCM-41 shows heterogeneity of the silanol surface coverage.^104^ However, after extraction or calcination/re-hydroxylation by various methods, the surface properties seem to approach the situation of regular amorphous silica, with a silanol density of around 4.5 nm^−2^ for MCM-41.^93^ Silanol concentrations are reported to be somewhat higher for SBA-15 than for MCM-41, but this could be related to more pronounced degradation during re-hydroxylation of SBA-15, or to problems in determining precise values for surface areas in the micro-mesoporous SBA-15 compared to the purely mesoporous MCM-41. Ibuprofen sorption capacity also correlates with silanol density and thus hydrophilicity of MCM-41. Materials with densities between 4.2 SiOH nm^−2^ and 5 SiOH nm^−2^ were studied, and for the more hydroxylated material, the ibuprofen loading increased by about a factor of 1.5.^105^ Nevertheless, although general statements with respect to silanol density on the surface of ordered mesoporous silica are possible, as discussed above, exact values are dependent on the synthesis protocol and history of the materials, so that analysis of the used sample is required to obtain information on specific materials. If this is not possible or does not seem to be required, the value for hydroxylated silica of about 5 nm^−2^ seems to be a good approximation for most materials, based on the available data in the literature.

##### Surface modification by grafting

The silanols on the surface of silica are the reactive sites, which can be used for functionalization. The different types of silanol groups have different reactivity, with the isolated silanols normally being the most reactive ones. Another important factor is the accessibility of the groups, which is also a function of the size of the species to be grafted onto them.

**Fig. 6:**
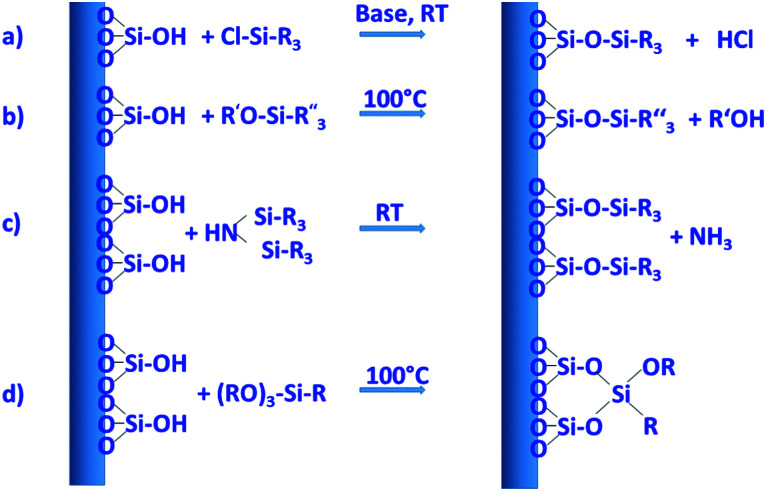
Important reactions with functional silanes for surface modification. Reactions mostly proceed in a solvent, water is typically excluded, because otherwise the silanization agents are hydrolyzed in an uncontrolled manner away from the surface. (a) Functional monochlorosilane, (b) functional monoalkoxysilane, (c) functional disilazane, silicon atoms in the disilazane can also be bridged *via* R-groups, and (d) functional trialkoxysilane, here attachment *via* one, two, or three siloxane bonds to the surface is possible, moreover, functional silanes can crosslink with themselves and thus produce oligomeric/polymeric surface-bound species. The example shown has one alkoxy group left for further crosslinking. As in general silicate chemistry, silicate species avoid edge or face sharing tetrahedra linked to each other.

By far the most often used method for introducing different types of functional groups to the surface is the reaction with functional silanes. Functional silanes contain hydrolyzable groups, which react with the silanol groups of the surface to create a siloxane bond. Suitable precursors are chlorosilanes, alkoxysilanes, or disilazanes (Fig. 6), of which many derivates are commercially available. If controlled surface functionalization is desired, water needs to be excluded, so that the silanes only react with the surface silanol groups and not with water, which would lead to uncontrolled hydrolysis and potential (partial) self-condensation/oligomerization/polymerization of the silanes.^100^ Typically, silylation proceeds in organic solvents, but gas-phase silylation is an alternative, which works for reasonably volatile silanes.^100^ A very simple, fast, and convenient method, which was recently described, uses mechanochemical silylation.^106^ SBA-15 silica was mixed with silane and loaded into the jars of a shaker mill. After relatively mild milling for typically less than 10 min, surface functionalization was complete, for alkoxy- and chlorosilanes bearing different organic groups. No solvent or base are required for this functionalization route; hence it appears to be a convenient alternative.

Silylation with silanes bearing only one hydrolyzable group, with chlorotrimethylsilane as the prototype example, allows cleanest functionalization, since there is essentially no crosslinking between the grafted groups, and the surface functional groups reach a high density, because each silicon atom carries three such groups. On the other hand, only one siloxane bond needs to be broken to remove the grafted species, so that under conditions, where siloxane bonds are attacked, the functionalization may be lost, at least partly. Three hydrolyzable groups on the silane, such as, for instance, in trimethoxypropylsilane, may form a less defined organosiloxane polymer bound to the surface with different motifs; however, since the siloxane network is more extended and crosslinked, the functionalization can be more stable.

Several comparative studies have shown that silylation with hexamethyldisilazane is the most effective silanization method, reaching high degrees of functionalization under gentler silylation conditions. In each case, however, the reaction is strongly dependent on the chemical nature and steric requirements of the functional groups on the silane.^107,108^ The basic type of functionalization is just used to control the hydrophilic/hydrophobic surface properties by introducing nonpolar alkyl groups, in the simplest case methyl groups, but also longer alkyl chains can be used. Effectiveness of the silanization reaction is improved, if nucleophiles are present to promote the hydrolysis/condensation reactions.^107^ If silanes with functional groups beyond alkyl groups are grafted to the silica, the additional nucleophile might not be necessary, if the functional group itself provides the nucleophiles. This is the case, for instance, for aminopropyl-groups, which were often used to introduce basic functionality or to allow further modification.

Using these and related techniques, essentially any functional group can be anchored to the surface of silica. For modified silica to be used as drug release agents, especially introduction of acidic or basic surface groups, beyond adjustment of hydrophobicity, appears to be interesting, because this allows controlled interaction to adjust uptake and release properties. To create basic sites, the aminopropyl-group mentioned above is probably the most often used type of functional group, with 3-aminopropyltrimethoxysilane, due to its ready commercial availability and ease of use, being the most popular silane. Acidic sites are somewhat more difficult to generate. Silanol groups react directly with chlorosulfonic acid with the elimination of HCl to produce directly anchored sulfonic acid groups.^109^ However, a two-step pathway, first involving anchoring of a thiol group with a functional silane to the silanol group, followed by oxidation of the thiol to sulfonic acid, seems to be more often used to introduce acidic groups.^109^

The field of silica surface modification has been very well developed over more than 50 years, not only for the functionalization of ordered mesoporous silica, but for many types of silica, for instance to tailor materials for chromatography. More detailed information can be found in various, partly quite extensive, reviews.^98,100,109,110^

##### Co-condensation with functional silanes

The creation of organosilicas, *i.e.* silicas, in which silicon-carbon bonds exist, is also possible using one-step procedures, if silanes with corresponding moieties are used as silica precursors. Such functional silanes can be used for the synthesis of mesostructured silica without additional surfactants, if a condensable silane, for instance with trialkoxy-groups, contains also one long chain hydrocarbon, such as octadecyltrimethoxysilane. If such precursors are co-condensed with a tetraalkoxysilane, the silane with the long hydrocarbon chain acts as a porogen.^111^ However, for drug release applications, this is of little use, since the pore system is then occupied by the alkyl chains, which have to be removed by calcination, resulting in an essentially pure silica material without organic functionalization. More interesting for drug release applications are co-condensation synthesis protocols, where a functional silane with shorter organic groups is reacted with, for instance, TEOS in the presence of separate surfactant molecules, an approach, which had already been introduced in 1996,^112,113^ and many different functional groups can be incorporated *via* this pathway.^114^ For the prototypical MCM-41, but also for other materials, a fraction of functional silane of up to about 20% still results in the formation of well-developed mesostructures, depending on the nature of the organic group^99,115^ but the nature of the phase formed might change at high fractions of organoalkoxysilane.^116^ However, with organically bridged silsesquioxanes, it is possible to produce ordered mesoporous organosilicas (PMOs) with exclusively organically modified silicon atoms.^29–31^ Adjustment of the content of the functional silane and the nature of the pending organic groups allows fine control of the hydrophobicity of the materials.^117^

For co-condensation pathways, there is one feature, which seems to be little explored. The hydrolysis rates of organoalkoxysilanes substituted to different degrees with alkyl groups are very different: under basic conditions, the higher the level of substitution with alkyl groups, the slower the hydrolysis, which is the other way round under acidic conditions.^118^ It would be expected that this would lead to incorporation of organoalkoxysilanes into the framework formed at different stages of the synthesis, which could lead to inhomogeneity of the distribution of organic groups over the material, and thus could affect drug uptake and release properties. This effect, however, does not seem to have been studied in any detail as yet. The difference in accessibility of grafted and co-condensed surface groups, as reported by Rosenholm and Lindén,^119^ may be related to different reactivity of the silanes. Also in drug-release experiments, SBA-15 modified by co-condensation did not perform as well as similar materials obtained by grafting, which may again be related to the location of the functional groups.^120^

It had already been mentioned above, that ordered mesoporous materials containing exclusively silicon atoms bound to one carbon atom are accessible by the condensation of organically bridged siloxanes, such as bis(triethoxysilyl)ethane and related compounds, in the presence of surfactants. Such precursors can be used in co-condensation reactions with tetraalkoxysilane, but also as the sole silicon source. In such cases each silicon atom in the material is connected *via* an organic bridge to one other silicon atom. This means that not only the surface of the solids, but also the bulk within the walls is exclusively organically modified, often resulting in a higher hydrothermal stability. Under well controlled conditions and using bis(triethoxysilyl)benzene as a precursor, it is even possible to synthesize ordered mesoporous silica with a crystalline order within the walls, achieved by benzene stacking.^121^ Generally, the synthesis proceeds as that for purely siliceous ordered mesoporous silica. The surfactant and either base or acid are added to water or water mixed with other solvents, often alcohols; as a next step, the organically bridged silane (pure or as mixtures) is added and the reaction system reacted at a given temperature for a duration of typically several hours. The solid is recovered and the surfactant template is removed, mostly by extraction, but due to the high thermal stability of Si–C bonds and selected organic bridges, in specific cases, using carefully established protocols, also calcination may be possible to at least partially remove the surfactant, while retaining the organic groups in the framework.^122,123^

PMOs have not been as extensively studied for use in drug delivery as their pure silica counterparts (possibly modified with pending organic groups). This has been attributed to the more difficult synthesis of uniform and discrete PMO nanoparticles (NPs) with adjustable properties on the one hand, and lack of information on bio-safety of PMO nanoparticles on the other.^124^ However, in principle, they have favourable properties, such as the adjustable hydrophobicity and the possibility to control biodegradability. This has been extensively covered in excellent recent reviews.^124–126^ Degradation of PMOs with inert bridging groups in water or simulated body fluids, such as ethane or benzene, was found to be remarkably slower than that of pure silica NPs, which was attributed to the high hydrophobicity and the more stable Si–O-bonds in silsesquioxanes.^125^ This higher stability, however, can be modified, if organic groups, which can be cleaved enzymatically or *via* redox reactions, are incorporated in the PMO framework. This is treated in depth in ref. 125. The overall degradation is then a complex interplay between the decomposition of the organic groups and the remaining silica framework.

Moreover, co-condensation of organically bridged silsesquioxanes with alkoxysilanes or alkylalkoxysilanes creates materials with organic functional groups in the bulk of the material, possibly pending organic groups, and silanol groups, which can be further modified by different surface groups. Thus, bi- or trifunctionalized materials, comprehensively covered in ref. 124, are accessible, which broadens the scope of such materials even more. Due to these additional possibilities, it can be expected that PMOs will play a more important role in the field of drug delivery in the years to come.

### 1.3. Synthesis of mesoporous silica nanoparticles

The sections above mainly dealt with the adjustment of the mesostructures and the control of the surface chemistry of ordered mesoporous silica. However, for use in biomedical applications, the morphology of the individual particles is of high importance. While in the early days of research on ordered mesoporous silica the morphology was more or less accepted as obtained from a particular synthesis protocol, it is not sufficient anymore for a number of advanced applications. For drug delivery purposes the drug carrier particles need to be isolated and in the size range of around 10 nm to approximately a few hundred nanometers.^126,127^

The excellent textural properties of mesoporous silica materials together with their ability to be used in drug delivery technology inspired the rapid translation from bulk to the nanoscale dimension. The reason that fueled this transition to the nanoscale could be found in the unique physicochemical properties that mesoporous nanoparticles could offer to drug delivery technologies. Among them, we like to highlight their great pharmacokinetic profile, the improved drug stability and solubility, and their outstanding control over the timing and location of the therapeutic release, which would contribute to reduce the potential toxicity of the therapeutic agent.

In the race of developing mesoporous silica nanoparticles for drug delivery technologies, the contribution of Victor Lin should be highlighted. Even though Prof. Lin passed away in 2010 at the age of 43,^128^ his seminal contributions were essential for the development and applications of mesoporous silica nanoparticles, a term that he coined to illustrate nanoparticles made of mesoporous silica with a well-defined and controllable morphology.^129–132^ He was also able to demonstrate the possibilities of MSNs through their application in a variety of different scientific areas, such as heterogeneous catalysis,^133^ renewable energy,^134^ biosensing^135^ and nanomedicine,^136,137^ among others.

However, strictly speaking, Stöber was the real pioneer in developing the synthesis of spherical monodisperse micron size silica particles.^137^ Since then, many modifications have been made to the so-called Stöber method to yield many different types of monodispersed mesoporous nanosized silica particles. The reason for such popularity relies on the fact that manipulating the reaction parameters might result in materials with different morphologies (such as films, fibers, monoliths or particles) and sizes (macro, micro or nano-particles). In this sense, the first time that the Stöber method was modified towards the production of mesoporous silica particles was carried out by Grün *et al.*, when they introduced a cationic surfactant to produce micrometer spheres of ordered mesoporous oxide MCM-41-like particles.^138^ Then, nano-sized mesoporous silica particles were reported by the research teams of Cai,^139^ Mann^140^ and Ostafin.^141^ And then, Victor Lin was the one who popularized the term MSNs referring to mesoporous silica nanoparticles.^130^ Since then, MSNs with a variety of morphologies, dimensions, pore sizes and pore structure have been explored by many different research groups. The conventional synthesis of MSNs is performed at low surfactant concentration to force the assembly of the ordered mesophases to depend on the interaction between the cationic surfactant and the growing anionic oligomers of the silica precursor, which in turn restraint the assembly of mesophases to small sizes. Once the reaction is complete, the organic surfactant is removed by either solvent extraction or calcination and the silica particles might be isolated. However, the synthesis conditions of MSNs can be modified in different aspects, such as the pH of the reaction mixture, the type of surfactants or copolymers employed as structure directing agents, or the concentrations and different types of silica precursors. There are outstanding reviews in the literature where the principles of the different synthetic methods employed for the formation of various MSNs are introduced and explained, together with their influence on the final properties.^38,127,142–145^

There are several reasons, why for drug delivery applications NPs should be used – although MSNs have not been approved by the US Food and Drug Administration for medical applications, yet.^127^ Such nanoparticles are isolated and can be made colloidally stable, which is crucial when a preparation for medical applications should be stored without settling of the solid fraction. The payload in porous silica NPs can be made high, if porosity is high, the surface can be functionalized almost at will by the methods discussed above, and with proper synthetic protocols, the size can be adjusted to pass certain barriers in the body.^126^ As compared to bigger particles, NPs are more easily degraded and can thus leave the body faster. Biodegradability can be tuned over rather wide margins, so that release rates can be adjusted (although degradation of the carrier is not the only release mechanism).^125,127^ The final degradation product of the silica moiety of the drug carrier is silicic acid, which is physiologically unproblematic and has thus been considered safe by the US FDA for 50 years.^126^

Due to the high interest in MSNs and organosilicas, there is a vast number of review articles. These can certainly not all be listed here, since as of 2017 approximately 350 such review papers were identified.^126^ However, several of these more recent reviews shall be highlighted here, since they are particularly useful and relevant in the field of drug delivery vectors. Some of these originate from the group of one of the authors of this review. In ref. 127, the field is broadly covered, while ref. 146, updated four years later^147^ is focused on stimuli responsive drug delivery systems. Ref. 124 specifically covers organosilica nanoparticles for biomedical applications. Möller and Bein give an overview with an emphasis of different aspects of the synthesis and application of silica nanoparticles, amongst others for use in medicine.^148^ Ref. 149 treats various types of silica NPs for biomedical applications, and MSNs are placed in this context. Sun *et al.* treat different methods and describe in detail influential factors to control the morphology of mesoporous silica, not only to produce NPs, but also other morphologies.^150^ Croissant *et al.* provide broad coverage of biosafety and degradation of MSNs.^125,126^ Two reviews from the group of Lindén address the medical applications with respect to materials properties and also discuss biodistribution and safety.^151,152^ As stated above, this list falls very short of being complete, even with respect to review papers only. However, it allows access to the literature on this field from rather different angles, all with relevance to biomedical applications.

The most versatile method for the synthesis of ordered mesoporous silica combines two well-known approaches in silica chemistry, *i.e.* the so-called Stöber-method for the synthesis of monodisperse silica spheres with sizes in the low hundred nanometer size range, and templating of mesopores by different types of surfactants, as discussed extensively in the previous sections. The versatility of the synthesis of monodisperse, sub-μm sized silica spheres was described in a publication in 1968,^153^ based on earlier observations of Kolbe.^154^ The key element of the Stöber-synthesis is the addition of an alkoxysilane (mostly tetraethoxysilane is used) to an ammonia solution (mostly in ethanol/water with excess ethanol, but other solvent systems are also possible) under agitation at relatively high dilution. This results in the formation of solid monodisperse silica spheres with sizes typically in the range of 100–400 μm, but systems producing spheres somewhat outside of this range are also known. The key ingredient, which induces the formation of monodisperse spheres, is ammonia (ethanolamine and basic amino acids are alternatives), and thus the Stöber method typically proceeds under alkaline conditions. While the Stöber synthesis in the first decades after its description was only known to experts, it became highly popular for various applications around 2000, which recently led Ghimire and Jaroniec to speak about a renaissance of the method in a very useful recent review on this synthesis.^155^ The synthesis is well established, and a hands-on guide for the synthesis of solid and porous Stöber-type spheres can be found in a methods and protocols paper, which focuses on hollow spheres, but contains much useful information also on the regular Stöber-process.^156^ Giesche described in detail the precise adjustment of particle sizes by a seeded growth process, with control of sizes between about 20 nm and 3.5 μm; moreover, description of a continuous synthesis is included as well.^157^

The Stöber-process is a rather robust synthesis, but the addition of surfactants to create mesoporous nanospheres is not straightforward, since conditions have to be adapted. The easiest method seems to be the combination of TEOS and octadecyltrimethoxysilane as silica sources. A reliable recipe for the synthesis of 350–400 μm sized porous spheres is given in ref. 156, and the particle size can be increased by increasing ammonia concentration and decreased by lowering ammonia concentration. Also, this type of synthesis can be carried out in a continuous fashion.^158^ MCM-48 type monodisperse spheres were produced by an adaptation of the Stöber-synthesis with the addition of CTAB as the template, but the spheres are somewhat large for biomedical applications, about 800 nm judging from the micrograph.^159^ This problem was solved later by Kim *et al.*, who controlled particle size by using an additional surfactant, *i.e.* the block copolymer Pluronic F127.^160^ While these silica NPs are typically obtained with spherical morphology, Wang *et al.* reported a synthesis of cubic or truncated cubic NPs, which have a structure similar to *Pm*3̄*n* SBA-1.^161^ They are produced also in an ammonia system, but the TEOS is dissolved in hexane instead of in an alcohol.

A very versatile approach for the synthesis of silica NPs with different particles sizes, pore sizes, pore arrangement and functionalization was reported by Möller and Bein.^148^ The key element in their synthesis is the use of triethanolamine as the base; in addition, small amounts of fluoride are added.

For materials, which are obtained under acidic conditions, there is no such general approach for the synthesis of monodisperse particles in the size range of around 100 nm, since the Stöber-method relies on the use of ammonia or amines as the mineralizer. The prototypical SBA-15 is normally obtained with broad particle size distribution at sizes exceeding 1 μm. Thus, synthesis conditions have to be explored, and for nanoparticle synthesis one needs to fine-tune the system by optimizing the synthesis conditions. Lee *et al.* published a comprehensive study, where various synthesis parameters were explored, resulting in different particle sizes and shapes, including sizes in the range of around 100 nm.^162^ For KIT-6, a synthesis was reported to yield 45 nm particles.^162^ However, the quality of the particles in this publication is difficult to judge, since only atomic force microscopy (AFM) data are shown. Generally, plate like particles could be interesting, since diffusion pathways are short, if the channels are perpendicular to the plate base. However, while plate like particles with thickness in the desired range are accessible, overall particle dimensions typically still exceed 1 μm.^163^ Zhu *et al.* succeeded in synthesizing SBA-15 with dimensions of 400 × 400 nm and narrow particle size distribution by optimizing synthesis conditions, but here also no general procedure was developed for the synthesis of NPs.^164^ Since SBA-15 has interesting properties, there is thus a need in drug delivery for the development of more controlled synthesis processes for the formation of SBA-15 and related materials in monodisperse and nanoparticle morphology.

There is one rather general method that allows the synthesis of surfactant templated ordered MSNs, irrespective of pH, which is evaporation induced self-assembly (EISA). This method had originally been developed for the preparation of coating, but was extended to aerosol-based syntheses to allow production of a wide variety of silica NPs.^165^ Particles are in the right size range, but seem to have a rather broad particle size distribution. Due to the more complex setup and polydispersity of the products, NPs from aerosol-assisted EISA processes do not seem to have been often used for drug delivery applications, yet.

**Fig. 7:**
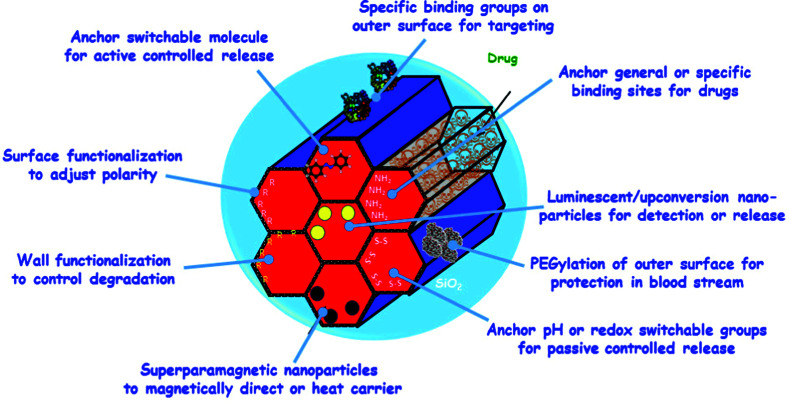
Schematic drawing of some modification options of ordered mesoporous silica to impart additional functionality.

Due to the fact that a high level of control is required for the synthesis of ordered MSNs in any case, these processes are often suitable for the introduction of further functionality with high relevance into drug delivery. Fig. 7 schematically shows the different types of modification, which are useful for biomedical applications; they were recently very well reviewed in ref. 127 and 126. Introduction of organic groups, as discussed above, can modify the polarity of the materials and thus optimize the loading and release of drug molecules.

Organic groups can also be tailored to control biodegradation of the carrier. Magnetic NPs can be integrated to drive particles by magnetic fields or heated magnetically for drug release. Capping groups on the pore mouth allow stimuli-responsive release, for instance, by a pH change or optical switching, which changes the spatial arrangement of molecules. The methods for functionalization are almost limitless, and range far beyond drug release. Magnetically sensitive probes can be incorporated for MRI contrast, and fluorescent molecules, ions, or NPs in order to induce optical response upon stimulation. Moreover, the drug loaded NPs can also be directed to the regions in the organism, where the drug should be applied. Passive direction can make use of the shape of particles, because this may induce a preference for specific organs or cell types.^166^ However, one can also attempt to more actively target the carrier system to the places, where the drug should be administered, and different approaches are discussed in ref. 127.

#### Scale-up

Published synthesis procedures almost exclusively are described for batch sizes of a few grams only. If ordered mesoporous silica should be used in practical applications, scale-up is required. Since biomedical applications typically do not need high amounts of the material, moderate scale up appears to be sufficient to meet demands, at least for the near-term future. Although commercial scale amounts of silica materials for biomedical applications are not yet available, there are a number of indications that this should not pose unsurmountable problems. First of all, a true commercial application of ordered mesoporous silica in a catalytic process started around the year 2000 by Exxon-Mobil, although the details of the process have not been disclosed.^16^ This means that surfactant templated silica, albeit probably not with the kind of particle morphology required for biomedical applications, can be synthesized at commercial scale. On the other hand, Stöber particles are commercially available from several suppliers, and thus this process also has been scaled up to at least moderate amounts. While the combination of the two scaled-up processes to result in the larger scale production of ordered MSNs for biomedical applications is not straightforward, it is probably not impossible, if there were a sufficient demand for such particles.

Moreover, there are reports in the literature, that for selected examples either scale-up of batch syntheses or production using continuous processes is possible. One of the authors of this review recently reported a scale-up to pilot scale (5 L) of the synthesis of MCM-41 suitable for biomedical applications, and while tuning of parameters was required, overall, the synthesis proved to be sufficiently robust in that further scale up can be envisaged.^167^ Continuous solution-phase synthesis of MCM-41 was reported as early as 1998;^168^ however, in this publication the focus was more on the analysis of the early stages of formation, not production of higher amounts. Later on, continuous production set-ups for ordered mesoporous materials were described, which allowed throughput on the order of litres of solution per minute,^169^ or with a synthesis capacity of 330 g per run (limited by the size of the storage vessels for the reagents).^170^ Monodispersity of the product can be a problem in such processes, but this can be solved by using segmented flow reactors.^158^

Finally, the aerosol-assisted EISA process^165^ is a continuous synthesis method by its very nature, so that higher amounts of material can naturally be prepared by extending the synthesis time, if proper collection methods are used.

Thus, on analysing the available information, it can be concluded that scale-up of ordered mesoporous silica of biomedical grade should be possible to serve at least limited markets. For commercialization, one certainly must comply with good manufacturing practice to obtain approval by the respective national authorities, but generally, this should also be possible.

## 2. Mesoporous materials for drug delivery

Ideally, a drug delivery system should control the loading process and the rate and period of release of the corresponding drug as well as specifically directing it to a target tissue or cell.^147,171–173^ There are numerous research studies focused on the development and biomedical application of silica-based mesoporous matrices to host and release various antitumor, antimicrobial or other types of therapeutic agents.^171–175^ The expansion and development of this type of studies were inspired by the pioneering research work of María Vallet-Regí's group on the MCM-41 material as a controlled delivery system with ibuprofen as the model drug.^9,10^

**Fig. 8:**
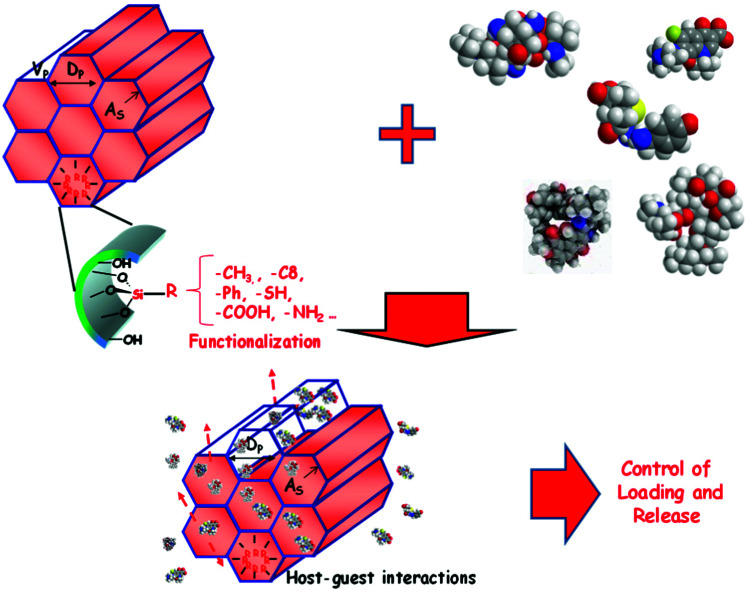
Schematic ilustration of the influence of the textural and chemical properties that make mesoporous materials the best candidates for controlled drug delivery.

The most outstanding properties that make mesoporous materials the best candidates for controlled drug delivery are mainly focused on: ordered pore structure (structural properties), narrow pore size distributions, large surface areas and volumes (textural properties) and high density of silanol groups that facilitate the covalent bonding of organic groups (chemical properties) (Fig. 8).^10,176^

First of all, the biocompatibility of silica based materials has been widely demonstrated in different *in vitro* and *in vivo* models.^177,178^ In terms of structural and textural properties, pore diameter, surface area and pore volume are crucial to obtain the best efficiency of these mesoporous siilica materials. The pore diameter must be adapted to the size of the drug molecules to be loaded and is consequently a limiting factor when choosing and loading the corresponding drug. In addition, the pore diameter acts as a regulator of release rate as it limits the diffusion of drugs along the pore into the release medium.^179^ On the other hand, the surface area of these materials is also a key for a greater amount of drug to be loaded; the greater the surface area, the greater the interaction with the loaded molecules. In addition, in order to increase the amount of drug loaded into the materials, the pore volume and thus the filling of the materials can be increased, inducing an increase in drug–drug interactions within the mesoporous cavities.^101,180^ With respect to the chemical properties, the amorphous silica surface has a high density of silanol groups. In this context, when the surface is hydroxylated to the maximum value, Zhuravlev determined the number of OH groups per unit surface area as a silanol number density, which has the numerical value of 4.9 OH per nm^2^ (arithmetical mean);^101,180^https://doi.org/10.1002/chem.200600226. While for pure silica mesoporous materials the interactions between the material and the drug are through weak interactions with the surface silanols, such as van der Waals forces or hydrogen bonds, it is possible to use the silanols for functionalization to improve the covalent anchoring of various organic groups. This fact allows working with a wide variety of hybrid organic–inorganic mesoporous materials.^98,179^

As discussed above, the functionalization with organic groups of the surface of mesoporous silica materials makes them excellent candidates for drug delivery, since it allows to control the adsorption and release of drugs. There are basically two methods for functionalizing mesoporous silica matrices, the one-step synthesis or co-condensation method and the post-synthesis or grafting method (see above). The former method consists of a single step in the presence of a surfactant as a structure directing agent and involves different simultaneous hydrolysis and condensation reactions of the silica and organosilica precursors. Although this strategy allows the organic functions covering the entire silica surface, there is an upper functionalization limit to avoid the disorder of the mesoporous structure. The second method is usually performed to modify surfaces by grafting organic silanes ((RO)_3_SiR′) under anhydrous conditions. This method allows a great versatility of selective functionalization of both the external surface of the silica and the internal and external surface of the mesopore, before or after surfactant extraction, respectively. As a consequence, this functionalization increases the wall thickness, and the organic molecules present in the mesopore decrease the textural properties of the material.^98^ As is well known, the electronic interactions, host–guest interactions through electrostatic attractive forces, and hydrophilic–hydrophobic interactions present for the different functionalizations of mesoporous matrices allow a controlled and specific modulation of the charge and release kinetics of the correponding drugs.^10,180–182^

## 3. Mesoporous silica nanoparticles for drug delivery

One of the most important aspects for any nanoparticles to be employed in drug delivery technologies, independently of the type of nanocarrier employed, is their capacity to transport their payload to precise locations in the body to increase efficacy and reduce potential side effects. In this regard, during the design of a nanocarrier, it is compulsory to consider its biological behaviour when it might be administered. Thus, it is necessary to take into account the biocompatibility, biodistribution, biodegradability and potential clearance of any engineered MSNs to be used as a drug delivery system.

### 3.1. Degradation, biodistribution and clearance

MSNs have gained the attention of many research groups to be used as drug delivery nanosystems. However, despite all the interest, they have not been approved yet to be used in medical applications by the regulatory agencies. In this sense, there are some prerequisites that need to be addressed before reaching clinical trials, such as the MSN degradation, biodistribution, clearance routes, and their final fate within the body.^183^ This sequence of requirements is not exclusive for MSNs, because the performance of any potential nanocarriers, and the subsequent translation to the clinic, is dependent on the adsorption, distribution, metabolism, and elimination. The area of nanomedicine has included these processes into biokinetics that includes nanoparticle uptake, biodistribution and elimination.^184^

**Fig. 9:**
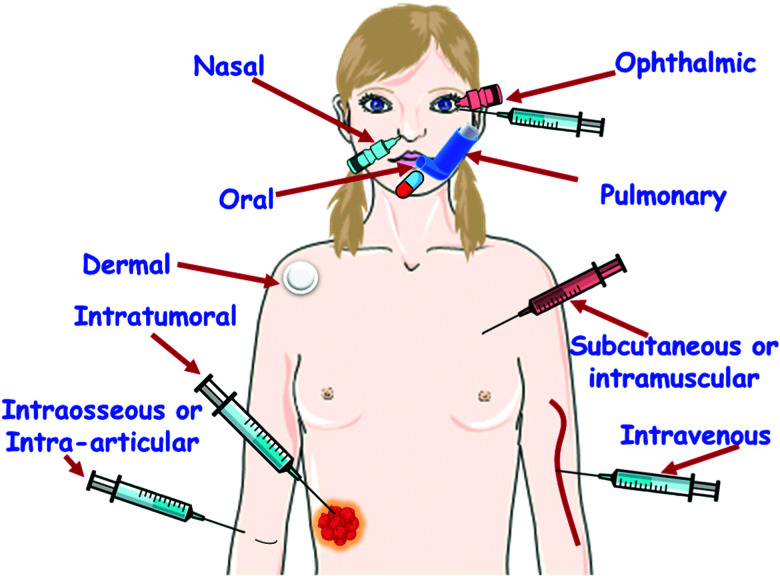
Schematic illustration of all possible routes of mesoporous silica nanoparticle administration for drug delivery.

**Fig. 10:**
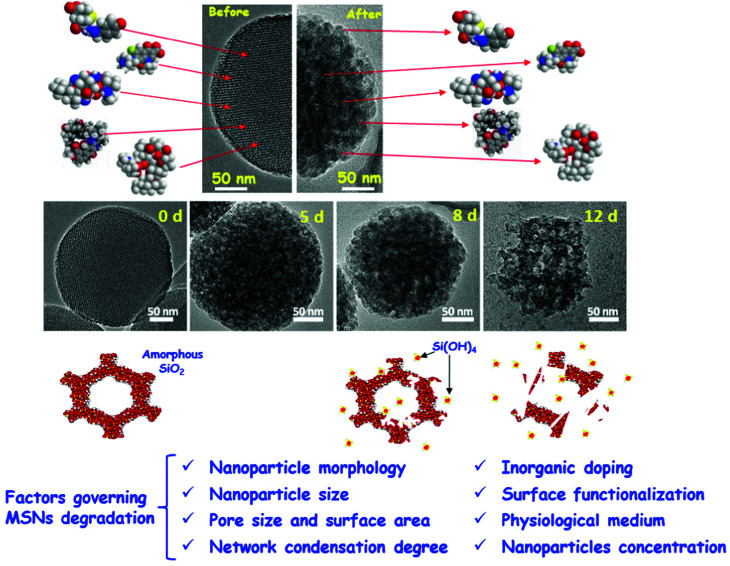
Different transmission electron microscopy (TEM) images of MSN dissolution in phosphate saline solution (PBS) media after different soaking times (0, 5, 8 and 12 days), and schematic illustration of the dissolution process of MSNs in an aqueous medium. The different factors governing MSN degradation are also displayed.^185^ Adapted with permission from ref. 185. Copyright 2017, Springer.

Among all the possible routes of NP administration for drug delivery, the most common are intravenous, subcutaneous, intratumoral, intraosseous and intra-articular (Fig. 9). Therefore, the nanoparticle stability in blood, or any other physiological media, is one of the first requirements that should be addressed. In fact, there should be a balance between nanocarriers being robust enough to protect the payload during the journey and being biodegradable once they have accomplished their mission. This balance can only be reached through detailed understanding of the chemistry of the employed nanocarriers. Although MSNs are known to be mechanically, thermally and chemically stable, their potential degradation through lixiviation of the siloxane bonds from the silica network in aqueous media might present a strong influence on the release kinetics (Fig. 10).^185^ Additionally, the orthosilicic acid by-products of the degradation products are not expected to be cytotoxic since they are biocompatible and excreted through the urine.^125^ In fact, silicic acid has been recognized as safe by the US Food and Drug Administration (FDA) for over 50 years.^126^

#### 3.1.1. Degradation of MSNs

As above-mentioned MSNs are degradable under physiological conditions.^125,185,186^ The silica degradation process can be divided into three steps: (i) hydration, in which water molecules are adsorbed into the siloxane network; (ii) hydrolysis, in which siloxane bonds hydrolyse and produce silanol groups; and (iii) ion-exchange processes, consisting of nucleophilic attack of hydroxyl groups (OH^−^), resulting in leaching of silicic acid. Once the degradation mechanism is known, the next step should be investigating the degradation kinetics, that is, the dissolution rate of the MSNs. Thus, different research studies have focused on evaluating the influence of the parameters that govern the degradation of MSNs (Fig. 10), which are the following: nanoparticle morphology, nanoparticle size, pore size and surface area, condensation degree, inorganic doping, surface functionalization, physiological medium and nanoparticle concentration.^125,185,187^

##### Effect of morphology

The influence of MSN morphology on their degradability has been evaluated. For instance, Hao *et al.*^188^ investigated the degradation of MSNs with different aspect ratios (AR), from nanospheres (AR = 1) to nanorods (AR = 2 and AR = 4), in different simulated media, highlighting simulated intestinal and body fluids. The results indicated that sphere-shaped MSNs exhibited faster dissolution (50–60% of degradation) and the authors of that paper attribute this finding to the relatively larger external surface area of spheres in comparison to rod-shaped MSNs (25–30% and 15% of degradation for short and long nanorods, respectively).

##### Effect of nanoparticle size

The influence of nanoparticle size on MSN dissolution was evaluated by Braun *et al.*,^189^ in diverse simulated fluids. To this aim they synthesized MSNs with diverse diameters, 80, 200 and 1500 nm and proved that the dissolution profile of the nanoparticles was independent of their size. This finding agrees with that obtained by Yamada *et al.*,^190^ who tested the degradation process in colloidal MSNs with various diameters (20, 30, 40 and 80 nm) with comparable surface areas (960–980 m^2^ g^−1^) and quantified the amount of Si species dissolved in the phosphate buffer saline solution (PBS). Again the results showed almost equal degradability rates (15 wt% per day) for the four sizes with a complete degradation in a week.

##### Effect of pore size and surface area

The effect of pore size on the degradation of MSNs has been also evaluated. Hence, larger pores should permit enhanced diffusion kinetics of water in and out of the pores, favouring the silica dissolution process.^125^ In this line, Shen *et al.*^191^ developed dendritic MSNs with radial porosity and well-defined diameters ranging from 2.8 to 13 nm. TEM studies proved that MSNs with 10 nm radial pores underwent complete hydrolysis after only 24 hours.

In the same report, the authors designed hierarchically core@shell MSN@MSN nanospheres (MSN-5 nm pore@MSN-10 nm pore) to lower the degradation rate. The results indicated that the MSN shell exhibiting 10 nm pores degraded during the first 12 h, whereas the degradation of the MSN core of 5 nm pore diameter occurred after 72 h of assay. Although surface areas and pore volumes of MSNs with 10 nm pore diameters were considerably higher than those of MSNs with 5 nm pore sizes, their condensation degree was similar and therefore authors concluded that the degradation was faster in MSNs with 10 nm than in MSNs with 5 nm because of the enhanced diffusion kinetics in the former.

The surface area plays a key role in the dissolution kinetics of MSNs. Actually, the higher the surface area the greater the contact with the physiological fluid at the interfaces, which should produce faster dissolution rates. This fact was proved by Yamada *et al.*^190^ when comparing the degradability in PBS of two different MSNs exhibiting different surface areas, blackberry-like MSNs (303 m^2^ g^−1^) and chrysanthemum-like MSNs (934 m^2^ g^−1^). After different incubation times at 37 °C, samples were observed by TEM, confirming that MSNs exhibiting the highest surface area were the most degraded.

##### Effect of the network condensation degree

The network condensation degree of MSNs is a pivotal parameter that governs their degradability, since it controls the reactivity of the silica network towards hydrolysis. In this regard, the condensation degree can be evaluated by ^29^Si solid state nuclear magnetic resonance (NMR), which typically displays peaks at *ca.* 1110 ppm (Q^4^, corresponding to Si atoms connected by four Si–O–Si bonds) and at *ca.* −100 ppm (Q^3^, corresponding to Si atoms connected through three Si–O–Si bonds and one Si–OH).^191^ In MSNs with a well-condensed silica network there are more abundant Si atoms in Q^4^ than in Q^3^ environments, the latter exhibiting Si–OH groups, which are essential for the post-functionalization of the surface of these NPs. A highly condensed silica network with almost only Q^4^ sites and very low degradation rates can be obtained *via* calcination post-synthesis treatment, resulting in very low dissolution rates. Thus, He *et al.*^192^ proved that only 30% of calcined MSNs were degraded after 15 days of soaking in a simulated body fluid (SBF), while incompletely condensed silica was fully dissolved.

##### Effect of inorganic doping

Diverse authors have studied the effect of the inorganic doping of silica on MSN degradability. Thus, silica doping with zirconium, calcium, iron, manganese and zinc cations and/or oxides strongly influences the dissolution rate of MSNs, as discussed below.

Fontecave *et al.*^193^ demonstrated that mesoporous mixed silica–zirconia oxide nanoparticles exhibited a slower dissolution rate than pure silica MSNs in a PBS medium. Zirconium doping not only inhibited degradation but also produced self-limitation of soluble silica concentration in the close environment of the nanoparticle due to the re-condensation of silica on local zirconium centres in the mesoporous matrix.

Oppositely, calcium-doped MSNs showed faster degradation kinetics than pure silica MSNs, as reported in a pioneering study by Li *et al.*^194,195^ The incorporation of calcium in MSNs decreases the connectivity of the silica network due to the increase in the number of non-bridging oxygens, which produces faster dissolution and degradation rates.

Among inorganic dopants, iron oxide was also incorporated into MSNs to improve the degradability of the resulting nanosystems.^196–198^ The incorporation of iron enhances the silica dissolution owing to two main reasons: on the one hand due to the decrease of the silica condensation degree at the interface of the iron oxide nanophase; on the other hand because the elimination of iron from the mixed oxide network, by either dissolution or chelation, increases the porosity of the silica.

In another research study, Yu *et al.* developed manganese-doped hollow MSNs that exhibited tumour microenvironment-triggered biodegradation.^199^ This “metal ion-doping” approach allowed the accelerated biodegradation of Mn-doped hollow MSNs in either mild acidic or reducing tumor microenvironment by disintegrating the Mn–O bond and successively triggering “manganese extraction”, which promoted the fast biodegradation of Si–O–Si bonds afterward.

Very recently, Chen *et al.*,^200^ developed zinc-doped MSNs as tunable biodegradable nanoplatforms that can deliver therapeutic zinc ions within tumor cells after internalization. Zinc plays both network forming (covalent –Si–O–Zn–O–Si linkages) and network modifying (–SiO^−^·Zn^2+^ moieties) roles and therefore plays a key role in controlling the degradation rate of the nanosystem, particularly regarding variations in the degradation rate *versus* pH. Thus, the acidic pH prompted fast zinc release due to both cation exchange with H^+^ and cleavage of the Zn–O covalent bond, which created hollow MSNs. Nonetheless, at the physiological pH of 7.4, the zinc release consists of a two-step process. The first one consists of a quite fast zinc release due to the cationic exchange of Zn^2+^ by H^+^. The second one relies on the release of the covalently bonded zinc following a slower process that requires the cleavage of Zn–O bonds and subsequent breaking of the silica network, which results in a more uniform and slower degradation of the nanosystem.

##### Effect of surface functionalization

Cauda *et al.*^201^ investigated the influence of the organic functionalization on MSN degradation. In this work, the authors investigated the degradation of non-functionalized MSNs and functionalized MSN-R (R = phenyl, chloropropyl, aminopropyl and PEG) in SBF up to one month. The results evidenced that the fastest degradation took place in phenyl-functionalised MSNs, whereas PEGylation significantly inhibited silica dissolution; meanwhile, it decreased the deposition of an apatite-like layer on the surface of the nanoparticles. In addition, the dissolution process produced an increase in the pore size, a collapse of the porosity and, consequently, a decrease in the surface area of MSNs. In another study, Hao *et al.*^188^ also investigated the effect of PEGylation on MSN degradation. They not only confirmed that the presence of PEG slowed down the degradation of MSNs, but they also observed that there was a modification in the dissolution process itself. Whereas pristine MSNs dissolved from the outermost surface to the inside, PEGylated MSNs began to dissolve from the interior towards the external surface. In another study, Cauda *et al.*^202^ also investigated the influence of the density of the PEG coverage and polymer chain length on the dissolution process of MSNs. To this aim, they covalently grafted to MSNs PEGs of different molecular weights, 550 and 5000 Da, and a mixture of both polymers. The results indicated that the denser and longer the polymer chains the slower the dissolution rate of silica. In this sense, Paris *et al.*^203^ reported similar results regarding the degradation of polymer-coated MSNs, since grafting of MSNs with a stimuli-responsive copolymer slowed down the silica dissolution rate.

##### Effect of a physiological medium

Undoubtedly, the fluid in which the MSNs are soaked plays a pivotal role concerning the stability of silica. Braun *et al.*^189^ investigated the degradation of MSNs in different dissolution media: simulated lung fluid (SLF, pH 7.40), SBF (pH, 7.25), simulated gastric fluid (SGF, pH 1.60) and PBS (pH 7.40). The silica dissolution rates followed the order SLF > SBF ≈ PBS ≫ SGF, indicating that, apart from general pH effects, the presence of organic acids in SLF produced faster silica dissolution rates. Hao *et al.*^188^ also evaluated the effect of the presence of fetal bovine serum (FBS) in Dulbecco's Modified Eagle's Medium (DMEM) on the degradation of MSNs. They found that the presence of proteins from FBS accelerated the silica dissolution process and decreased MSN stability. Although the protein-mediated degradation of silica was previously reported,^204^ this study confirms that this is a relevant factor in MSNs for biomedical applications, since they will be in close contact with many diverse proteins in the biological environment.

##### Effect of nanoparticle concentration

The concentration of MSNs in a given medium also controls their degradation rate, because the silica dissolution is governed by solubility limits.^205–207^ The solubility of amorphous silica is 120 ppm (40 mg L^−1^) in water, at neutral pH, 25 °C and 1 atm.^205^ Nevertheless, there are different parameters, such as the presence of solubilizing agents (nucleophilic amine, hydroxide species, *etc.*), ionic strength and protein concentration, which can modify this threshold value. In a pioneering study, He *et al.*^192^ evaluated the degradation of three concentrations of MSNs, namely 0.1, 0.3 and 0.5 mg mL^−1^, in SBF. The results proved that, independently of the MSN concentration, a three-stage degradation behavior was observed. There was an extraordinarily fast bulk degradation stage in the first two hours and a decelerated degradation stage blocked by the formation of a calcium/magnesium silicate layer followed by a sustained slow diffusion stage on the day-scale. The three experiments produced total MSN degradation in 15 days. However, different proportions of materials were degraded during the initial burst-effect stage, being 35% (0.5 mg mL^−1^), 60% (0.3 mg mL^−1^) and 90% (0.1 mg mL^−1^), which accounts for the solubility thresholds of silicic acid from silica dissolution. The influence of the concentration of MSNs on their degradation provides some significant conclusions derived from the investigations reported to date:^125^ (i) most of the silica dissolution rates reported in the literature cannot be directly compared; (ii) some degradation thresholds reached in some experiments could be attributed to the use of an extremely elevated silica concentration or the use of deionized water; (iii) employing dynamic conditions, *i.e.*, a continuous flow of the degradation media, would rather simulate *in vivo* conditions, but in this case faster and total degradation of MSNs are expected; and (iv) the degradation of MSNs depends on the amount administered, the administration route and its site of action.

#### 3.1.2. Biodegradation and clearance

The next step was evaluating the *in vivo* dissolution or biodegradation of MSNs using different animal models. In most of the cases, the chemical composition of the nanocarriers was identified as a key parameter, because the surface functionalization with polymeric coatings improved their stability and, therefore, increased the bloodstream half-life. In general, it has been observed that MSNs are distributed to body tissues and are excreted primarily *via* renal clearance.^125^ The biodistribution of *ca.* 220 nm MSNs was observed in the liver, spleen and kidneys after 24 hours of intravenous injection.^208^ After 7 days, most of the nanocarriers were excreted from the body without signs of severe tissue damage. The biodistribution of smaller MSNs (50–100 nm) administered by intravenous injection was also evaluated, and excretion *via* hepatobiliary transport was observed.^209^ In similar experiments, the biodistribution studies, which were carried out in small animals, confirmed the MSN accumulation in the reticuloendothelial system (RES), including lungs, liver, and spleen.^210^ The reason for the accumulation in the RES system was attributed to the serum protein adsorption on the surface of the MSNs, which can be avoided through their functionalization with hydrophilic polymers like poly(ethylene glycol) (PEG). It has recently been found that the type of adsorbed proteins would influence the composition of the protein corona and, therefore, the final fate of the nanoparticles.^211^ Thus, it is possible to influence the final biodistribution of the MSNs through the appropriate functionalization, which would in turn strongly influence the protein corona composition.

In a different experiment, the biodistribution of MSNs was also evaluated by radiolabelling them with positron emission tomography detectable ^64^Cu using mice bearing xenografts of breast cancer tumors^212^ and human glioblastoma tumours.^213^ The biodistribution of both models was observed to be similar, independently of the presence a targeting ligand. The highest concentration of MSNs was found in the liver, and much less NPs were found in lungs, spleen, kidneys, and intestines, together with a low concentration in the blood. More importantly, both independent studies demonstrated almost identical biodistribution, regardless of the animal model used.

Similar biodistribution experiments were carried out labelling differently sized MSNs with ^89^Zr and ^45^Ti, finding that the higher the particle size (from 80 to 160 nm), the higher the accumulation in the spleen and lower in the liver.^214,215^ Similarly, fluorescently labelled 80 nm MSNs were employed for evaluating the biodistribution in healthy mice.^216^ In this case, the highest concentration of NPs was observed in the spleen, although they were also found in the lungs and liver. The same experiment revealed the effect of particle size and functionalization on the nanocarrier's biodistribution. As expected, the functionalization of MSNs with PEG led to a longer blood circulation time, regardless of the size of the nanocarriers. On the other hand, non-functionalized large MSNs presented a shorter blood half-life, which could be attributed to their accumulation in the liver and spleen. The same effect of modifying the surface of ^89^Zr MSNs with PEG was observed in mice with xenografts of LNCaP and PC-3 tumours. The non-modified MSNs were rapidly accumulated in the lungs, liver and spleen while the PEG modified nanocarriers showed a longer blood half-life.^217,218^ On the other hand, liver accumulation was observed when MSNs were decorated with cationic species, such as amine groups, which could be due to the fast protein accumulation onto the cationic surface of MSNs.^209,219^

As it has been mentioned above, besides size and surface functionalization, particle shape was found to have a strong influence on MSN biodistribution. MSNs with different shapes, spherical and elongated, were evaluated. The biodistribution analyses revealed that elongated and cylindrical particles accumulated in the spleen and presented a shorter blood half-live in comparison with spherical particles.^166,219,220^

The injection route has also been observed to be an important parameter in the biodistribution of MSNs. In this sense, MSNs when injected *via* subcutaneous injection require additional time to enter and circulate in the blood stream, as expected.^221^

The clearance routes of MSNs from the body were evaluated through different studies that confirmed the renal elimination as their main excretion route.^125,166^ Among these reports, the seminal investigation carried out by Tamanoi and coworkers showed that although MSNs were initially found in the spleen and liver, most of them were renally excreted after 96 hours of administration, and a minority were excreted through feces.^222^ In this sense, it was found that renal clearance required a certain level of particle dissolution^223^ although other reports have also found intact MSNs in the urine.^166,222,224^

With regards to other potential excretion routes, hepatobiliary excretion has been observed through the liver and bile, which is governed by protein adsorption on the particle surface,^225^ and excretion through the faeces was found to be favoured by nanoparticle aggregation.^226^ The excretion route has been found to be connected with the blood circulation time of MSNs.^227^ In this sense, the longer the blood circulation time, as in the case of PEGylated MSNs, the slower the clearance rate. Additionally, as it has been commented above, the PEG moieties grafted on the surface of MSNs can reduce their dissolution rate, which delay the clearance of the MSNs from the body, as expected.^202^

In general, the physicochemical properties of MSNs strongly affect their biocompatibility. Therefore, depending on the final application, the MSNs should be designed with appropriate characteristics, and the administration route and dose should be carefully selected, so that good biocompatibility, low toxicity and controlled biodegradation and clearance would be guaranteed.

### 3.2. Targeting

As it has been mentioned above, the design and engineering of MSNs should include the ability to deliver the therapeutic cargo to precise locations. In this sense, the majority of the research on targeted NPs has been focused on the potential treatment of cancer, because these nanocarriers can selectively accumulate in tumour tissues to locally release their payload. When NPs are injected into the bloodstream, they preferentially accumulate in the tumour through what is called passive targeting. This is due to the particular architecture of the tumour's blood vessels, which have extensive fenestrations. In addition, these tumour tissues usually lack effective lymphatic drainage, so this is why this phenomenon of preferential accumulation is known as enhanced permeability and retention (EPR) effect,^228^ as it will be described below in the cancer section.

However, sometimes the efficiency of the EPR effect is not as high as expected for treating a disease such as cancer.^229,230^ A potential alternative that can be employed together with the EPR effect is the active targeting, which is based on surface conjugation of molecular targeting ligands that might present high affinity towards specific membrane receptors overexpressed in the cell membranes. This approach enables the development of nanocarriers that are able to recognize biomolecules associated with specific disease conditions, such as cellular receptors overexpressed on the surface of cancer cells. Although targeted NPs have not yet reached the clinic, there are several approaches that are under investigation, and even some clinical trials are currently underway.^231^ In this sense, there are several reasons that could explain some of the major disappointments of targeted nanocarriers, such as the immunological response due to the presence of the surface ligands, the increase of size due to the presence of voluminous ligands, or the binding site barriers, that occur when ligands bind with high affinity to target molecules so that further diffusion into the tissue is hampered. This is the reason why a balance between binding affinity and diffusion ability should be targeted in the design of nanocarriers.

Although targeted nanocarriers have not yet reached the market, they have been employed by many different researchers worldwide to increase the accumulation of MSNs in tumours using different targeting agents, as it will be reviewed below in the cancer treatment section. In fact, the different targeting approaches should be designed based on the tumour type to be treated.

### 3.3. Stimuli-response

**Fig. 11:**
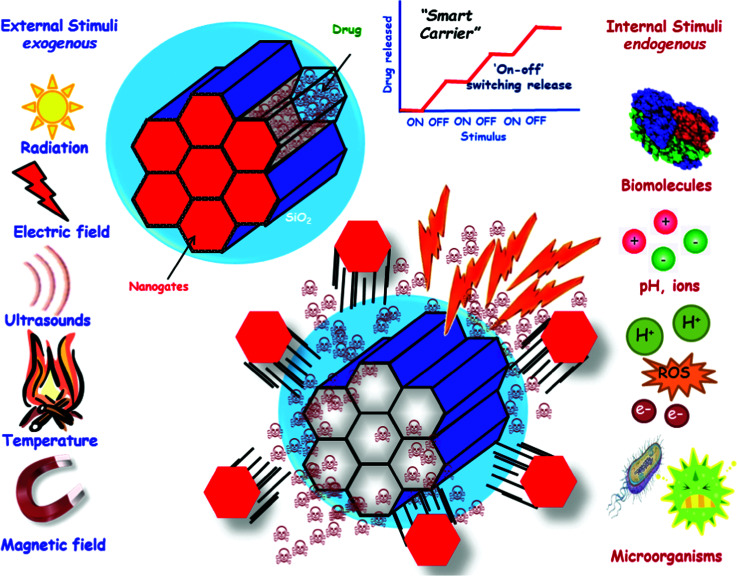
Schematic representation of stimuli-responsive MSNs for triggered drug release.

One of the great advances of nanomedicine is the potential for triggered release of therapeutic agents in the tissue of interest. As described above, a suitable drug delivery system should meet a number of essential requirements to be used as a nanomedicine in the clinic, such as being able to encapsulate a high amount of therapeutic agents, being able to transport them to the specific tissue of the targeted disease avoiding any premature loss of the drug, and being able to release a large amount of the transported drug or biomolecule to achieve a high therapeutic concentration at the local level. It is thus desirable for any ideal nanomedicine to be able to release its payload on demand, *i.e.*, in response to a provided stimulus, either an internal stimulus characteristic of the pathology to be treated or an external stimulus controlled from the outside by a physician. Both modalities have advantages and disadvantages, as will be described throughout this review. For instance, in the treatment of cancer, there are several stimuli in the tumour microenvironment than can be exploited as payload release triggers (Fig. 11), such as low pH, high concentrations of glutathione or elevated levels of certain enzymes. On the other hand, there are certain materials, such as inorganic nanocarriers, that can transform energy coming from external sources into heat that can be employed for therapy. The external energy source can be near-infrared light, ultrasounds or magnetic fields, that can be controlled from the outside and can be localised to a specific area reducing the potential side effects.

With respect to MSNs, their possible stimuli-sensitive behaviour is of particular interest, since their open porosity makes it relatively easy to introduce drugs into their pores. On the other hand, it is also very easy for these therapeutic agents to escape through the same way they entered. This would greatly reduce their efficiency and could increase certain side effects due to the massive distribution of certain drugs throughout the body. Therefore, it is necessary to devise different strategies to close the pore gates to prevent premature release of the cargo. Over the last few years, various strategies have been developed to close the pore gates depending on the pathology to be treated, as we will see in the following sections of this review. The present review will focus on smart nanocarriers based on MSNs capable of releasing high local concentration of the therapeutic cargo on-demand after the application of certain stimuli, such as endogenous stimuli (pH, redox, enzymes and small molecules) and/or exogenous stimuli (light, magnetic field, temperature and ultrasound).

## 4. Potential biomedical treatments using MSNs

### 4.1. Cancer

Cancer is a very complex disease that is characterised by an abnormal and uncontrolled cell division that leads to tumours that could spread to the surrounding tissues. In the last few decades, cancer has been ranked as one of the leading causes of death worldwide, with an estimated 20 million new cases and 10 million deaths worldwide only in 2021.^232^

Cancer is more than just one disease since there are many different types of cancer depending on the part of the body where it might start, and it could spread throughout the body in a process called metastasis. In an attempt to classify cancer, there are two main categories: hematologic or blood cancers, which are those of the blood cells, and solid tumour cancers, which are those of any of the other body organs or tissues. In the former cancer, nanotechnology is expected to play a key role in the promising area of immunotherapy and the development of CAR T cell therapy, while in the later cancers, nanocarriers are already being employed in the clinic as an alternative to conventional chemotherapy.^231^ In any case, cancer is very heterogeneous and complex, and this is why developing effective cancer therapies is a very challenging process.

Conventional cancer treatments include surgery, radiotherapy and chemotherapy, which would depend on the type and stage of the cancer to be treated. Conventional chemotherapy is based on the systemic administration of drugs that target rapidly-growing and dividing cells. Their mechanism is commonly based on blocking key metabolites needed for replication, intercalating into DNA, competing with selected nucleotides or blocking microtubule development. However, these conventional anticancer drugs are commonly highly cytotoxic, poorly soluble in aqueous media, have low stability and bioavailability and, in most cases, they lack of specificity. All of these pitfalls often lead to severe side effects, which might include pain, cardio toxicity, diarrhoea, nausea, hair loss and potential depression of the patient immune system. Consequently, the dosage needs to be reduced, which might result in lower therapeutic effect and development of resistance to these drugs.

#### 4.1.1. Nanoparticles for cancer treatment

There are some therapeutics that were very promising in the lab, but failed on their way to the clinic because of several reasons, such as limited water solubility, low stability, off-target toxicity, drugs that induce adaptive resistance or impossibility of overcoming the several barriers associated with cancer. In this regard, research on improved delivery techniques has been developed to offset many of these concerns. In fact, there is a great opportunity and expectation for tailored delivery systems able to improve certain characteristics of the already existing pharmaceutical compounds, such as their biostability and bioavailability, among others. Among the delivery systems, NPs have changed medical therapeutics and drug delivery for ever.^233^ In this sense, research in cancer nanotechnology has flourished dramatically over the last 20 years. The so-called area of nanomedicine has brought together many different investigators, such as physicists, chemists, engineers, clinicians and biologists to give birth to a multidisciplinary field centred on cancer care and treatment. The reason for the high promise of NPs for cancer treatment relies on the facts that they can (1) show improved safety behaviour, which allows larger doses that would minimise off-target toxic effects and increase the desired therapeutic effects delivered to the cancer cells; (2) increase the delivery time; (3) offer combination treatments with a potential synergistic effect; (4) combine different targets simultaneously; (5) be potentially used in immunotherapy; and, (6) reduce drug resistance through the targeting of certain multidrug resistance proteins.

Therefore, thanks to the use of NPs as drug carriers, certain treatments have been improved. That is the case for liposomes containing doxorubicin, called Doxil, which was the first approved liposomal nanomedicine by the FDA and showed fewer adverse effects than the free drug for Kaposi sarcoma and ovarian cancer treatment.^234,235^ Another example is paclitaxel (a potent anticancer drug) containing NPs that can be administered using much less toxic drug solvents than the free drug. In general, many FDA-approved nanomedicines, such as Doxil, Abraxane Marqibo, Onyvide, Vyxeos, and others, have drastically reduced the life-threatening toxicities of the free-drug, improving the safety and efficacy of existing drugs^236^ and, consequently, resulting in a moderate improvement of the overall survival of the treated patient.^237^

In general, NPs bring the potential of improving the solubility and stability of the anticancer drugs encapsulated, favour their transport across membranes and biological barriers, and increase the circulation time in the bloodstream, which improves their safety and efficacy.^238^ All these characteristics allow many limitations typical of conventional therapies to be overcome.^239^ There are many different types of NPs that have been approved for clinical use by the FDA^240^ or that are under investigation. Among them, the most popular nanocarriers are based on lipids, polymers, or inorganic materials, such as gold, iron or silica NPs.

Lipid-based NPs (LNPs) offer many advantages, such as their simple formulation, their high biocompatibility, and great bioavailability. Additionally, their physicochemical characteristics can be easily modified to modulate their biological response. All these reasons have placed LNPs at the top of FDA-approved nanomedicines,^241^ including the COVID-19 vaccine formulations.^242^ However, despite the excellent properties of LNPs, they are still limited by their storage stability and the relatively low payload that they can deliver.

Polymeric NPs are considered as good delivery vehicles thanks to their biocompatibility, precise control of the final properties, simple formulation parameters and stability during storage. Their versatility allows the delivery of a great variety of the potential cargo, such as hydrophobic or hydrophilic drugs, small molecules, large biomolecules, proteins or even vaccines.^243^ However, polymeric NPs present some disadvantages, such as the increased risk of particle aggregation and potential toxicity. In fact, the number of polymeric nanomedicines currently accepted by the FDA is lower than one might expect.^240^

Inorganic materials, including gold, iron or silica NPs, have also been employed to produce nanocarriers with a wide variety of sizes, structures and geometries.^244,245^ However, even though gold and iron based NPs might present unique plasmonic or magnetic properties, their clinical application has been limited by their low solubility and potential toxicity concerns.

Among the available inorganic NPs, those made of silica have attracted the attention of researchers in the area because of their robustness. For example, silica quantum dots (C-dots from Cornell University) are ultrasmall silica nanoparticles of 6–10 nm, so they can be cleared through the kidneys avoiding potential accumulation in the body. Cornell dots are in clinical trials for cancer imaging in patients with metastatic melanoma or malignant brain tumours (NCT03465618, NCT01266096 and NCT02106598).^246,247^ The clinical trials have demonstrated promising preliminary results, since those C-dots are well tolerated without significant side effects, preferentially taken by the tumour and efficiently cleared by the kidneys. However, they cannot be employed for cancer drug delivery treatment, because their cargo adsorption capacity is very limited,^248^ although they hold great expectations for detecting, staging and biopsy of tumours with great accuracy.^247^ Another type of silica-based NPs, MSNs, has become very popular worldwide due to the outstanding morphological properties to be used as drug delivery systems for the potential treatment of cancer. Compared to the above described organic NPs, MSNs show the typical advantages of inorganic materials, such as mechanical strength, tuneable degradability, and thermal and chemical stability.

The potential clinical success of MSNs in the treatment of cancer depends on (1) their stability in physiological environments and their circulation time in the bloodstream; (2) their capability to cross the many different physiological barriers to reach the affected anatomic sites; (3) their capacity to accumulate at the disease site; (4) their safe behaviour; and, (5) their optimised pharmacokinetic behaviour.

#### 4.1.2. Stability and circulation time of MSNs

Nanoparticle stability is a vital prerequisite for any formulation to be employed in the biomedical field. The circulation time of any nanoparticle in the body depends on the physicochemical properties of the nanocarriers, such as NP size, shape, surface chemistry, and surface properties. Consequently, the biological performance of MSNs, taking into account their biocompatibility, biodistribution, biodegradability and clearance, is governed by the same set of properties.^249^

##### MSN size

Size is very important to increase the circulation time and stability of MSNs. In general, it is recognised that any nanoparticle with a diameter smaller than 10 nm is rapidly eliminated by the kidneys, while the NPs with a diameter larger than 200 nm might be eliminated by the reticuloendothelial system. Regarding the particular case of MSNs, the preferred size to ensure long circulation half-time is 50–300 nm.^146,250^ As mentioned above, the lower limit relates to the potential fast-renal clearance, while the upper limit refers to the potential embolisms provoked by MSN aggregation into capillaries and alveoli. In general, it has been accepted that MSNs with a diameter below 100 nm might slow down the best possible levels of cellular intake. As a matter of fact, the optimal size range of MSNs to secure long circulating half-life is 50–100 nm because smaller MSNs are difficult to produce due to their inherent mesoporosity, and larger MSNs would present difficulties in bypassing certain biological barriers.^146^

##### MSN shape

During their circulation in the bloodstream, NPs might suffer different flow rates that could potentially include shear stress that could damage them. In this sense, it has been observed that the shape of the carriers could have a strong influence on the final fate of the NPs. Typically, spherical NPs have been employed in nanomedicine, but other morphologies, such as rods, cylinders, disks or ellipsoids, have also been explored because of their different performance regarding their biocompatibility, biodistribution and potential clearance.^220,251^ Regarding the MSN shape, some reports have evaluated the *in vivo* fate of rod-like MSNs, revealing that short-rod MSNs were preferentially taken up by the liver while long-rod MSNs were preferentially retained in the spleen.^220^

##### MSN surface properties

Any circulating MSNs would come into contact with many different types of biomolecules and cells suspended in blood. They thus come into contact with a highly complex fluid that can contain an average of more than 3700 proteins, among other biomolecules, such as sugars, cholesterol, aminoacids, *etc.* Thus, a corona on the surface of the MSNs could be formed due to the non-specific adherence of serum proteins and lipids.^252^ Consequently, the composition of the corona would depend both on the types of biomolecules that might be present in the blood and on the MSN surface. In fact, small modifications of the particle surface could have a strong influence on the adsorption or desorption of proteins and, subsequently, on the corona composition.^253^ From the variety of proteins present in blood, a family of proteins called opsins stick to the surface of foreign bodies to favour the detection and fast removal by the macrophages from the mononuclear phagocyte system. In this sense, attaching hydrophilic polymers to the external surface of MSNs has been widely employed to provide stealth properties to those NPs.^147^ As a general trend in this area, many NPs incorporate poly(ethylene glycol) (PEG) on their surface to avoid rapid excretion. Coating MSNs with PEG does not only increase the dispersion of the particles in the suspension media,^202^ but also shields the MSN surfaces from enzymes and antibodies, interfering with the opsonisation process and enhancing their circulating half-life.^201,254–257^ The molecular weight of the employed PEG together with the packing density of PEG grafted has been observed to play an important role in the repulsion effects against opsins, finding a range of 10 000–20 000 molecular weight of PEG grafted to MSNs as an optimum.^255^*In vivo* evaluation of PEGylated MSNs demonstrated a decreased accumulation in the liver, spleen and lungs, while the circulation in blood half-life increased and biodegradation decreased, as initially expected. These findings were in agreement with the smaller amount of degradation products found in urine of PEGylated MSNs *versus* non-PEGylated MSNs.^166,255^

However, our daily exposure to PEG containing additives in products for skin care and cosmetics has been found to increase the production of anti-PEG antibodies that could induce a fast clearance of PEGylated NPs.^258^ Consequently, an alternative to PEGylation has been developed through the use of Zwitterionic surfaces, that are characterised by presenting the same number of both positively and negatively charged functional groups on their surface, which preserves the electrical neutrality.^259,260^ This approach was evaluated by covalently grafting zwitterionic polymers with positive and negative groups within their backbone into the surface of MSNs; however, the hydrodynamic diameter was increased too much to employ this platform as a drug nanocarrier.^261^ A potential solution was presented using small zwitterionic moieties to decorate the surface of poly(ethyleneimine) coated core@shell Fe_3_O_4_@MSNs.^262^ This platform achieved a reduced degree of protein adsorption while the co-delivery of different biomolecules was not impeded.

It is also possible to simultaneously graft two different functional groups, positive and negative, to the surface of previously prepared MSNs.^263^ The mixed-charge pseudo-zwitterionic surfaces ensured a significant reduction in protein adhesion and macrophage uptake by MSNs similar to that of PEGylated MSNs.

Other approaches to create “stealth” properties in circulating MSNs include coating them with blood cell membranes to avoid macrophage-based recognition.^264^ This approach was reported for the first time back in 2011, when the surface of poly(lactic-*co*-glycolic acid) NPs was coated with the membrane from red blood cells.^265^

Red blood cells are an excellent choice as membrane source because they present a great ability to circulate in blood for long periods of time, something that is highly desirable in NPs for drug delivery. Since then, a wide variety of cells have been employed as membrane sources to coat different types of NPs. In this sense, MSNs have been also coated with red blood cell membranes and loaded with doxorubicin and a near-infrared photo-sensitizer such as chlorin e6.^145,266^*In vitro* results indicated that the combination of these NPs with laser light stimulation produced synergistic effects of chemotherapy and photodynamic therapy. Similarly, cancer cells have been used to coat MSNs with homotypic targeting ability, thanks to the MDA-MB-231 cancer cell membranes, and magnetic hyperthermia properties, thanks to the superparamagnetic ferroferric oxides encapsulated.^267^ Thus, it was possible to develop cancer cell membrane-coated MSNs for the combination of chemo and magnetocaloric therapy of cancer cells.

An alternative to blood cell membrane coating has been developed through the coating of MSNs with an artificial lipid layer, in the so-called protocells.^268^ This lipid layer on the surface of MSNs prevents protein adsorption and opsonisation.

#### 4.1.3. Tumour accumulation and targeting of MSNs

As it has been mentioned above, most of the conventional drugs employed in cancer chemotherapy are highly toxic, which leads to several side effects that could force the suspension of the treatment. To overcome this problem, nanocarriers can be designed to accumulate within solid tumours and once they reach the target, they should be selectively internalised into tumour cells.^269,270^ Although recent receptors have shown that, on average, less than 1% of the nanocarriers administered into different tumour models reach their targeted tissue,^271^ the data were not normalised by tumour weight, which might distort the values calculated and their pharmacological meaning.^236^

**Fig. 12:**
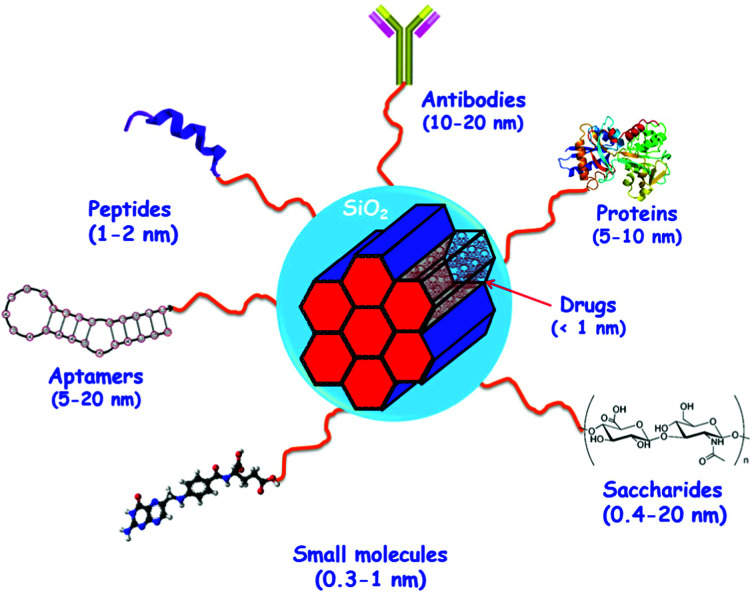
Schematic representation of different targeting ligands attached to the surface of MSNs for active targeting of cancer tissues and cells.

As it happens with most of the available nanocarriers, MSNs provide a great window of opportunity to deliver selectively the anticancer drugs to cancer cells through either passive targeting, active targeting, or a combination of both. Passive targeting has been widely employed by already FDA accepted nanomedicines, and it is based on the enhanced permeability and retention (EPR) effect, that was initially described by Matsumura and Maeda back in 1986.^272,273^ These researchers found that large macromolecules tend to accumulate in the tumour interstitium for long periods of time. As a consequence of the rapid growth of solid tumours, new blood vessels are rapidly formed, exhibiting some imperfections as a consequence of the fast process, such as discontinuous epithelium and several fenestrations. These fenestrations in the defective tumour blood vessels favour permeability that lead to the extravasation of the blood components to the tumour interstitium. Additionally, the rapid growth of tumour tissues also provokes their defective lymphatic drainage, which hinders the diffusion of NPs and large macromolecules, and favours their retention in the tumour interstitium. On the other hand, active targeting consists of attaching certain ligands to the nanocarrier surface, as represented in Fig. 12, that present a great affinity towards some receptors overexpressed in the surface of target organs, tissues, cells or organells.^270^

As expected, the targeting capacity relies on different parameters, such as the abundance and accessibility of the overexpressed receptors in the target, the targeting ligand density grafted on the MSN surface and, of course, the affinity between both targeting ligands and overexpressed receptors. Among the great variety of different host–guest interactions, amide formation and maleimide–thiol coupling are by far the most exploited targeting systems.^146,274,275^ There are many possible combinations of targeting ligands that have been grafted to MSNs for developing nanoplatforms for potential cancer therapy (Table 2), such as the following:^276^

**Table 2:** Different cellular receptors overexpressed in tumour cells and targeting ligands employed to decorate MSNs for potential targeted cancer treatment

Receptor^a^	Targeting ligand^b^	Cell line targeted^c^	Ref.
Proteins			
TfR	Transferrin	PANC-1, BT-549 HeLa, HuH-7, HT1080, MiaPaCa-2	279–286
EGF-R	EGF	HuH-7	287
Sialyl-Lewis X antigen	Aleuria aurantia lectin	DLD-1	288
HER2	Rec. GST-HER2-Afb	SK-BR3	289
TEM1	TEM1-scFv	Ovcar5	290
Sialic acid receptor	ConA	HOS	291

Peptides			
α_v_β_3_ integrins	c(RGDyK)	U87-MG	292
α_v_β_3_ integrins	cRGD	MDA-MB 435	280
Unknown	SP94 peptide	Hep3B	293 and 294
Iα,β receptors	TAT peptides	HeLa, MCF-7/ADR	295–298
α_v_β_3_ integrins	K_7_RGD; c-RGDFK	HeLa	299
IL-13Rα2	IL-13 peptide	U251	300 and 301
Unknown	KALA peptide	A549	302
α_v_β_3_ integrins	N_3_GPLGRGRGDK-Ad	SCC-7, HT-29	303
α_v_β_3_ integrins	K_8_(RGD)_2_	U87-MG	304
—	pHLIP	MCF-7, A549, U20S, H1299, HepG2	305
α_v_β_3_ integrins	Thiolated-RGD	A375, HepG2, MCF-7, Neuro-2a	306
α_v_β_3_ integrins	N_3_RGDFFFFC	U-87 MG	307
NRP1, NRP2	t-Lyp-1	MDA-MB 231, HUVEC	308
α_v_β_3_ integrins	RGD family GFLGR_7_RGDS	U87-MG, MDA-MB-435, MDA-MB-231, HepG2, Neuro-2a, HeLa, SCC-7, HT-29, A375, MCF-7	309
nAChR	CDX	BCEC	310
CD13	NGR family	BCEC, C6	311 and 312
Formyl pept recept 1	Bld-1	HT-1376, T-24	313
Electrostatic	Polylysine	HeLa	314
Melanocortin	NAPamide	Melanoma cell lines	315
CD44	cA6	MDA-MB-231, SK-BR3	316
GRP78	GRP78P	4T1	317

Antibodies			
HER2/neu	Anti-HER2/neu	BT474, MCF-7 SK-BR3	318–320
Mesothelin	Anti-ME1	MM	321
Mucin 1 glycoprotein	Anti-Mucin 1, TAB-004	4T1, MMT	322
CD105/endoglin	Anti-TRC105	HUVECs, 4T1	214
Transferrin (mouse)	Ri7	bEnd5, Neuro-2a	323
HER1	Anti-EGFR	A549	324
CD45R	B220	CALM-AF10	325
HER2	Trastuzumab	SK-BR3, BT474	326–328
PSMA	anti-FOLH1	LNCaP	329
VEGFR2	anti-VEGFR2	FRO	330
EGFR	Cetuximab	SW620	331

Aptamers			
Thrombin	TBAA_15_C_18_	HeLa	332
Nucleolin	NCL/aptamer AS 1411	MCF-7, MDA-MB-231, SW480, PANC-1, PC3	333–336
HER2	HB5	SK-BR3, MCF-7, MDAMB-231	337
EpCAM	Anti-EpCAM	HepG2, SW480, SW620, HT-29, HEK-293T, Y79, WERI-Rb1, Ramos B	336 and 338–340
CD105	YQ26	HEK-293, HUVEC, 4T1	341
MUC-1	ApMUC-1	MDA-MB-231, C26, A549, MCF-7, CHO-K1, A2780, MDA-MB-231	342–344
PTK-7	Sgc8	CCR-CEM	345
HER2	HApt	MCF-7, SK-BR3 cells	346

Saccharides and polysaccharides			
Mannose receptor	Mannose	MDA-MB-231	347
ASGP-R	Lactobionic acid	HepG2	348
Galactose receptor	Galactose	HCT-116, Capan-1, MDA-MB-231	349
CD44, RHAMM, CD168, HARE	Hyaluronic acid	HCT-116, MDA-MB-231 HepG2, HEK-293, Ovcar8, HeLa	350–356
Lectin receptor	Glucuronic acid	HCT-116	357

Small molecules			
FR-α	Folic acid	HeLa, HepG2, PANC-1, U20s, MDA-MB-231, SK-BR-3, MiaPaca-2, MCF-7	222, 224 and 358–369
FR-α	Methotrexate	HeLa	364
Sigma receptor	Anisamide	ASPC-1	370
Electrostatic	TPP cations	Mitochondrion	371 and 372
SA-R	Phenylboronic acid	HepG2, 4T1	373 and 374
NET	MABG	NB-1691	375 and 376
BR	Biotin	HOS, HeLa, MDA-MB-231	377–380
TCII-R	Vitamin B12	No cells tested	381
Electrostatic	Guanidinium cations	Mitochondrion	382

a Receptors (alphabetical order): ASGP-R: asialoglycoprotein receptor; BR: biotin receptor; CD13 (aminopeptidase N, APN): cell membrane alanyl aminopeptidase; CD44 (P-glycoprotein 1, Pgp-1): multifunctional cell surface adhesion receptor; CD45R (PTP): protein tyrosine phosphatase; CD105 (Endoglin): type I membrane glycoprotein; CD168 (RHAMM, HMMR): hyaluronan-mediated motility receptor; EGF-R: endothelial growth factor receptor; EpCAM: epithelial cell adhesion molecule; FR-α: folic acid receptor; GCR: glucocorticoid receptor; GRP78: glucose regulated protein 78; HARE (Stabilin-2): human hyaluronan receptor for endocytosis; HER1 (EGFR, ErbB1): human epidermal growth factor receptor 1; HER2: human epidermal growth factor receptor 2; HER2/neu (ErbB2, CD340): tyrosine–protein kinase receptor; Iα/β: importins α and β; IL-13R-α2: interleukin-13 receptor α2; MUC-1: human mucin 1 glycoprotein; nAChR: nicotine-acetylcholine receptor; NET: norepinephrine transporter; NRP1 and NRP2: neurophilins 1 and 2, coreceptors for vascular endothelial growth factor (VEGF); PSMA: prostate specific membrane antigen; PTK-7: protein tyrosine kinase-7; SA-R: sialic acid receptor; TEM1: antitumor endothelial marker 1; and TCII-R: transcobalamin 2 receptor.

b Targeting ligands (alphabetical order): anti-EGFR: epidermal growth factor receptor antibody; anti-EpCAM: anti-epithelial cell adhesion molecule RNA aptamer; Anti-FOLH1: monoclonal antibody, clone C803N, against human folate hydrolase 1 (FOLH1); anti-HER2/neu: anti human epidermal growth factor receptor 2 (ErbB2, CD340) antibody; anti-ME1: anti-malic enzyme 1 (ME1) antibody; anti-mucin 1, TAB-004: TAB-004 anti-mucin-1 antibody; anti-TRC105: anti-endoglin (CD105) antibody; anti-VEGFR2: anti-vascular endothelial growth factor receptor 2; ApMUC-1: mucin 1 aptamer; B220: Anti-CD45R (B220) antibody; Bld-1: bladder 1 (CSNRDARRC) peptide; CDX: d-peptide ligand of nicotine acetylcholine receptor (nAChRs); cA6: A6 (CKPSSPPEECW) cyclic peptide; ConA: concanavalin A; c(RGDyK): cyclo(Arg–Gly–Asp–d-Phe–Lys) peptide; c(RGD): cyclic RGD (Arg–Gly–Asp) peptide; c-RGDfK: cyclo(–Arg–Gly–Asp-D–Phe–Lys) peptide; Dex: dexamethasone; FA: folic acid; Glu: glucose; GRP789: glucose-regulated protein 78 binding peptide; HB5: DNA aptamer based on 86 nucleotides that binds to HER2; HApt: anti-HER2 aptamer (HApt, 5′-RS-S-C_6_-GCA GCG GTG TGG GGG CAG CGG TGT GGG GGC AGC GGT GTG GGG-3′); K7RGD: linear RGD peptide sequence with 7 consecutive lysine residues; K8(RGD)2: cationic peptide containing 2 RGD sequences; Ri7: anti-insulin receptor substrate 4 (IRS-4) antibody; MABG: metaaminobenzyl guanidine (meta-iodobenzylguanidine analogue); NAPamide: Ac-Nle-Asp–His-D–Phe–Arg–Trp–Gly–Lys-NH_2_ peptide; NCL/aptamer AS 1411: Nucleolin (AS 1411) aptamer; NGR: Asn–Gly–Arg peptide; GRP78P: glucose-regulated protein 78 binding peptide; pHLIP: 38-aa peptide named pH (low) insertion peptide; Rec. GST-HER2-Afb: recombinant glutathione-S-transferase-HER2-affibody protein; Sgc8: aptamer: Sgc8 that binds specifically to human acute T lymphocyte leukemia cells (CCRF-CEM); TBAA15C18: 5′-GGTTG GTGTG GTTGG AAAAA AAAAA AAAAA-C_18_-spacer-3′ modified thrombin binding aptamer; TEM1-scFv: antitumor endothelial marker 1 single-chain variable fragments; Tf: transferrin; t-Lyp-1: truncated form of the cyclic tumor-homing peptide LyP-1 (CGNKRTRGC); TPP: triphenylphosphonium.

c Cell lines: blood: Ramos: Burkitt lymphoma; bone: BMSC: bone mesenchymal stem cells; HOS: human osteosarcoma; bladder: HT-1376: bladder carcinoma; T-24: bladder carcinoma; brain: BCEC: brain capillary endothelial cells; NB-1691: neuroblastoma; U87-MG: human primary glioblastoma; U251: human astrocytoma; breast: BT474: human breast cancer cell line; BT-549: human breast carcinoma cell line; MCF-7: human breast cancer cell line; MCF-7/ADR: human breast cancer (doxorubicin resistant); MDA-MB-231: human breast adenocarcinoma; SK-BR3: human breast adenocarcinoma cell line; cervix: HeLa: human cervix epithelioid carcinoma; intestine and colon: C26: human colorectal cancer cells; DLD-1: colon adenocarcinoma; HT-29: human colorectal adenocarcinoma; HCT-116: human colon carcinoma; SW480: colon adenocarcinoma; SW620: colon adenocarcinoma; epithelia: HT1080: human fibrosarcoma; HUVEC: human umbilical vein endothelial cell line; eye: WERI-Rb1: human retinoblastoma; kidneys: HEK-293: human embryonic kidney 293 cells; liver: HepG2: human hepatoblastoma derived; HuH-7: human hepatoma; lungs: A549: human lung carcinoma; pancreas: MiaPaCa-2: human pancreatic carcinoma; PANC-1: human pancreatic carcinoma, epithelial-like cell line; ovarian: A2780: human ovarian cancer cells (paclitaxel resistant); ovcar: human ovarian serous adenocarcinoma; prostate: LNCap: androgen-sensitive human prostate adenocarcinoma; PC3: human prostate cancer; skin: A375: human amelanotic melanoma; MDA-MB-435: amelanotic melanoma; SCC-7: squamous cell carcinoma; thyroid: FRO: anaplastic thyroid carcinoma; murine lines: bEnd5: mouse brain endothelioma; C6: *Rattus norvegicus* brain glioma; CALM-AF10: murine acute myeloid leukemia; CHO-K1: Chinese hamster ovary; 4T1: *Mus musculus* mammary gland tumor; MMT: murine mammary epithelial cancer cells; and Neuro-2a: mouse neuroblastoma.

– Proteins, some of which have been observed to be involved in the fast metabolism of certain tumour cells. The high demand of certain proteins by tumour cells normally leads to the overexpression of specific receptors for those proteins. Some examples that have been employed to target those malignant cells include transferrin, which is involved in the iron transportation into cells, and epidermal growth factors, that are known for promoting cell growth and differentiation.

– Peptides, whose smaller size than that of regular proteins in terms of number of aminoacids has resulted in a number of certain advantages as targeting ligands, such as easier surface attachment process and better stability in physiological media. Additionally, they are normally accessible products in terms of price, and they would not trigger the immune response of the organism.

– Antibodies, which have been found to be some of the most effective targeting ligands thanks to their specificity. However, they are very sensitive to harsh reaction conditions, so they are normally grafted to the surface of MSNs through electrostatic interactions or covalent grafting employing soft conditions. The major concern of using antibodies for decorating nanocarriers is the potential triggering of the immune response of the patient. This response could be avoided by decorating these nanocarriers with additional hydrophilic ligands, such as PEG moieties, to make them stealth.

– Aptamers: RNA or DNA strand that could fold into specific conformations to specifically recognise different receptors with high affinity. Their attractive properties as targeting ligands, such as small size, high specificity and ligand affinity, have fuelled their use with many different types of nanocarriers.^277^

– Saccharides and polysaccharides, which have been used to provide control on the protein corona and as targeting ligands in many different types of NPs, since some tumours are known to overexpress carbohydrate receptors.^278^ This is the case of hyaluronic acid, which has been employed as a targeting ligand of nanocarriers because tumour cells overexpress specific surface receptors for this polysaccharide.

– Small molecules, which can be conjugated to the MSN surface with a higher ligand density than macromolecules. Additionally, those small molecules are usually stable and present low immunogenicity. An example of this type of ligands is folic acid, a vitamin employed by certain enzymes during the replication of DNA. Consequently, rapidly growing and dividing cancer cells overexpress folate receptors on their surface to capture lots of folate acid. Therefore, decorating MSNs with folic acid has been widely carried out to increase the nanocarrier uptake by cancer cells.

In the last few years, many research groups in this field have combined some targeting elements on one nanocarrier, leading to the so-called double targeting approach.^383^ Different approaches of MSNs with double targeting abilities have been developed using small molecules, such as folic acid with dexamethasone,^384^ with triphenylphosphonium^385^ or glucose,^386^ and peptides, such as arginine–glycine–aspartate peptides with the cell penetrating peptide TAT,^387^ with interleukin 13^388^ or with folic acid itself,^389^ to ensure the specificity and selectivity towards cancer cells.

#### 4.1.4. Overcoming physiological barriers with MSNs

Recent research on nanomedicine-based anticancer drugs has revealed that the major obstacle for increasing their efficacy lies in two basic facts: the poor penetration capacity of carrier NPs and the inadequate balance between drug retention in the bloodstream and drug release at the precise tumour tissue.^390^ This is due to some physiological barriers that NPs should overcome for becoming a successful treatment. Some of the barriers include the fast bloodstream clearance, the lack of selective accumulation in tumour tissues, the absence of selective cellular uptake by cancer cells, or potential endosomal entrapment. In fact, some authors claim that today's minimal therapeutic impact of NPs on drug delivery in the clinic is a direct consequence of their inability to overcome some of these barriers.

Then, once the NPs might have preferentially accumulated in the tumour, thanks to the above mentioned stability and circulating half-life together with the passive or active targeting, there are still some biological barriers to overcome. Among them, the dense extracellular matrix of tumours rich in collagen content hinders the penetration of nanocarriers, limiting their therapeutic effect to the external layers of the tumour.^238^ The lack of nanocarrier penetration and distribution throughout the tumour matrix reduces the potential therapeutic effect, confining it only to the tumour periphery.^391^ This limitation can be addressed through the degradation of the dense extracellular matrix of the tumours to favour the nanocarrier diffusion into the tumour. The matrix degradation can be achieved through the local administration of certain proteolytic enzymes before adding the NPs, or these proteolytic enzymes can also be grafted to the surface of the NPs. Thus, decorating MSNs with different proteolytic enzymes, such as collagenase, has contributed to the digestion of collagen-rich tumour mass, improving the nanocarrier diffusion into deep areas of the malignant tissue.^392–394^ However, these enzymes could be degraded in physiological environments, so a similar approach was developed but using pH-responsive polymeric nanocapsules containing collagenase grafted to the MSN surface. The enzyme would travel protected into those capsules, and the mild acidic conditions typically from lactic acid accumulation in tumour tissues would trigger the release of collagenase only at tumour tissues.^324^

**Fig. 13:**
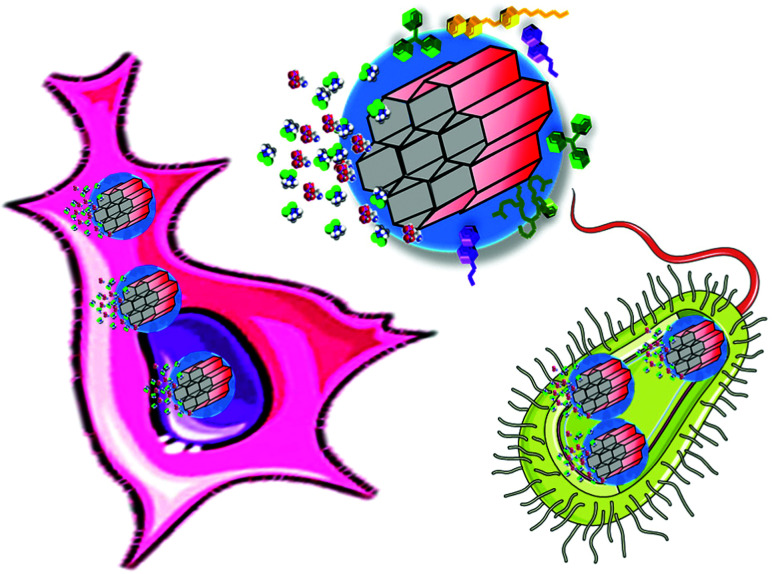
Schematic representation of mesenchymal stem cells and bacteria as carriers of MSNs for enhancing penetration in a tumour context.

Another important physiological barrier of tumour tissues that reduces the therapeutic efficiency of nanotherapeutics is the elevated interstitial fluid pressure. This is a consequence of the rapidly growing and dividing cancer cells, which constrict the lymphatic vessels in the vicinity of the tumour tissue and, therefore, obstruct the drainage hampering the diffusion of the nanocarriers into the tumour tissue.^395^ Recently, the active role of living systems as alternative carriers of drug delivery systems has been explored to solve these biological issues.^396–398^ Hence, living systems with self-propelled and guiding capacities, including mesenchymal stem cells,^399,400^ and non-pathogenic bacteria,^401^ have been envisioned as carriers of MSNs that are able to penetrate tumour tissues (Fig. 13).

The next physiological barrier that NPs might encounter on their journey is the subcellular targeting, that is, arriving at the relevant compartments within the cells.^402^ After travelling through the bloodstream, accumulating in the tumour and selectively internalised into tumour cells, NPs should be able to release their cargo inside the cells. However, when the cells internalise these NPs, they do it through an endocytosis process, in which the endosome is acidified by the action of proton pumps. The nanocarriers would end up being encapsulated into acidic endosomes or lysosomes. Consequently, the highly acidic environment in the endosomes or lysosomes might degrade the nanocarriers and their payload. A possible way to avoid that degradation is based on the proton sponge effect that might cause the endosome to burst. Basically, NPs might be decorated with certain ligands with a buffering capacity, so when the cell encapsulates these NPs into endosomes or lysosomes and send hydrochloric acid to degrade whatever is inside, these NPs are able to buffer that solution. Therefore, the cell keeps influxing protons, together with chloride ions and water, trying to acidify the solution to degrade the endo-lysosome content. Consequently, the endo-lysosome would swell and eventually the vesicle would be disrupted and NPs would be released into the cytoplasm.^403^

Therefore, researchers in this field have decorated the surface of MSNs with buffering ligands to favour endosomal escape and cytoplasm release of the payload, such as poly(amidoamine) dendrimers,^404^ metalorganic complexes,^405^ poly(ethyleneimine) cationic polymer^406,407^ imidazole groups^408^ and certain aminoacids with buffering capacity.^409^ The endosomal escape of nanocarriers can also be achieved through the decoration of their surface with functional groups that can generate reactive oxygen species (ROS) upon external triggering. These generated ROS induce the peroxidation of the endo-lysosomes membranes, favouring their permeability and destabilisation, which leads to the subsequent escape of the encapsulated NPs. Researchers in the area have explored this possibility through different avenues, such as loading a photosensitiser such as indocyanine into the network of cavities of MSNs.^410^ Then, upon near-infrared irradiation, ROS species are generated, destabilising the endosome's membrane. In a similar approach, the photosensitiser can also be grafted to the surface of MSNs to allow the release of the cargo into the cytoplasm of the targeted cell.^411^ The ROS generating species have also been used in the release mechanism from MSNs, such as cleaving ROS-responsive bonds,^412^ so the endosomal escape takes place at the same time as that of the payload release.^413^

In some cases, the payload delivery must be in a particular organelle, as it is the case for mitochondrial targeting because mitochondria are involved in cell apoptosis, cell metabolism and ROS generation.^414^ Among the different approaches, MSNs have been decorated with different mitochondria-targeting ligands, such as triphenylphosphine derivates,^371,415–418^ guanidium derivates^382^ or certain mitochondria-targeting peptides.^419^

Finally, cargo loaded NPs could also target the nucleus of the cells to avoid any potential exocytosis of the cargo before reaching the nucleus. To achieve this, MSNs have been decorated with a particular type of peptides that can recognise specific transport receptors that can initiate the trans-nuclear membrane penetration mechanism^420–423^ The nucleus has been also targeted using gold nanoclusters that show outstanding nuclei staining properties. The staining ligands were composed of gold–protein clusters and were released at acid pH and were able to stain the nuclei, which might be of interest for future cancer cell imaging and diagnosis.^424^

#### 4.1.5. Safety of MSNs

As it has been mentioned above, any nanoplatform employed as a drug delivery system should be characterized according to the regulatory agencies regarding the adsorption, distribution, metabolism, excretion and toxicity properties.^425–427^ Additionally, different MSN parameters, such as diameters, shape or surface properties should be analysed to assess their safety. In fact, these parameters can strongly influence the particle aggregation, protein adsorption at the surface of the NPs and interactions with the physiological environment.

A very interesting study was carried out to explore the MSN—body interaction regarding the adsorption, distribution, excretion and toxicity of these nanocarriers with four different administration routes: oral administration, or intravenous, hypodermic, or intramuscular injection to mice.^428^ The study revealed that the MSNs administered through intramuscular and hypodermic injections could cross certain biological barriers into the liver, but they presented a low adsorption rate. When the NPs were orally administered, they were adsorbed by the intestinal tract and persisted in the liver. As expected, unmodified MSNs administered through intravenous injection were observed in the liver and the spleen.

Regarding the biodistribution of MSNs, as it holds for any nanoparticle systems, they tend to accumulate in the RES organs, that is, in the lungs, liver and spleen. An excellent review of the different modifications that have been carried out to MSNs and their biodistribution and excretion routes can be found somewhere else.^227^ However, the number of biodistribution studies is still quite limited, and the variations of the experimental design, including the type of MSNs (size, shape, and surface chemistry), their different dissolution rates, their mechanical stability, the different protein corona compositions, the different routes and doses administered, and the different animal models employed, make it very difficult to stablish clear MSN design criteria. On the other hand, it is well-established that to increase the circulation time of MSNs, they should be functionalized with hydrophilic polymers, such as PEG, and their size should be controlled to be within the range of 100–150 nm. In any case, optimal design criteria of MSNs to achieve the best possible biodistribution and clearance rates, and therapeutic profile have not been established yet.

#### 4.1.6. Pharmacokinetics of MSNs

Nanomedicines in general can exhibit a wide range of different *in vivo* kinetic characteristics, depending on the design of their formulations. The design of ideal nanocarriers for cancer therapy should include the ability of releasing high local concentrations of the therapeutic payload at the tumour site, as it has been commented above. Additionally, that payload should be released on-demand, that is, after the application of certain stimuli, in what are called smart drug delivery nanosystems.^429^ These smart delivery systems are very interesting when the payload is a cytotoxic drug, as is often the case in cancer therapy, because this can avoid the premature release of the transported cargo before reaching the tumour site. This on-demand feature improves the therapeutic efficiency of the nanocarriers and reduces the potential off-target side effects of the cytotoxic drug.

The stimuli-responsive concept is of great importance when the employed nanoplatforms are MSNs. Their open porous structure favours the introduction of therapeutic molecules into the network of mesoporous, but it is also very easy for the payload to diffuse out of the porous channels before reaching the targeted tissue. Therefore, the pore entrances might need to be closed once the payload is encapsulated within the pores. Thus, this approach allows producing nanocarriers with zero premature release of their payload, thanks to the grafting of different organic or inorganic species on the pore outlets that might work as pore blockers or gatekeepers.

**Fig. 14:**
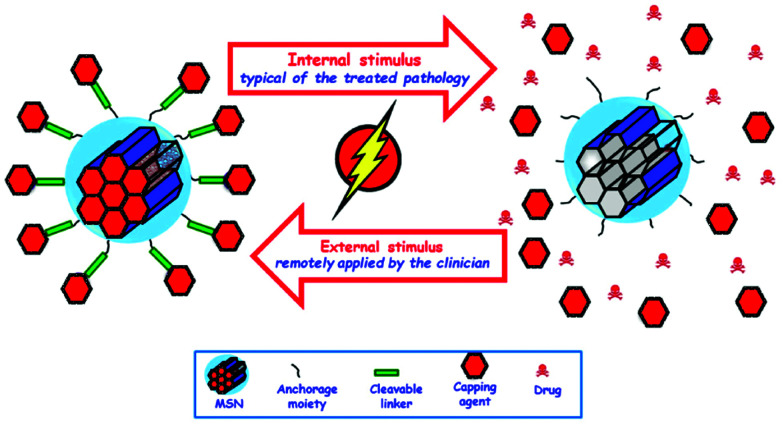
Schematic representation of the different internal and external stimuli employed for triggering drug delivery from MSNs.

Those pore gates could can be closed using different types of gatekeepers, such as (1) reusable gatekeepers, that are based on a bulky capping molecule that can bind reversible to the surface of MSNs; (2) completely reversible gatekeepers, which are based on the reversal concept of a ring shaped macromolecule that closes or opens a stream or a flux; and (3) irreversible gatekeepers, which are based on the cleavage of certain chemical bonds of the capping molecules, leading to a permanent separation of that molecule from the pores’ entrances.^430^ Consequently, the stimuli-responsive behaviour of the MSNs can be developed through the attachment of cleavable bonds or certain gatekeepers that might undergo either chemical or physical changes in response to selected stimuli (Fig. 14).

From the perspective of the type of stimulus that can trigger the cargo release from MSNs, there are two fundamental types of stimuli: internal and external stimuli.^127,146,431–434^ Therefore, the goal of those smart MSNs is to transport the therapeutic drug to the tumour tissue and once at the target site, the action of a certain stimulus, either internal or external, might trigger release of their payload at the precise site where it is needed, achieving a great control on the administered dose.

##### Internal stimuli

**Fig. 15:**
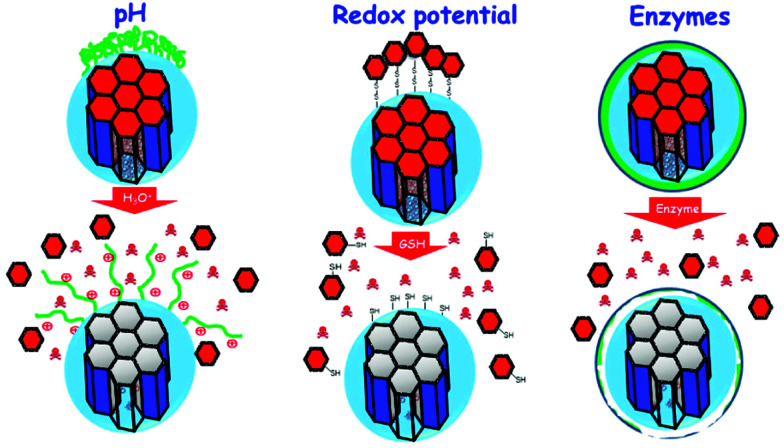
Schematic representation of different internal stimuli employed to trigger drug release from MSNs.

Internal stimuli are those typical for the treated pathology. In this sense, these smart MSNs are designed to respond to certain chemical variations that might take place as a consequence of the disease. In fact, these carriers are also known as self-regulating drug delivery systems, because they are able to release their therapeutic cargo in response to certain chemical or biological stimulation present in certain places of the human body without any external intervention by the physician. Fig. 15 describes the different internal stimuli employed to trigger drug release from MSNs.

##### pH responsive MSNs

Among all the available stimuli employed to trigger the release of the cargo from MSNs, pH is one of the most employed internal stimuli.^291,435–445^ The reason for such popularity can be found in the different pHs that some pathologies present in comparison with healthy situations. This is, for instance, the case for the extracellular pH of tumour tissues, which is between 6.5 and 7, as compared to the physiological pH, 7.4. This lower pH of tumour tissues is due to the Warburg effect, where cancer cells produce their energy through high rate of glycolysis, leading to the production of acidic lactate.

Additionally, there are also differences of the pH in the different cell compartments or organelles: the cytosol presents a pH of 7.4, while Golgi apparatus (6.4) and other residues, such as endosomes (5.5) or lysosomes (5.0), are more acidic. In general, pH-responsive MSNs can be designed employing different mechanisms for capping the pore entrances, such as using polymers, macromolecules or inorganic moieties. Researchers in this field have employed pH sensitive polymers that are able to change their conformation depending on the environment. Thus, those polymers would not allow the payload release at the physiological pH, while an acidic pH would change their conformation and the cargo release would be triggered. Examples of the polymers grafted to the surface of MSNs used as pH-sensitive shells include poly(4-vinyl pyridine),^411,446^ poly(2-(diethylamino)ethylmethacrylate,^447,448^ poly(2-(dimethylamino)ethylacrylate,^449^ chitosan,^357,450,451^ starch, poly(allylamine hydrochloride) and polystyrene,^452^ coordination polymers,^444^ gelatin,^453,454^ polymeric lipids^455^ and different poly(acrylic) responsive polymers.^456,457^ Besides their conformational changes at different pHs, these polymers with carboxylic acid groups, which can be protonated or deprotonated depending on the pH, have also been employed for pH-sensitive systems. A cytotoxic drug, such as doxorubicin, with amine groups deprotonated at physiological pH, would be linked to the proton of the polymer coated MSNs through electrostatic interactions. When the nanocarriers reach the acidic environments, the carboxylic groups of the polymer would be protonated and the amine groups of the drug would be positively charged, increasing the electrostatic repulsion and triggering the drug release.^458^ This approach has also been employed using poly(l-glutamic acid),^459^ poly(l-histidine)^409^ and poly(aspartic acid).^460^ Similarly, polymers grafted to MSNs through acid labile linkages have been employed for the development of pH-responsive MSNs.^461^ A very innovative type of monomers, self-immolative polymers, that disassemble from head to tail into their monomers upon the application of an appropriate trigger, have also been employed for developing pH-responsive MSNs.^462^

Macromolecules to mechanically interlock the pores of MSNs that allow the cargo release at certain pHs have also been employed for developing pH-responsive nanocarriers.^432,463–466^ Examples of this type of macromolecules are curcubit[6]uril,^467,468^ curbubit[7]uril^469^ and cyclodextrins.^435,440,470–474^

Certain inorganic compounds have also been employed to reversibly block the mesopores’ gates of MSNs for developing pH responsive nanocarriers. These inorganic blocking caps include certain nanostructures, such as Au,^475,476^ Fe_3_O_4_^477^ or CeO_2_^478^ NPs, that are grafted to the pore entrances through pH labile linkages^475,477,479^ or pH dependent hybridisation of DNA strands.^307,439^ Inorganic clusters that decompose at certain pHs have also been employed, such as ZnO quantum dots^480,481^ hydroxyapatite,^482^ Au NPs^483^ or layered hydroxide nanosheets.^484^

In addition to the above mentioned approach of closing the pore entrances with different pH-sensitive moieties, it is also possible to graft drugs to MSNs using pH-sensitive cleavable linkages, such as hydrazine, acetal or ester bonds.^485^ The acidic environment of tumour tissues or endo-lysosomes would provoke the cleavage of the pH-sensitive linker triggering the release of the payload only in those acidic environments.^486–491^

##### Redox responsive MSNs

Glutathione (GSH) is a reducing agent that plays a vital role in the maintenance of the adequate levels of reactive oxygen species (ROS) within cells. Although the levels of GSH vary throughout the organism (in some intracellular compartments the GSH concentration could be 1000 times higher than that in the extracellular media), it is known that tumour tissues might present higher GSH concentration than healthy tissues.^492^ This imbalance in GSH concentration has been exploited to design redox-responsive release systems and more particularly, redox-responsive MSNs for smart drug delivery.^130,136,303,348,493–503^

As it happens in the case of pH sensitive carriers, redox-responsive MSNs can be designed employing different species to close the pore entrances, such as polymers, inorganic moieties or organic species. For instance, different polymers crosslinked by GSH-cleavable S–S (disulphide) linkages have been covalently linked to MSNs as shells.^498,503,504^

It is also possible to graft some other polymers to the surface of MSNs through GSH-cleavable disulphide linkers.^348,494,495,500,505,506^ In both approaches, the high concentration of GSH typical of tumour tissues would cleave the S–S bonds (both crosslinkers and NPs linkers), triggering the payload release from the pores of MSNs.

Similarly, researchers in this field have employed inorganic species, such as CdS,^130^ Fe_3_O_4_^501^ or Au NPs^136,361^ to close the pore entrances using disulphide linkages. The presence of GSH molecules would cleave the S–S bond by reduction to two –SH groups, resulting in the removal of the nanocaps from the pore entrance and triggering the release of the cargo. The pore entrances have also been blocked with metallic nanoconstructs, such as MnO_2_ coatings.^507^ Thus, the high concentration of GSH would reduce those coatings to Mn^2+^, dissolving manganese oxide and triggering the release of the payload.

Certain organic moieties large enough to work as blocking caps, such as folic acid, some antibodies, certain macromolecules, or even dendrimers and proteins, have been linked to the surface of MSNs through disulphide linkers^508,509^ to develop redox-responsive MSNs. Nucleic acids can also block the pore entrances when grafted to MSN through disulphide linkers.^510^ Then, reducing agents could cleave the disulphide bonds, releasing both the payload from the mesopores and the nucleic acid that was acting as the gatekeeper. In the same way, cytochrome *c* has been employed as a pore-capping agent and a therapeutic agent against liver tumour when it was grafted to the surface of doxorubicin loaded MSNs through disulphide linkers.^511^

A smart approach for developing redox-responsive MSNs has been developed in the last few years through the linking of highly cytotoxic drugs or sensitive proteins to the surface of MSNs through GSH-cleavable disulphide bonds.^512–514^ Similarly, inactive prodrugs have been grafted to the surface of MSNs, which could be activated later in the intracellular environment.^515,516^

##### Enzyme responsive MSNs

The dysregulation of certain enzymes, both hypo- or over expression, in certain pathologies or diseased tissues can be exploited for developing smart drug delivery systems.^517,518^ Thus, different enzyme-responsive MSN drug delivery systems have been developed in the last few years.^309,373,469,519–537^

As it has been mentioned above in other responsive MSN delivery systems, enzyme responsive MSNs can be designed by employing different concepts, such as polymer shells, supramolecular interlocking of the pores, or even using enzymes themselves. Thus, different polymers have been employed to coat MSNs bearing specific enzyme-cleavable sites, such as galacto-polysaccharides^536,537^ and hyaluronic acid,^418,469^ whose degradation takes place when certain enzymes might be overexpressed in some tumour cells. Similarly, gelatin, collagen and certain designed polypeptides, glucanamide and polyester derivatives, have been employed to close the pore entrances of MSNs, which would release their payload in response to overexpressed metalloproteinases, amidases or esterases typical of tumour tissues.^519,520,532,534,538^

In a different approach, certain cyclodextrins and glycoluril macrocyclic molecules have been employed as supramolecular nanovalves mechanically interlocking the pore entrances through enzyme cleavable sites.^531,535,539^ Thus, the overexpression of these enzymes would cleave those sensitive linkers, uncapping the pores and triggering the payload release only in the area where the enzyme was overexpressed, that is, the tumour site. Other macromolecules employed for blocking the pores of MSNs include adenosintriphosphate (ATP) that forms a dense network around the particles.^524^ The presence of acid phosphatase, an enzyme employed as a biochemical marker for the presence of bone metastases, would hydrolyse the phosphate bonds, disassembling the shell and triggering the cargo release.

##### External stimuli

**Fig. 16:**
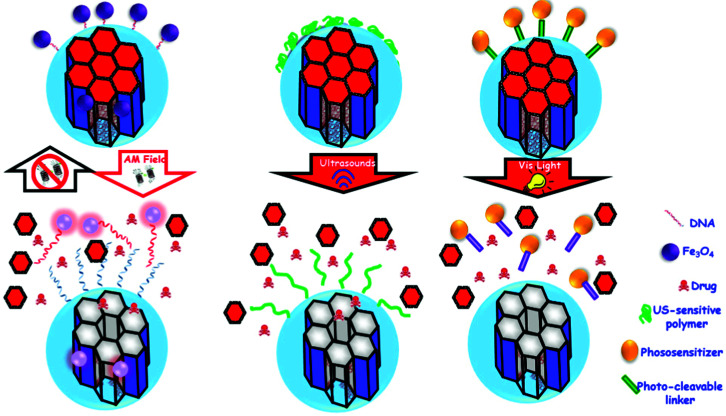
Schematic representation of different external stimuli employed to trigger drug release from MSNs.

External stimuli are those that can be activated remotely by the physician, so the release might be under control at all times. In fact, in some of these externally triggered systems, the release can be switched on and off on demand, which leads to pulsatile responsive drug nanocarriers. One additional advantage of this type of external triggering, is the possibility of local application at the site of the disease, which increases the precision and, therefore, the efficacy and efficiency of the potential treatment. MSNs have been developed to respond to several external stimuli, such as magnetic fields, light or ultrasound, as will be detailed below (Fig. 16).

##### Magnetic fields

Magnetic fields have widely been employed to develop responsive MSNs because they can be used for magnetic guidance under a permanent magnetic field or for a temperature increase under the application of alternating magnetic fields.^540–543^ In the former approach the presence of magnetic NPs within the silica network can be exploited to track the silica nanoparticle fate^544^ or to favour a magnetically guided accumulation in the targeted tumour tissues.^545^ The later approach is based on encapsulating magnetic NPs within the silica network, so they would be able to generate thermal energy under the application of an external alternating magnetic field. These magnetic NPs are normally based on superparamagnetic iron oxide NPs (SPIONs), that can transform the magnetic energy into thermal energy through two mechanisms: Brownian fluctuations, that are a direct consequence of the rapid rotation of the nuclei, and Néel fluctuations, that are caused by the rotation of the magnetic moments.^546^ The most common synthetic pathway of this type of responsive MSNs is through the introduction of SPIONs within the silica network, and then placing thermo-sensitive gatekeepers at the pore entrances, such as certain thermo-responsive polymers that might present a closed conformation at a physiological temperature but an open conformation when the temperature increases by a few degrees.^540^ Other thermo-responsive coatings employed following the same principles are lipid bilayer coatings that would disrupt and trigger the payload release as a consequence of the heat produced by the magnetic field application,^541^ or temperature sensitive pseudorotaxanes that would disassemble under the administration of alternating magnetic fields.^547^

##### Light

Light is a very attractive alternative to be used as stimulus for triggering the cargo release from MSNs because it is non-invasive and allows for a spatiotemporal control of the release.^548–556^ Other great benefits of light as the stimulus include the fact that the wavelength of the radiation can be selected from different regions (ultraviolet, UV, visible or near-infrared, NIR), its easy application, low toxicity, and potential of focusing the light at a precise site of disease.^557^ Thus, UV light was employed to induce the reversible release of the payload from MSNs, the pores of which were blocked with coumarin moieties that underwent dimerization in response to the UV light.^558^ In a similar approach, azobenzene molecules were grafted in the inner part of the pore entrances, and UV light irradiation provoked the *cis*–*trans* isomerisation in such a way that they worked as molecular impellers propelling the payload to come out of the mesopores.^549^ Azobenzene molecules have also been employed to block the pore entrances of light responsive MSNs.^551,559,560^ Other approaches have been developed blocking the pore entrances with different voluminous moieties through a light sensitive linker,^561–565^ or closing the pore entrances with polymers built with light sensitive monomers.^555^

On the other hand, and depending on the wavelength selected, light might present a low tissue penetration capability,^566^ requiring the use of certain medical devices similar to those employed for laparoscopic procedures. In this sense, the use of two-photon-excited photodynamic therapy, in which two relatively low-energy photons are absorbed simultaneously by the photosensitizer, leading to a total energy absorption that is twice that of each absorbed photon, has become very popular for the treatment of different types of cancer in the last few years.^567–570^

Gold NPs are well known for their plasmonic properties, so they can transform NIR radiation into thermal energy, which can be employed for triggering the release of the payload.^571–573^ Additionally, this two-photon approach has been applied to many MSNs for the delivery of reactive oxygen species, genes and different drugs for the potential treatment of cancer.^472,574–579^

##### Ultrasound

Ultrasound (US) constitutes a very interesting external stimulus combined with MSNs because of the potential spatiotemporal control of the cargo delivery at the site of the disease without producing any damage to healthy tissues.^203,580–583^ Additionally, ultrasound presents some advantages, such as the lack of invasiveness, the availability of ultrasound devices in many clinics, the absence of ionizing radiation, and the relatively easy regulation of tissue penetration.^584^ Thus, US-sensitive MSNs were developed closing the pore entrances with a polymer presenting US-sensitive bonds, that triggered the payload release upon irradiation with US.^203^ US has also been employed together with pH to develop a smart nanocarrier based on MSNs coated with polydopamine.^582^ US irradiation provoked an ultrasonic cavitation effect that triggered the payload release from the cargo. The same cavitation approach was carried out coating the surface of MSNs with folic acid functionalized β-cyclodextrin, which was evaluated both *in vitro* and *in vivo*.^583^ Sodium alginate was also employed for coating MSNs responsive to US with very promising results.^585^ In a different approach, MSNs were functionalized with folic acid and encapsulated in a microbubble, and the antitumor efficacy was corroborated in tumour bearing mice.^581^

##### Temperature

The development of temperature sensitive MSNs for potential cancer treatment is based on the fact that tumour tissues with overexpressed inflammatory markers might show a significant temperature variation up to 5 °C in comparison with healthy tissues. As it has been described above, it is also possible to increase the temperature of the nanocarriers by magnetically induced hyperthermia or by plasmonic effects. Therefore, coating the surface of MSNs with thermo-sensitive polymers, such as poly-*N*-isopropylacrylamide and its derivates, might allow to trigger the payload release only in high temperature situations mentioned above (tumour tissues or when triggered with external stimuli).^586–590^ In similar approaches, different temperature sensitive moieties acting as gate keepers, such as polymers, peptides or nucleic acids, have also been employed to close the pore entrances of MSNs and open them only in response to an increase of temperature.^591–596^

### 4.2. Bone diseases

Bone diseases and associated disorders have considerably grown in recent decades due to the increase in life expectancy.^597–599^ The aging of the population is leading to an increase in bone fractures or defects and therefore the use of treatments to prevent or fix this situation. Most treatments focus on improving bone remodelling with surgery to implant a bone substitute or a bone graft, either natural or synthetic. However, these approaches have limitations such as biomechanics, immunogenicity, availability of a donor, *etc.* As is well known, the gold standard in this context is the use of autograft bone in the damaged area, but this treatment has some limitations, too.^600^ In this regard, tissue engineering has emerged as an alternative in various fields for decades, including the improvement of bone repair and the treatment of various associated diseases, such as osteoporosis, fractures or infections associated with implant surgery.^173,600,601^

There are different biomaterials that have been used to improve bone regeneration as an alternative approach^602,603^ to solve the limitations of the current treatments. These materials, mainly scaffolds, have been enriched with molecules, substances or mesenchymal stem cells that have improved their mechanical, osteogenic and angiogenic properties, in order to be similar to the bone to be replaced.^600^ In addition, the treatment of diseases such as osteoporosis or osteosarcoma, even if effective in reducing the risk of fracture or increasing the survival rate, respectively, also has certain limitations such as bioavailability, tissue specificity, long-term effect or development of resistance.^597,598,600,604,605^

Recently, nanotechnologies have emerged as an ideal alternative, due to their unique properties previously mentioned.^606^ Different types of NPs, both inorganic and organic, are being used as vehicles for different osteogenic and angiogenic molecules to improve bone repair or treat osteoporosis. Within the inorganic NPs we can find silicon NPs (MSNs),^607^ hydroxyapatite NPs,^608^ gold NPs,^609^ magnetic NPs,^610^ and platinum NPs,^606^ among others.^606^ These systems allow us to control the size and functionalization, and they can be used as vehicles of treatment or as contrast agents for diagnosis. Hydroxyapatite NPs have shown to be effective to induce bone tissue formation and serve as vehicles of osteogenic molecules. Gold NPs improved osteoblast differentiation and avoided osteoclast activity. Magnetic NPs are commonly used as vehicles for drug delivery and contrast agents for diagnosis. Platinum NPs have shown antioxidant and anti-osteoclastic properties.^606^ On the other hand, organic NPs have been used as drug delivery functionalized platforms of synthetic polymers. Among them, chitosan NPs (CS NPs),^611^ poly(lactic-*co*-glycolic acid) (PLGA NPs)^612^ and solid lipids NPs^613^ are the most common organic NPs. CS NPs have shown to be effective for antioxidant treatment of age-related disorders and as an excellent nanosystem to load and release different proteins, a function shared with PLGA NPs. Solid lipids NPs increased availability in biological systems and the solubility.^606^

As we previously detailed, MSNs have shown to be biocompatible and biodegradable with excellent properties as drug delivery systems due their size, shape, surface chemistry and surface charge.^147,172^ Furthermore, it is possible to functionalize the surface of MSNs with bone targeting molecules,^614^ such as small bisphosphonates,^615^ tetracyclines, peptides, and proteins, among others.^147,172^ In this section, the osteogenic effects of MSNs as vehicles of different molecules or biological signals on bone metabolism^147,172,607^ will be discussed (Table 3). In addition, the effects of MSNs on bone generation, fracture treatment and wound healing have been detailed, as well as in the treatment of several diseases as osteoporosis. Moreover, stimuli-responsive MSN systems and the synergistic effect of the combination of MSNs with other biomaterial platforms, such as hydrogels or scaffolds, have been explained.

**Table 3:** Examples of different types of silica-based nanocarriers (MSNs) applied in bone diseases

Carrier type(s)	Drug(s)	Cell line(s)	*In vivo* model	Application	Ref.
MSNs	Dexamethasone	BMSCs	Bone targeting	Bone tissue regeneration	616
			Mice	Osteoporosis	
MSNs	BMP-2	MSCs	None	Bone tissue regeneration	617
	pDNA				
MSNs	BFP-BMP-7	MG-63	None	Bone tissue engineering	618
		hMSCs			
MSNs	BMP-2	BMSCs	Ectopic bone formation	Bone tissue engineering	619
	Dexamethasone		Rats		
MSNs	QK peptide	HUVEC	Critical-sized calvarial defect	Bone tissue engineering	620
	Dexamethasone	BMSCs	Rats		
MSNs	Enoxacin	*S. aureus*	Infection model	Bone tissue engineering and infection	621
		BMMs	Rats		
MSNs	miR-26a	BMSCs	None	Bone tissue regeneration	622
MSNs	(rno)-miRNA-26a-5p	BMSCs	None	Bone tissue engineering	623
MSNs	Cu^2+^	Macrophages	None	Bone tissue engineering	624
		BMSCs			
MSN-G3	Levofloxacin	MC3T3-E1	None	Bone tissue engineering and infection	625
	Zn^2+^	*E. coli*			
MSNs	Ag^+^	BMSCs	None	Bone tissue engineering and infection	626
	PDGF-BB	*E. coli*			
		*S. aureus*			
		*P. aeruginosa*			
		*C*. *sporogenes*			
		*B*. *fragilis*			
MSNs	Lactoferrin	ADSCs	None	Bone tissue regeneration	627
				Osteoporosis	
Ce@MSNs		MC3T3-E1	None	Osteoporosis	627
		RAW264.7			
MSNs	Licorice-ISL	BMMs	Calvarial bone erosion	Osteoporosis	628
			Mice		
MSNs	17β-E2	MC3T3-E1	OVX	Osteoporosis	629
			Mice		
MSNs (TDNs)	MCP-1	BMMSCs	OVX	Osteoporosis	630
			Mice		
MSNs	SOST SiRNA	MEFs	OVX	Osteoporosis	615 and 631
	Osteostatin		Mice		
MSNs	DOX	MG-63	None	Osteosarcoma	632
Fe-MSNs magnetic responsive	PLK1 siRNA	KHOS	None	Osteosarcoma	633
MNC-MSNs pH-responsive	PLK1 SiRNA	KHOS	None	Osteosarcoma	634
MSNs ultrasound responsive	DOX	HOS	None	Osteosarcoma	635
MSNs pH-responsive	DOX	HOS	None	Osteosarcoma	291
MSNs pH Responsive	BMP-2	BMSCs	Ectopic bone formation	Bone tissue regeneration	636
	Dexamethasone		Mice		
MSN enzyme responsive	Ag^+^	*S. aureus*	Femur defect	Bone tissue regeneration and infection	637
			Rats		
MSN-PEM/Ti	17β-E2	OBs	None	Bone tissue engineering	638
MSN/nanofibrous scaffold	BMP-2	C2C12	None	Bone tissue engineering	639
	Deferoxamine	hMSCs			
MSN/GelMA hydrogel	Metformin	SHEDs	None	Bone tissue regeneration	640
MSN/HA	Zoledronic acid	L-929	None	Bone tissue regeneration	641
		BMMs			
MSN/PLLA/PCL scaffolds	Dexamethasone	BMSCs	Calvarial bone defect	Bone tissue regeneration	642
			Rats		
nHA/MSNs	Dexamethasone	MC3T3-E1	Calvarial bone defect	Bone tissue engineering	643
			Rats		
MSN/PLGA-PNIPAM hydrogel	miRNA-222	BMSCs	Mandibular defect	Bone tissue engineering	644
	Aspirin		Rats		
MSN/PCL nanofiber scaffold	COL1A1 SiRNA	HDFs	Subcutaneous implantation	Bone tissue regeneration	645
			Rats		

#### 4.2.1. Osteogenic release systems in bone tissue regeneration

There are several molecules or growth factors capable of stimulating and promoting osteogenesis necessary in the processes of bone loss or other alterations related to bone regeneration or wound healing. Among them, the most studied molecules for generating bone directly through osteoblasts are the bone morphogenetic proteins (BMPs),^646^ parathyroid hormone (PTH),^647^ parathyroid hormone related protein (PTHrP),^647^ osteostatin^647–651^ or alternative novel natural peptides currently under study. In addition, bisphosphonates can also be found as main factors acting on osteoclastogenesis.^652,653^ In recent years, the use of SiRNAs or miRNAs as specific molecules has also begun in the treatment of cancer and to a lesser extent in the treatment of bone diseases.^147,172^ Moreover, combinations of these molecules with different biomaterials have been used to improve the conventional treatments,^646,654,655^ avoiding limitations and side effects. In this section, MSNs that have been loaded with these types of molecules to carry out an osteogenic effect in different *in vitro* and *in vivo* models are discussed.

##### Growth factors and biological signals

As previously mentioned, MSNs are being used as potent and intelligent drug delivery systems for several disorders, including osteoporosis and/or in the enhancement of bone regeneration.^173,606,607,646,656^ Among the different options, many authors have opted to study the osteogenic and angiogenic effects of MSNs loaded with factors such as dexamethasone (Dex), bisphosphonates, BMPs, miRNAs and SiRNAs, or a combination of several of them. Dex is a corticosteroid that induces osteogenesis in bone mesenchymal stem cells (BMSCs),^657^ through osteoblast differentiation and the increase of gene expression of different osteogenic factors involved in this process, such as osteocalcin, osteoprotegerin, RUNX family transcription factor 2. The most important problem associated with its use is that at high concentrations Dex decreases the BMSC proliferation, so it is critical to be able to control and maintain the release of this drug thanks to the MSNs, thus avoiding side effects. On the other hand, bisphosphonates are synthetic compounds analogous to pyrophosphate, consisting of a P–C–P skeleton plus two variable carbon residues.^653^ Nowadays they are the most used agents to prevent and treat osteoporosis together with PTH. The bisphosphonates have an avidity for hydroxyapatite and inhibit the dissolution of its crystals as the basis of their antiresorptive properties.^653^ However, these molecules also present certain limitations and side effects that require the use of a nanocarrier such as MSNs.

One of the first studies using alendronate (a bisphosphonate) loaded in mesoporous silica materials for the treatment of osteoporosis or bone repair was carried out by María Vallet-Regí's group in 2006.^658^ In that study, the introduction of phosphate groups enhanced drug loading, inducing apatite formation and opening a promising bone related action field. Other studies have been carried out years later with different antiresorptive molecules loaded on MSNs such as zolendronic acid or ipriflavone, demonstrating the osteogenic and antiosteoclastogenic activity of these nanosystems.^174^ In addition, MSNs are capable of loading hydrophobic compounds such as poorly soluble anabolic agents to improve bone repair. An example of this approach was proposed by Ren *et al.*^616^ with a Dex-loaded nanosystem to treat osteoporosis due to the ability to load and release Dex in a sustained manner to induce osteogenic effects in BMSCs. In addition, the authors coated alendronate, a bisphosphonate with high affinity for the hydroxyapatite presents in bone, to MSN surface as bone targeting ligand to Dex release in bone. The *in vitro* and *in vivo* results demonstrated the biocompatibility of the system, releasing the drug at the concentrations necessary to increase alkaline phosphatase (ALP) activity and matrix mineralization, improving the specific accumulation at bone. Others of the most widely used bone factors in bone regeneration studies are BMPs. These peptides are a family of approximately 30 proteins that are involved in many processes in the body, including stem cell and tissue regeneration.^659^ Their best-known function is to induce an increase in bone formation, and they are currently used in their recombinant form as a treatment for kidney and bone diseases.^659^ Specifically, BMP-2 and BMP-7 have been shown to improve bone repair in human fractures and in different animal models.^659^ BMP-2 was approved by the FDA as molecular treatment in fracture regeneration and spine non unions.^659^ In addition, BMPs have some limitations: they are pleiotropic and activate different tissues, are unable to induce adipogenesis at high concentrations, or are difficult to immobilize due to their higher molecular weight.^659^ In order to avoid some of these limitations, Kim *et al.*^617^ and Luo *et al.*^618^ designed MSN nanocarriers to transfer BMP-2 plasmid DNA into rat mesenchymal stem cells or bone-forming peptide derived FP from BMP-7 in human mesenchymal stem cells and osteosarcoma MG-63 cells, respectively. The nanosystems were successfully internalized by these cells with controlled release of each molecule. In the first study, MSNs induced BMP-2 expression together with other bone related factors protein expression in transfected cells. In the second study, the nanodevice increased osteogenic differentiation *via* ALP activity and nodule matrix mineralization formation. The results of both studies demonstrated that it is possible to avoid the limitations of BMP treatments by loading these molecules into a nanovector such as MSNs.

Another option to explore is to load several molecules involved in bone formation into the same nanosystem to enhance its osteogenic properties. Consequently, Zhou *et al.*^619^ designed a biomaterial based on MSNs loaded with two potent osteogenic molecules, BMP-2 and Dex. In order to avoid the negative effects of high doses of BMPs, the authors used only residues 73–92 of the BMP-2 protein. *In vitro*, the complete nanocarrier showed an excellent biocompatibility and elevated rates of cell internalization in bone mesenchymal stem cells derived from rats. In addition, the presence and release of Dex y BMP-2 from this nanocarrier increased ALP activity, matrix mineralization and the expression of several osteoblastic factors involved in osteoblastic differentiation. The proposed nanocarrier was intramuscularly implanted into rats in an ectopic bone formation model displaying osteogenic actions and accelerating bone repair *in vivo* (microCT and histological analysis). These results indicate that combining several osteogenic factors in a single nanovector such as MSNs can be of great utility in the field of bone regeneration.

Furthermore, the importance of angiogenesis in bone formation^660^ is well known, which is crucial to develop a correct repair of bone tissue in the fracture processes or in other bone diseases. In many fractures or large bone defects, bone repair cannot be completed or fails due to lack of blood vessels at the focus.^661^ To address this problem, Sun *et al.*^620^ evaluated a nanocarrier based on MSNs with osteogenic and angiogenic properties due to the presence of Dex and QK peptide. This peptide, which mimics the helical structure of angiogenic vascular endothelial growth factor, was taken up by MSNs thanks to chitosans. On the one hand, MSNs loaded with the angiogenic peptide QK (QK@chi-MSNs) enhanced the formation of the tubular structure of human umbilical vein endothelial cells *in vitro*. On the other hand, the presence of Dex in the nanosystem (DEX@chi-MSNs) improved ALP activity, nodule mineralization and the expression of genes and proteins involved in osteoblastic differentiation of BMSCs. The efficacy in improving osteogenesis and angiogenesis of the nanosystem loaded with the two molecules (QK/DEX@chi-MSNs) was analysed *in vivo* in a rat model of critical size cranial defect. The results evaluated by imaging and histological techniques indicated that the presence and release of both factors increased bone regeneration through their effects on osteogenesis and angiogenesis, indicating the great opportunities that this system can display in the clinical treatment of bone diseases.

**Fig. 17:**
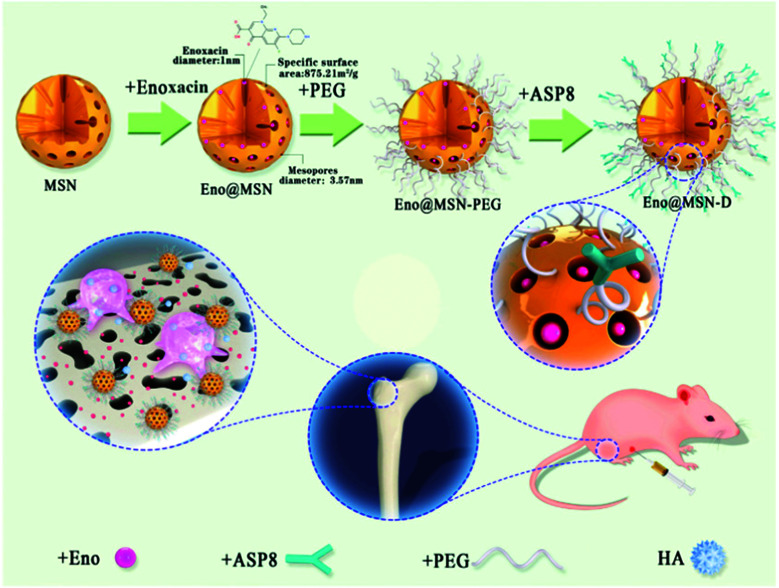
Schematic illustration and mechanism of action of a nanosystem based on MSNs proposed by Yao *et al.*,^621^ with targeting to bone (d-Asp8) and loaded with enoxacin (Eno@MSN-D). Reproduced with permission from ref. 621. Copyright 2021, Frontiers Media S.

Recently there is great interest in combining these elements with other osteogenic or antibiotic molecules to fight against bone infection associated with fractures or implants in bone tissue engineering.^173^ In implant-related infection processes there is a loss of bone mass associated with osteoclastic activity. In this sense, Yao *et al.*^621^ proposed a nanosystem based on MSNs with bone targeting ability thanks to eight repetitive aspartate sequences (d-Asp8) and loaded with enoxacin (Fig. 17). The latter peptide has antibacterial and inhibitory actions on osteoclastic activity, but it has no affinity for bone tissue, so its clinical application is limited. To circumvent this, d-Asp8 has been used as a targeting element to bone due to its affinity for hydroxyapatite to functionalize MSNs (Eno@MSN-D). This nanocarrier decreased both *Staphylococcus aureus* bacterial viability as well as osteoclasticity *in vitro* and specifically released the peptide into infected bone tissue *in vivo* performing the same effects as *in vitro*. In animals treated with the nanosystem, bone infection was reduced, and the loss of bone mass caused by the infection was reversed. This nanosystem could be of great interest in clinical practice as it specifically targets bone, preventing infection and bone loss.

On the other hand, therapy based on RNA has gained importance in the bone regeneration field in recent years as effective and non-toxic treatment in several disorders,^147,172^ especially in cancer. However, SiRNAs or miRNAs are inefficiently internalized by target cells as they are negatively charged and are rapidly degraded. miRNAs are small RNAs that regulate gene expression through post-transcriptional processing that can be loaded into MSNs to induce miRNA stability, effective internalization and osteogenic functions in different bone models. For example, MiR-26a induces bone formation through the regulation of several osteogenic differentiation pathways.^622,623^ Yan *et al.*^622^ and Hosseinpour *et al.*^623^ proposed a nanocarrier based on MSN coated with poly(ethylenimine) (PEI) to load this miRNA effectively. The results indicated that both nanosystems protected the miRNA from degradation, being efficiently internalized in rat bone marrow mesenchymal stem cells and inducing the proliferation and differentiation of these mesenchymal cells to osteoblastic cells through the increase of osteogenic proteins and gene levels implicated in bone regeneration. In addition, in the second study, MSN nanosystem loaded with miRNA-26a was lyophilized, freeze-dried, and evaluated after 3 and 6 months of storage, showing similar results improving osteogenic differentiation and excellent properties to clinical applications.

##### Bone metabolism

It has been known for several decades that silicon is a potent osteogenic element that increased mineralization in bone tissues.^662^ In addition to silicon, phosphorus or calcium, a wide variety of trace elements have been shown to regulate bone metabolism and induce osteogenic effects. These elements have been introduced in different biomaterials,^654^ including MSNs, to enhance their angiogenic and osteogenic effect on bone regeneration. These include zinc, strontium, iron, silver, magnesium, and copper, which have demonstrated *in vitro* and *in vivo* their positive effects in the field of bone diseases.^654^

Shi *et al.*^624^ tested a nanosystem based on MSN nanospheres loaded with copper ions in macrophages and bone mesenchymal stem cells (BMSCs) *in vitro*. Copper has been shown to be a potent osteogenic and hypoxia-inducing agent,^624^ This nanocarrier successfully incorporated and released both copper and silicon ions in a sustained manner. On the one hand, the nanosystem was internalized by macrophages, inducing the expression of osteogenic factors and proinflammatory cytokines and decreasing the expression of factors involved in osteoclastogenesis. On the other hand, the nanosystem was able to stimulate osteoblastic differentiation of BMSCs. Overall, this copper and silicon ion nanovector provided promising results for simple tissue engineering applications.

**Fig. 18:**
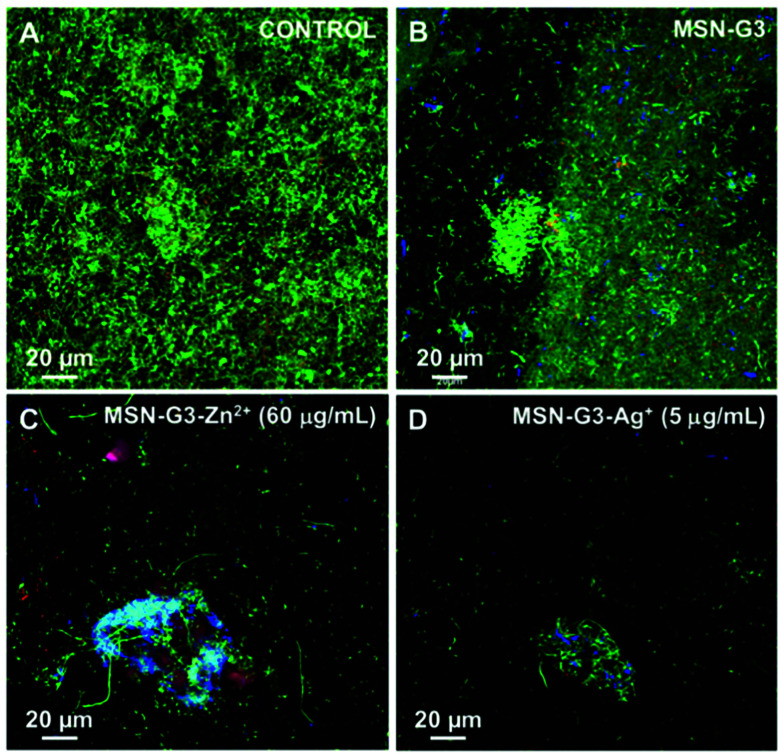
Confocal microscopy images of the antibacterial activity of MSN-G3 nanocarriers loaded or not (B) with Zn^2+^ (C) or Ag^+^ (D) compared to the control (A) onto Gram-negative *E. coli* biofilm formed onto covered glass-disk. Live bacteria are stained in green, dead bacteria in red and the matrix biofilm in blue.^625^ Reproduced with permission from ref. 625. Copyright 2021, Elsevier Ltd on behalf of Acta Materialia Inc.

On the other hand, inspired by the idea of combining osteogenic or antibiotic molecules to fight against bone infection and the associated bone loss,^173^ Vallet-Regí's Group^625^ proposed a versatile dual nanocarrier to fight bone infection and to improve bone regeneration. For this purpose, levofloxacin, a potent antibiotic widely used in clinical practice, and Zn^2+^ ions, which induce osteogenesis and exert antibacterial effects in different *in vitro* and *in vivo* models, were loaded into MSN nanosystems (Fig. 18). To improve the bacterial internalization and the Zn^2+^ loading capacity, the proposed nanocarrier was functionalized with a polyamine dendrimer G3 on the MSN surface. In biofilm and planktonic *Escherichia coli* bacterial culture, the complete nanosystem with Zn^2+^ and levofloxacin induced an antibacterial efficiency of almost 100%. The system was not only successful in completely reducing bacterial infection but also increased osteoblastic differentiation into preosteoblastic cells without affecting their viability. This novel nanosystem can combine different antibiotics, drugs and ions to be chosen according to the clinical needs of each patient, being of particular interest to fight infection and diminished bone regeneration *via* local administration. Following the same objective, Ma *et al.*^626^ developed a nanosystem based on MSNs encapsulated with silver nanocrystals and loaded in their pores with platelet-derived growth factor BB (PDGF-BB). On the one hand, Si released by the nanocarrier enhanced osteoblastic differentiation of BMSCs by increasing bone factor proteins and ALP activity. On the other hand, Ag^+^ was released slowly over time, maintaining a sustained antibacterial effect against different bacterial strains. Moreover, MSNs loaded with PDGF-BB increased angiogenesis in BMSCs, due to the increased secretion and protein expression of different angiogenic markers such as vascular endothelial growth factor or HIF-1alfa. In summary, the proposed nanosystem not only overcame infection and promoted osteoblastic differentiation and mineralization but also enhanced angiogenesis, thus becoming a powerful tool in tissue engineering.

#### 4.2.2. Osteoporosis

With the increase in life expectancy worldwide, there has been a considerable increase in bone-related diseases such as osteoporosis.^597^ The term osteoporosis indicates loss of bone mass and bone quality (*e.g.*, increased porosity), with the consequent risk of fractures of the spine, wrist, hip or ribs.^663^ According to the World Health Organization (WHO) definition, osteoporosis is considered to be a “progressive systemic skeletal disease characterized by low bone mass and microarchitectural deterioration of bone tissue, with consequent increased bone fragility and susceptibility to fractures”. This pathology is a “silent epidemic”, as there are often no specific symptoms before the fracture occurs. Approximately more than 200 million people in the world currently suffer from this disease;^597^ a population that is expected to grow over the next few years due to longevity and lifestyle patterns.

Osteoporosis mainly affects postmenopausal women (with estrogen depletion), but also men from the age of 50 years.^599^ The osteoporotic fractures are common and are associated with physical, social, and economic factors. The number of hip fractures worldwide was about 2 million in 1990, with the estimate for the year 2050 being 6 million.^598^ This type of fracture requires hospitalization, they are lethal in 20% of the cases, and result in permanent disability in about half of the patients. This has led to a health care cost of 3.5 billion euros in the European Union per year.

In osteoporosis there is an alteration in bone remodelling due to two fundamental causes, normally associated with (1) an increase in bone remodelling multicellular units and (2) a predominance of resorption over bone formation.^652^ The origin of bone mass loss is multifactorial; thus, it is associated with age and menopause (primary osteoporosis), prolonged treatment with certain drugs or other risk factors, among which are some endogenous syndromes.^652^ Conventional treatments for osteoporosis are currently divided into two groups: on the one hand, antiresorptive drugs that act directly on osteoclasts, limiting their activity and thus increasing bone mass and bone strength;^653^ and on the other hand, anabolic agents that act on osteoblasts, increasing bone formation and reversing the loss of bone mass.^604^ However, although these currently used pharmacological therapies are effective in decreasing the fracture risk and improving bone mineral density, they have some limitations related to bioavailability and toxicity issues in non-skeletal tissues, and cannot offer long-term solutions.

Among the antiresorptive drugs, the most widely used have been and are the bisphosphonates, which block the activity of the osteoclasts by inhibiting farnesyl pyrophosphate synthase, an enzyme involved in the membrane that prevents them from adhering to the bone.^653^ In this sense, they decrease bone resorption through an increase in osteoclast apoptosis. Although they are successful in preventing and reducing the risk of bone fracture, the direct effect on the increase in bone mass is relatively small (<2% per year). On the other hand, bisphosphonates present bioavailability problems and are not easily absorbed by the gastrointestinal tract.^653^ Therefore, high doses are necessary, which leads to intestinal complications. Bisphosphonates must be administered with a complex schedule of times and have problems associated with the administration of other drugs. In addition, they have a series of side effects when they are administered orally, such as mandibular necrosis due to excess inhibition of bone resorption or esophagitis due to the direct action of these drugs on the esophageal mucosa.^653^

Examples of antiresorptive drugs currently in use include Raloxifene and Denosumab.^653^ The former is a second-generation non-steroidal drug, a selective estrogen receptor modulator (SERM) that decreases bone loss by inhibiting cytokines associated with increased bone resorption. But it has the same limitation: the achievable bioavailability is very low. The second is an anti-receptor activator of nuclear factor κB ligand (RANKL) monoclonal antibody,^653^ which acts by decreasing osteoclast activation and differentiation, but since it can also be expressed in T lymphocytes, its inhibition could lead to impaired immunity. Among the anabolic drugs, parathyroid hormone (PTH) was the first anabolic agent to be approved. Since its approval by the FDA in the United States in 2002 and in Europe in 2004, recombinant human PTH (1–34) (teriparatide) is one of the bone anabolic agents currently available for clinical use.^647,664^ This hormone improves osteoblast function by binding to a specific receptor (PTHR1) and activating several signalling pathways related to bone formation, as the Wnt/B-catenin pathway. It has been shown to decrease the risk of fracture by up to 65% in osteoporotic patients when administered intermittently (one injection per day for 18 months), considerably increasing the bone mass at both the vertebral and appendicular levels.^647,664^ However, the PTH treatment presents some limitations, such as the activation of bone resorption and the partial efficiency on nonvertebral fractures.^647,664^ In this sense, abaloparatide was recently approved as a PTH (1–34) related peptide (PTHrP) analog used for the treatment of postmenopausal women osteoporosis with high risk of fracture or treatment failure with other osteoporotic medications.^647^ Intermittent day injection of this analog peptide has an anabolic effect on bone analogous to teriparatide. This peptide activates signal transduction with equal potency as PTH, but produces less activation of bone resorption. Previously, PTHrP through its N-terminal fragment had already been shown to exert osteogenic properties similar to PTH in different *in vitro* and *in vivo* models. On the other hand, its C-terminal fragment performed anti-osteoclastic and osteogenic actions in models of osteoporosis associated with diabetes and glucocorticoid treatment.

Having exposed the limitations and drawbacks of most osteoporosis treatments, it seems logical that alternative treatments have been sought to overcome these problems. In this regard, nanotechnology has emerged as an innovative and alternative treatment. The fundamental objective of this alternative approach is to be able to directly target the drug to the bone, in order to avoid possible side effects and to ensure a long-term effect, minimizing systemic toxicity and increase the pharmacokinetic outline and efficiency of chemical drugs. In the field of osteoporosis treatment, different NPs have been developed, such as dendrimers, liposomes, polymeric NPs, iron oxide or gold NPs, and MSNs. As highlighted throughout this review, MSNs exhibit higher robustness due to their silica structure, and they are mechanically, thermally, and chemically stable. Thus, different molecules involved in bone formation and resorption loaded in MSNs are currently being studied as experimental alternatives. These nanocarriers can load different drugs, carrying them to the target cell or tissue.

Recently, different groups have developed new nanosystems based on MSNs loaded with novel natural molecules that directly affect osteoblast- and osteoclast-mediated bone formation or resorption.^607,646,654^ Noh *et al.*^627^ developed a nanocarrier based on MSNs loaded with an osteogenic growth factor called lactoferrin with potential application in the treatment of bone fractures or diseases such as osteoporosis. Lactoferrin is a molecule present in colostrum with anti-inflammatory properties that improves macrophage activity and immunity through interaction with several immune cells. In addition, this molecule has shown osteogenic properties *in vitro* and *in vivo*, inducing new bone formation especially when loaded in a biomaterial because of its short half-life in the blood. The nanosystem containing lactoferrin was functionalized with tannic acid, as it can interact with different biomacromolecules through hydrogen bonding and electrostatic interactions. The proposed nanocarrier induced prolonged release of lactoferrin (28 days) and osteo-differentiation of adipose-derived stem cells, increasing alkaline phosphatase activity, calcium accumulation and osteocalcin and osteopontin gene expression (osteogenic markers) in these cells.

In order to eliminate the negative effects of osteoporosis treatments affecting osteoclast related to oxidative stress, Pinna *et al.*^665^ proposed a ceria MSN nanocarrier (Ce@MSNs), which can shuttle between Ce_2_O_3_ and CeO_2_. This nanosystem acts as an antioxidant reactive oxygen species scavenger, a key factor in bone cells apoptosis, with osteogenic properties *in vitro* in a concentration-dependent manner. The authors tested the osteogenic effects of this nanocarrier in normal and stressed situations in MC3T3-E1 mice cells. First, Ce@MSNs were successful internalized by the pre-osteoblastic cells, without affecting cell proliferation, inducing a significant decrease in oxidative stress induced by *t*-butyl hydroperoxide. Therefore, an increased in matrix mineralization was observed in MC3T3-E1 cell cultures without osteogenic supplements. Moreover, the authors developed a co-culture with MC3T3-E1 cells and RAW264.7 macrophages (osteoclast precursors), demonstrating the osteogenic, antioxidant and anti-osteoclastogenic effects of the Ce@MSN nanocarriers. The possibility of combining these three effects in the same biomaterial makes this nanosystem a unique system in the treatment of bone diseases or tissue engineering approaches in bone regeneration situations. In the same sense of avoiding the side effects of osteoporosis treatment in relation to osteoclastogenesis, Sun *et al.*^628^ studied a nanocarrier based on MSN loaded with a natural molecule against bone resorption and inflammatory bone destruction named licorice-derived bioactive flavonoid isoliquiritigenin (ISL). The authors examined this promising nanosystem (MSNs-ISL) to avoid osteoclast-mediated bone loss *in vitro* and *in vivo.* An excellent biocompatibility, together with an acid sensitive continuous ISL liberation in primary bone marrow-derived macrophages, was found in the presence of the MSNs-ISL nanocarrier. In these cells, MSNs-IL decreased receptor activator of nuclear factor-κB ligand (RANKL) protein and gene expression levels. In addition, this nanocarrier decreased osteoclast-related factor genes and protein levels, inhibiting osteolytic capacity of osteoclasts and nuclear factor of activated T cell (NFATc1) gene expression. In an *in vivo* mouse model for calvarial bone erosion induced by lipopolysaccharide, this novel nanosystem inhibited inflammation, osteoclast activity and osteoclast related gene levels, including NFATc1 and cathepsin K, according to *in vitro* results. This model constitutes a considerable advance in the fight against bone loss associated with osteoclastic activity and inflammation.

Among the *in vivo* models of osteoporosis, one of the most widely used is the one involving ovariectomy (OVX) in female animals.^666^ These animals develop the disease, resulting in a loss of bone mass density and different alterations in bone architecture, mainly in trabecular bone.^666^ Estrogen insufficiency is one of the most common reasons of osteoporosis in postmenopausal women. Chen *et al.*^629^ proposed a novel 17β-estradiol (E2) loaded MSN nanocarrier in order to avoid the secondary negative effects of hormone therapy as unspecific accumulation in other organs. The authors used MC3T3-E1 preosteoblastic cells and OVX osteoporotic mice to evaluate the osteogenic effect of the nanosystem *in vitro* and *in vivo*, respectively. To improve the efficacy of targeting cells and bone tissue, the surface of the nanosystem was decorated with ethylenediaminetetraacetic acid (EDTA) [E2@MSN-EDTA], and the upconversion core of E2-nanocarrier (carbon shell upconversion NPs) operates as an exceptional imaging agent for following the E2 release. The nanosystem showed an excellent E2 loading and sustained release properties and improved the osteogenic differentiation of preosteoblastic cells. *In vivo*, the complete nanosystem increased bone mineral density values twice as much as the OVX group values, mitigating osteoporosis and decreasing hormone damage in the uterus. In addition, uterine weight and luminal epithelium were also decreased in the presence of the nanocarrier. In conclusion, this E2@MSN-EDTA nanocarrier showed highly promising drug delivery and osteogenic properties *in vitro and in vivo* in osteoporotic mice, avoiding the side effects of hormone therapy. As we previously mentioned, estrogen deficiency induced an inflammatory disorder associated with an increase in proinflammatory cytokine levels mediated by T cell activation. This situation altered the equilibrium between regulatory T cells (Tregs) and Th17 cells and reduced the osteogenic differentiation of bone marrow mesenchymal stem cells (BMMSCs). Taking into consideration the same *in vivo* OVX estrogen depleted osteoporosis model, Yang *et al.*^630^ proposed a T cell-depleting nanocarrier (TDNs) based on MSN that increased the osteogenic BMMSC deficit and reversed the loss of bone density mass. This novel TDN nanosystem was combined with Fas ligand (FasL) as the corona and monocyte chemotactic protein-1 (MCP-1) as the core. The results obtained by these authors indicate fast MCP-1 liberation, thanks to the conjugated FasL present on the TDN surface, allowing the apoptosis of activated T cells *in vitro* and *in vivo*. Thanks to the apoptotic indicators recognized by macrophages, the balance of Treg/Th17 cells was restored, returning the inflammatory situation to normal values, and increasing the osteogenesis of BMMSCs, as well as recovering the non-osteoporotic bone mineral density values induced by the OVX model. The system proposed by the authors is a powerful tool to address the treatment of bone loss associated with estrogenic depletion.

**Fig. 19:**
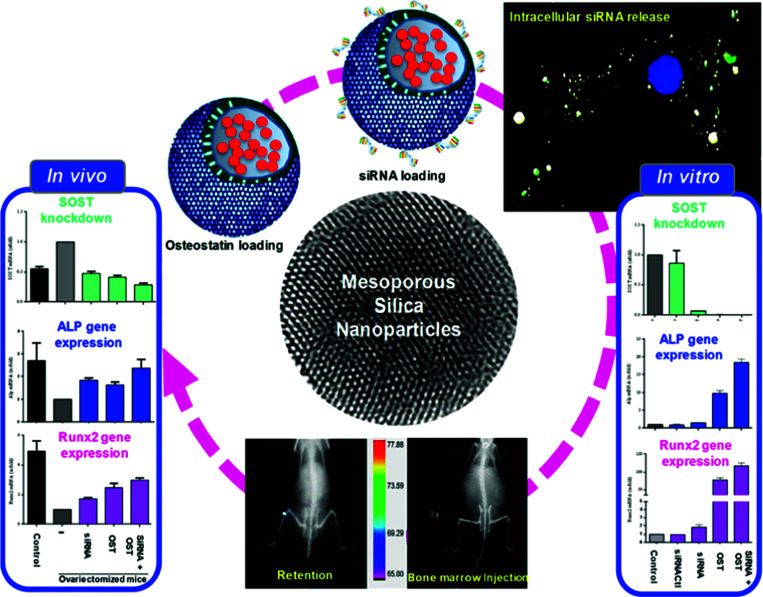
Schematic illustration of the proposed nanocarrier based on MSNs loaded with osteostatin and siRNA to knockdown SOST. Both *in vitro* and *in vivo* results showed an increased in the gene expression of early osteogenic differentiation markers.^631^ Reproduced with permission from ref. 631. Copyright 2019, American Chemical Society.

**Fig. 20:**
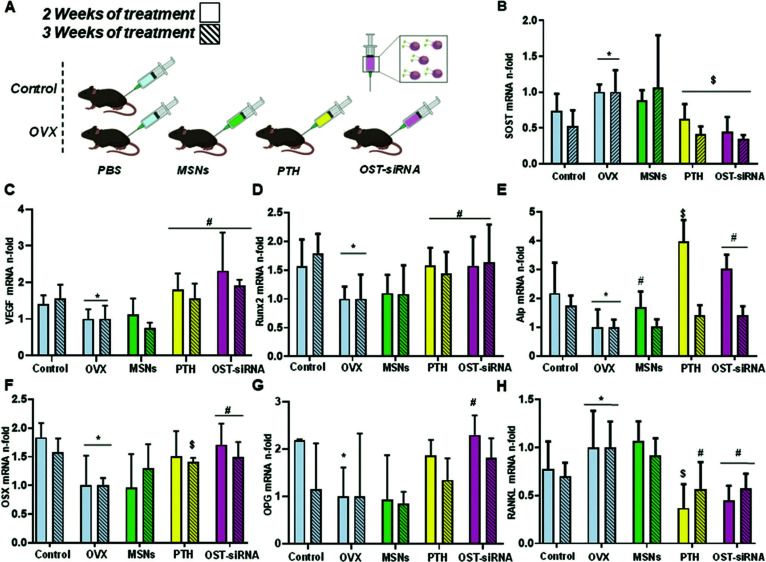
(A) *In vivo* assay performed by Vallet-Regí's Group. (B–H) *In vivo* evaluation of different gene expressions (measured by qPCR) in femur bone of healthy controls and ovariectomized (OVX) mice in the presence of SOST-siRNA and osteostatin loaded in MSNs-PA@PEI (OST-siRNA). A gold standard treatment control has been used (free PTH 100 μg kg^−1^ every 2 days) (PTH), and free MSNs-PA@PEI nanoparticles were used as negative control (MSNs). *p < 0.05 *vs.* control; #*p* < 0.05 *vs.* OVX; $*p* < 0.01 *vs.* OVX.^615^ Reproduced with permission from ref. 615. Copyright 2021, Wiley-VCH Gmb.

**Fig. 21:**
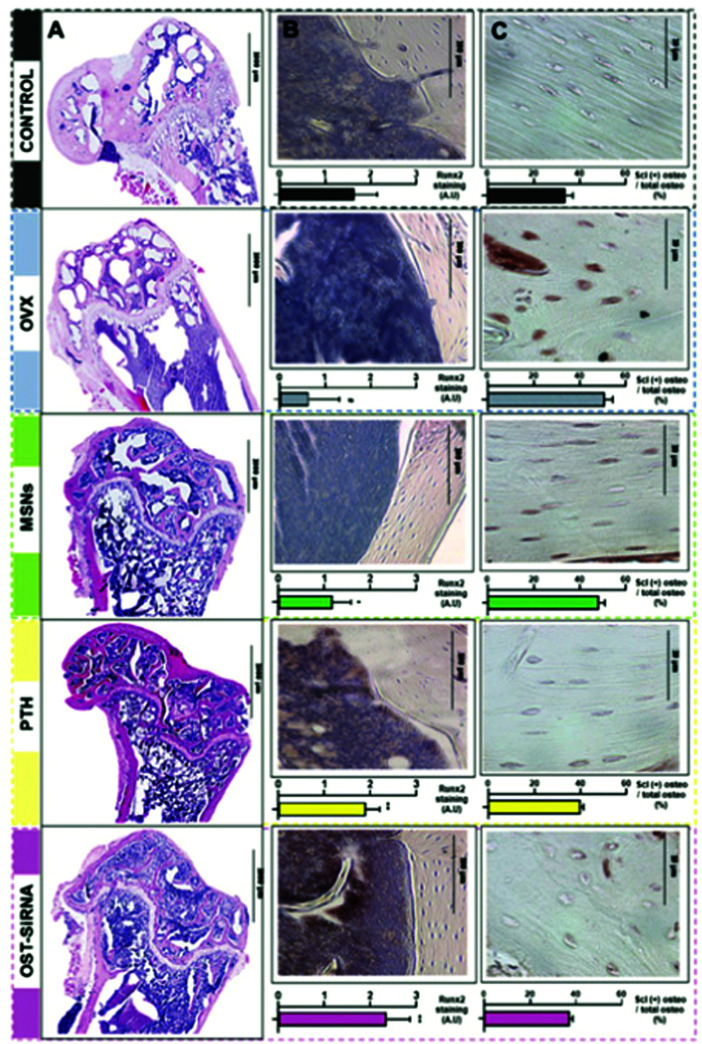
Histological studies and immunostaining for Runx2 and sclerostin in the femur of each group. (A) Representative images of the femur histological sections. (B) Representative Runx2 immunostaining in mice femurs. (C) Total and sclerostin-positive osteocytes in the cortical femur. Positivity (brown stain).^615^ Reproduced with permission from ref. 615. Copyright 2021, Wiley-VCH GmbH.

Of particular interest are the studies by Vallet-Regí's Group, where Mora-Raimundo *et al.*^615,631^ proposed a novel nanocarrier loaded with osteostatin peptide and SiRNA of SOST gene as a promising alternative for osteoporosis systemic treatment (Fig. 19–21). Osteostatin is the active domain of the C-terminal fragment of PTHrP and exerts anti-osteoclastic and osteogenic properties *in vitro*^649,650,667–669^ in mesenchymal and osteoblastic cell lines, it also improves bone repair and osteoporosis *in vivo.*^648,651,670,671^ The SOST gene is an inhibitor of bone formation through the Wnt signalling pathway, diminishing osteoblastic proliferation and differentiation.^615,631^ The understanding of the different signalling pathways involved in bone has made possible the use of new alternatives in the treatment of osteoporosis. The use of treatments associated with small interfering RNA silencers (siRNA) has recently started. However, the main disadvantage of SiRNA, and other peptides, is degradation as they are exposed to different enzymes, resulting in a short half-life in blood. This problem can be resolved using nanocarriers based on MSNs. Particularly, Mora-Raimundo *et al.*^615,631^ studied a nanocarrier coated with PEI in order to successfully release SOST siRNA and osteostatin into mouse embryonic fibroblastic cells and in two OVX osteoporotic mice models, injected in the femoral bone marrow^631^ or administered systemically by subcutaneous injection.^615^*In vitro*, the results showed an effective SOST gene expression inhibition, associated with an increase of gene expression of osteogenic markers. In the first place, the complete nanocarrier was administered in the femoral bone marrow of OVX osteoporotic mice, recovering bone density inducing by OVX through silencing SOST gene expression and increasing the gene expression of osteogenic factors, in agreement with the *in vitro* experiments (Fig. 19). This effect was more evident when the nanocarrier was loaded with osteostatin and SOST siRNA compared to each biomolecule separately. The authors wanted to go a step further, modifying this nanosystem with PEI to obtain a good colloidal stability and with alendronate to develop bone targeting ability, and then injected it subcutaneously in OVX mice. SOST inhibition and increased levels of gene expression of angiogenic and osteogenic markers (Fig. 20) were observed in femur associated with bone formation acceleration, improving the microarchitecture of the bone by immunohistochemical, histological and μCT analyses (Fig. 21). These positive effects on bone parameters resulted in the recovery of density values of the healthy bone mineral, which had been decreased by the ovariectomy. This approach represents a new treatment for osteoporosis with better results than the current clinical anabolic treatment (PTH), reducing the negative side effects.

#### 4.2.3. Osteosarcoma

The efficacy of MSN-based nanosystems has been previously discussed in the cancer treatment section. Regarding bone cancer, osteosarcoma is the most common primary bone tumour (70%), especially in young people with a second peak in patients aged over 50.^672^ This type of cancer consists of a malignant primary solid tumor derived from osteoprogenitor cells and is easily expandable. The first approach for its treatment consists of resection of the affected area, usually accompanied by chemotherapy and filling of the removed area. Although the treatments are efficient and have increased the survival rate substantially, there are several limitations, as resistance or side effects, which can be overcome thanks to MSN based nanosystems. Different types of NPs have been used in the treatment of osteosarcoma.^605^ Among the organic NPs, the most used have been polymers, micelles, liposomes and dendrimers.^605^ The most important inorganic NPs are MSNs, metal NPs, carbon nanomaterials and calcium phosphates.^605^ Nowadays, the most widely used approach is the combination of two or more of these systems to form complex nanocomposites.^673^

In the case of MSNs, there are several examples of MSN-based nanosystems that are very effective in treating this type of disease.^605,673^ Among those studied, we can highlight DOX-loaded MSN-based nanosystems and their relationship with serum proteins,^632^ iron oxide-loaded MSNs coated with PEI to load SiRNAs,^633^ magnetic MSNs to release RNAs with an additional coating with tannic acid for further protection with pH-sensitive properties,^634^ or MSNs that respond to ultrasound.^580^

**Fig. 22:**
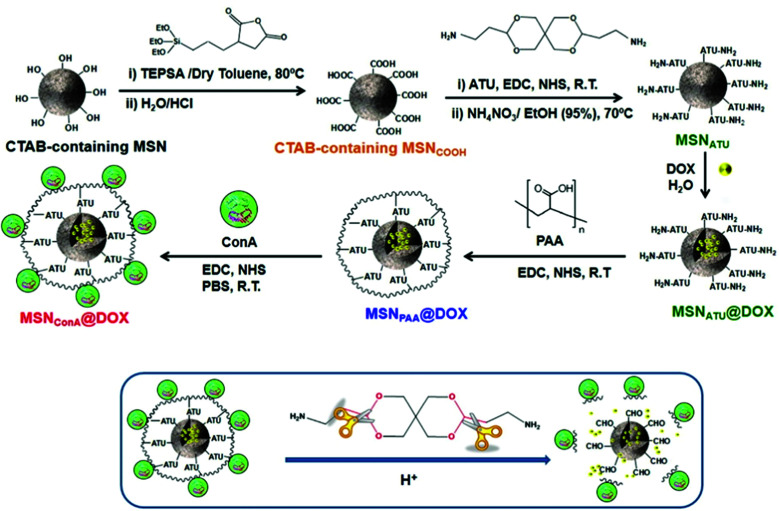
Schematic illustration of the nanocomposite proposed by Vallet-Regí's group loaded with DOX and different functional blocks to treat bone cancer. The nanocarrier was grafted with a pH-cleavable linker (ATU), coated with an acid-degradable polymer (PAA) and covalently linked to lectin ConA.^291^ Reproduced with permission from ref. 291. Copyright 2018, Elsevier Ltd on behalf of Acta Materialia Inc.

Of special interest is the study developed by Vallet-Regí's group^291^ where the authors proposed a novel MSN nanocarrier loaded with DOX as a multifunctional nanodevice for different functional blocks to treat bone cancer (Fig. 22). First, with the aim to reduce early drug release and provide pH-responsive properties, the authors used a polyacrylic acid capping layer linked *via* an acetal that can be cleaved under acidic conditions

Secondly, lectin concanavalin A (ConA) was used as the targeting ligand. ConA is selectively internalized into tumour cells due to its ability to recognize sialic acids (SA) that are overexpressed in these types of cells. The nanocarrier was effectively internalized by SA overexpressed osteosarcoma cells HOS compared to MC3T3-E1 preosteoblastic non-tumoral cells. The internalization of this pH-responsive multifunctional nanocomposite induced a significant decrease in HOS viability with minor DOX cargo (2.5 μg mL^−1^) and without affecting non-tumoral cells. This promising new nanosystem specifically kills osteosarcoma tumor cells with almost no effect on healthy cells, making it a powerful weapon against this type of cancer.

#### 4.2.4. Stimuli-responsive MSNs

As mentioned above, the most effective nanomaterial must be able to protect the drugs needed to treat the different bone diseases or disorders from degradation and transport them to the desired cell or tissue. The critical point is that the drugs or molecules used in that treatment should be released in a controlled manner into the target bone. MSNs are ideal biomaterials to achieve this goal, through various strategies to place different organic or inorganic substances at the pore openings that act as pore blockers or gatekeepers, avoiding the premature release of the cargo.^172,174,402^ Stimulus-response systems can control this release at the target organ in response to internal (pH, redox potential, *etc.*) or external stimuli (magnetic field, chemical species, *etc.*).^172,174,402^

The first aspect to identify is the specific application required for each disease and adapt the nanosystem to it. In some cases, to improve the treatment, it is possible to combine two or more internal or external stimuli. In the case of bone diseases, studies focused on this type of stimulus-response systems are limited compared to their application in cancer or other diseases. There are more studies of these nanosystems in response to internal and external stimuli in the field of bone cancer and osteosarcoma treatment^674^ than in osteoporosis. In this context, these nanosystems have been developed loaded with cytotoxic drugs and/or osteogenic molecules in response to stimuli, such as pH, ultraviolet light, ultrasound, or other stimuli.

In order to achieve a synergistic osteoinductive effect on bone regeneration, Gan *et al.*^636^ fabricated a nanosystem based on chitosan-functionalized MSNs (chi-MSNs) for dual release of Dex and BMP-2, factors that improve osteoblast differentiation. MSNs were coated with chitosan by crosslinking glycidoxypropyltrimethoxysilane to load BMP-2, while Dex was loaded into the mesopores. When the pH decreased, chi-MSNs rapidly released BMP-2. This led to effective internalization by bone marrow mesenchymal cells, followed by the release of Dex into their cytosol. The nanocarrier was able to synergistically increase osteoblast differentiation in these cells and bone regeneration *in vivo*, in a mouse model of ectopic bone formation. This pH-responsive nanosystem demonstrates the possibility of combining two factors in a single model to effectively enhance osteoblastic differentiation in clinical situations where bone formation needs to be restored.

On the other hand, in the context of bone regeneration, bacterial infection associated with biomaterial implantation can lead to failure and the associated need to re-operate the patient, with the corresponding personal hardship and economic cost. To avoid this situation, different research groups are designing nanocarriers capable of regenerating bone and avoiding a possible infection. Ding *et al.*^637^ have proposed a nanosystem that can eliminate an infection and regenerate bone simultaneously. For this purpose, MSNs were loaded with Ag ions in a single step and then polyamine hydrochloride and poly-L-glutamic acid (PG) were assembled using the layer-by-layer assembly (LBL) resulting in LBL@MSN-Ag. *Staphylococcus aureus* secrete glutamyl endonuclease that can degrade PG, an amide-binding polyamide. In a second step, Ti substrates were modified with polidopamine and used as surface to coat LBL@MSN-Ag NPs. LBL@MSN-Ag modified Ti substrates showed enzyme-responsive Ag ion release and induced an antibacterial effect *in vitro* through V8 enzyme over-expressed at the infection site. *In vivo*, the complete nanoplatform successfully treated a femur-defect rat model infected by bacteria, inducing osteogenesis and new bone formation (histology and microCT). As demonstrated by the authors, the proposed nanoplatform is able to combine infection control and maintain or enhance impaired osteogenesis in bone defect models.

#### 4.2.5. MSNs combined with other biomaterials in bone tissue engineering

Another possibility being explored by research groups is to achieve complex platforms with synergistic effects combining osteogenic factors (E2, BMPs, Dex, *etc.*), MSNs and other biomaterials, including scaffolds, hydrogels or hydroxyapatite, among others. A simple approach to control bone homeostasis by osteoclasts and osteoblasts was proposed by Hu *et al.*^638^ using MSN nanocarriers loaded with E2, fabricated using the LbL assembly technique, deposited onto titanium substrates. A chitosan–gelatin paired multilayer was created on this surface. The MSNs were successfully internalized by osteoblasts, releasing the E2 content through degradation of the multilayers. The complete system showed cell compatibility and induced osteoblastic differentiation, increasing ALP activity and matrix mineralization, and displaying osteogenic effects very useful to bone regeneration applications. We previously indicated the importance of angiogenesis in the process of bone regeneration. In this regard, Yao *et al.*^639^ designed a dual-drug delivery MSN nanocarrier combined with a 3D nanofibrous gelatin scaffold for deferoxamine (DFO) and BMP-2 release. An increase in angiogenesis can occur through the activation of hypoxia-inducible factor-1 alpha by DFO hypoxia-mimetic drug. BMP-2 was placed in the pores of MSNs for its sustained release over time and DFO was covalently conjugated through chitosan for a shorter release, extending its half-life and decreasing its cytotoxicity, and both were incorporated in a porous gelatin scaffold. In both human and mouse mesenchymal stem cells, the proposed scaffold/MSN released DFO and BMP-2 at 10 and 28 days, respectively, inducing synergistic angiogenic and osteogenic effects *in vitro.*

As an alternative to BMPs’ limitations on bone repair, Qu *et al.*^640^ proposed to load metformin (MF) in MSN nanospheres combined with gelatin methacryloyl (GelMA) photocrosslinkable hydrogels to tissue engineering applications. MF is typically used for type 2 diabetes treatment, but exerts osteogenic actions in mesenchymal cells, inducing bone repair. The complete MF-MSN/GelMA hydrogel synergistically increased osteoblastic differentiation of human exfoliated deciduous teeth cells, analyzed by ALP activity, matrix mineralization and osteoblastic gene expression. This type of approach can be of great interest and applicability in craniomaxillofacial clinical requests in bone regeneration.

**Fig. 23:**
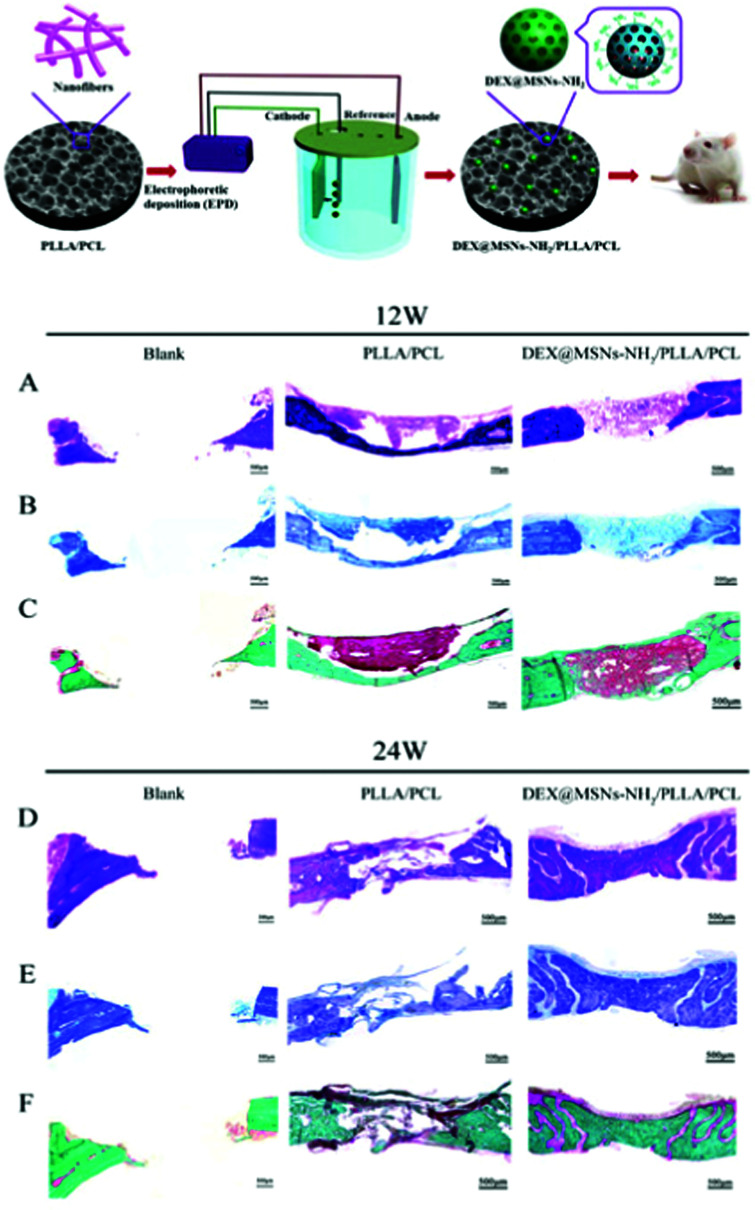
Schematic illustration of the DEX@MSNs-NH2/PLLA/PCL composite scaffold fabrication. H&E staining (A and D), T-blue staining (B and E) and Goldner's staining (C and F) studies of rat calvarial bone defects after 12 and 24 weeks of implantation of PLLA/PCL (blank) and DEX@MSNs-NH2/PLLA/PCL scaffolds.^642^ Adapted from ref 642. Copyright 2016, American Chemical Society.

On the other hand, fewer studies have been performed on MSN-based nanosystems evaluated in osteoclastic cells in bone regeneration. Zhu *et al.*^641^ proposed a MSN/hydroxyapatite (MSNs/HA) platform loaded with the anti-osteoclastic drug zoledronic acid (ZOL) and coated onto a stainless Kirschner wire substrate with the purpose of decreasing osteoclastic resorption activity. MSNs induced a ZOL sustained liberation profile compared to HA alone, inducing a decrease of tartrate-resistant acid phosphatases staining levels, cell proliferation and pit formation of L-929 cells and macrophages derived of bone marrow. As mentioned above, Dex is a potent dose-dependent osteoblastic differentiation factor that has been widely used in bone regeneration studies in different animal models. One of the most broadly performed is the calvarial bone defect. In this model, Qiu *et al.*^642^ and Jia *et al.*^643^ studied two types of MSN based nanoplatforms loaded with this drug. The first study focused on an acid/poly(ε–caprolactone) (PLLA/PCL) nanofibrous scaffold as the template of MSN nanocarrier loaded with Dex added through electrophoretic deposition (Fig. 23). In the second study, the authors designed a novel MSN coated nanohydroxyapatite system (nHA-MSNs). Both biomaterials induced significant osteogenic effects *in vitro* in rat bone marrow-derived mesenchymal stem cells and MC3T3-E1 preosteoblastic cells, respectively. In addition, MSN/PLLA/PCL scaffolds and nHA/MSNs improved the repair of a calvarial defect in rats compared with their respective controls. The designed platforms have demonstrated the possibility to enhance bone regeneration combining different biomaterials and osteogenic or antiosteoclastic molecules.

As we indicated previously, the use of treatments with MSNs loaded with miRNAs or siRNAs is increasing in all *in vivo* preclinical model diseases, including bone disorders. Specifically, miR222 is involved in neural differentiation, increasing the bone mesenchymal cell differentiation and the insufficient innervation that occurs in bone tissue regeneration. Lei *et al.*^644^ fabricated a poly(ethylene glycol)-*b*-poly(lactic-*co*-glycolic acid)-*b*-poly(*N*-isopropylacrylamide) injectable hydrogel coated with thermo-responsive MSNs and loaded with miR222 and aspirin (ASP) (miR222/MSN/ASP hydrogel). ASP is widely used as a non-steroidal anti-inflammatory treatment in several disorders and induces osteogenic effects *in vitro* and *in vivo.* The authors tested the miR222/MSN/ASP hydrogel *in vivo* in a mandibular bone defect in rats exhibiting neurogenesis and improving osteogenic bone repair effects. Aiming to enhance long-term sustained release of SiRNAs from genes involved in bone regeneration, Pinese *et al.*^645^ proposed scaffolds fabricated by electro spinning as support to release PEI-coated MSNs loaded with type I collagen SiRNA by surface adsorption or encapsulation in nanofibers. While the scaffolds demonstrated the ability to release siRNA for up to 30 days, the encapsulated nanosystem achieved sustained release for up to 5 months, inducing more effective silencing of collagen type 1 gene *in vitro* in human dermal fibroblasts. In a bone tissue regeneration model by subcutaneous implantation in rats, the studied scaffold showed an excellent biodistribution of SiRNA (close to the implant) and decreased the fibrous capsule formation. This scaffold platform constitutes an interesting approach to increase the efficiency of MSN nanocarriers for long-term release in bone tissue engineering.

### 4.3. Infection diseases

Infections are increasingly emerging as a cause of morbidity and mortality worldwide, due to the rapid emergence and dissemination of drug-resistant pathogens that have acquired new resistance mechanisms, leading to antimicrobial resistance (AMR), which threaten our ability to treat common infections.^675–677^ AMR occurs when bacteria, viruses, fungi and parasites change over time, when they are exposed to antimicrobial drugs, such as antibiotics, antivirals, antifungals, and anthelmintics, respectively. Consequently, the drugs become ineffective, and infections persist in the body, increasing the risk of spread to others, severe illness and death. In fact, due to the lack of effective antimicrobials for prophylaxis and treatment of infections, relevant medical procedures, such as cancer chemotherapy or diabetes management, as well as major surgery, such as organ transplantations, caesarean delivery, or prosthesis implantation and replacement, have become very risky.

According to the WHO, AMR is a major concern threatening human global health.^678^ Currently, drug-resistant diseases cause at least 700 000 deaths each year, and this figure could grow to 10 million by 2050.^679^ In fact, it is foreseen that by this date, more people will die from AMR than cancer.^680^ Moreover, AMR increases the healthcare cost for the sanitary systems related to long-stay in hospitals and more intensive care required.^681^

**Fig. 24:**
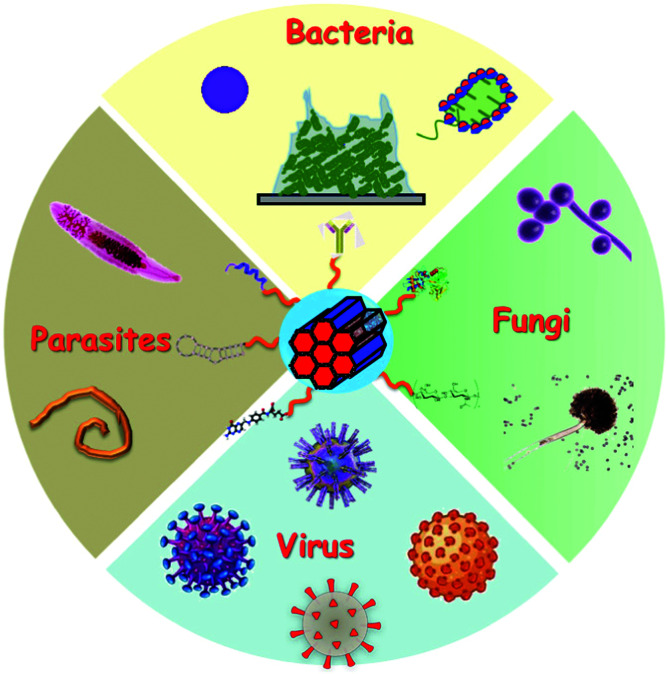
Possibilities for MSNs for the treatment of bacterial, fungal, viral and parasitic infection diseases.

MSNs as multifunctional drug delivery nanodevices for the treatment of infectious diseases have entered into this challenging scenario, bringing up the opportunity to develop custom-made therapies through the release of appropriate antimicrobial cargo, in a controlled manner only at the target infection site. Thus, innovative MSN-based formulations have been proposed for the management of infectious diseases produced by parasites, fungi, virus and bacteria (Fig. 24).

A few studies can be found in the literature applying MSNs to treat parasitic diseases. Among them, MSNs containing SWAP (soluble worm antigenic preparation)^682^ or loaded with praziquantel^683^ were evaluated to treat *Schistosoma mansoni* infection in mice; multifunctional MSNs containing benznidazole were tested against the parasite *Trypanosoma cruzi*, responsible for Chagas disease;^684^ and pH-responsive MSNs loaded with metronidazole for protozoal growth inhibition *in vitro*.^685^

Regarding fungal infections, the main research efforts have been focused on the design and development of MSN-based new formulations against *Candida albicans.* The challenge is to overcome the current drawbacks of multiple resistance mechanisms of this biofilm-forming pathogen to drugs such as fluconazole and amphotericin B.^686–688^ Diverse approaches have been suggested. They include the development of MSNs modified with Rose Bengal as photodynamic therapy (PDT) and antimicrobial treatment.^686^ Antifungals exhibiting poor aqueous solubility, such as econazol^689^ or itraconazole,^690^ were loaded into MSNs to improve drug bioavailability and increase antifungal activity. pH-Responsive tebuconazole-loaded MSNs were fabricated to treat vaginal candidiasis.^691^ Finally, loading of Ag NPs into MSNs by green synthesis, using *Azadirachta indica* leaf extract as the reducing agent, has been proposed as a promising alternative approach to the treatment of infections caused by *C. albicans*.^692^

To design and develop any viral infection treatment is essential to understand the mechanisms underlying the molecular interactions between virus and the host cells, which are key factors that govern its virulence and ability to spread. The big concern is the outstanding capability of a virus to change by genetic mutation to become drug resistant. The lack of broad-spectrum antiviral drugs, the quick and huge spreading capability of virus and the long-time required to elucidate its mechanism of action to design safe and efficient vaccines or antiviral drugs make viral infections an enormous threat to global health. Focusing on the design of novel antiviral drugs, the scientific community has dedicated impressive efforts to provide solutions to the millions of human deaths caused worldwide throughout human civilisation, such as the current pandemic situation caused by coronavirus disease 2019 (COVID-19) caused by SARS-CoV-2.^693,694^ Since the aspects related to the design and development of vaccines will be tackled in another section, here we will just give an overview of the potential of MSNs as nanocarriers for antiviral drugs to treat viral infections. In a pioneering study, LaBauve *et al.* designed lipid-coated MSNs as nanocarriers of the ML336 antiviral for Venezuelan equine encephalitis virus (VEEV).^695^ The smart design of this nanosystem overcomes the great limitations of this drug for clinical translation, such as poor solubility and stability in biological media, showing promising results both *in vitro* and *in vivo* in VEEV infected mice. In another study, Le *et al.* designed glycosaminoglycans (GAG)-mimetic-functionalized MSNs to treat Herpes simplex virus (HSV), namely HSV-1 and HSV-2, infections.^696,697^ In the first study,^697^ aryl sulfonate GAG mimetic-modified MSNs exhibited low toxicity, and most importantly inhibited HSV-1 and HSV-2 penetration into healthy cells whereas controls were inactive. In the second work, Lee *et al.*^696^ explored distinct functional groups related to the GAG structure attached to MSNs and studied their antiviral action against HSV-1 and HSV-2. Moreover, they evaluated GAG mimetic-functionalized MSNs as smart nanocarriers for acyclovir (ACV) delivery to simultaneously achieve inhibition of viral penetration and DNA replication. *In vitro* assays towards HSV-1 and HSV-2 opened an interesting alternative that can circumvent the current limitations of ACV and related compounds, such as antiviral drug resistance mostly in immunocompromised persons and poor bioavailability, which required the administration of high doses. However, as it will be discussed, MSNs are promising materials for developing vaccine delivery systems, and actually, hollow MSNs have been loaded with porcine circovirus type 2 (PCV2-ORF2) proteins to provoke specific antibodies and cell-mediated immune responses with no cytotoxic effects *in vivo*.^698^

**Fig. 25:**
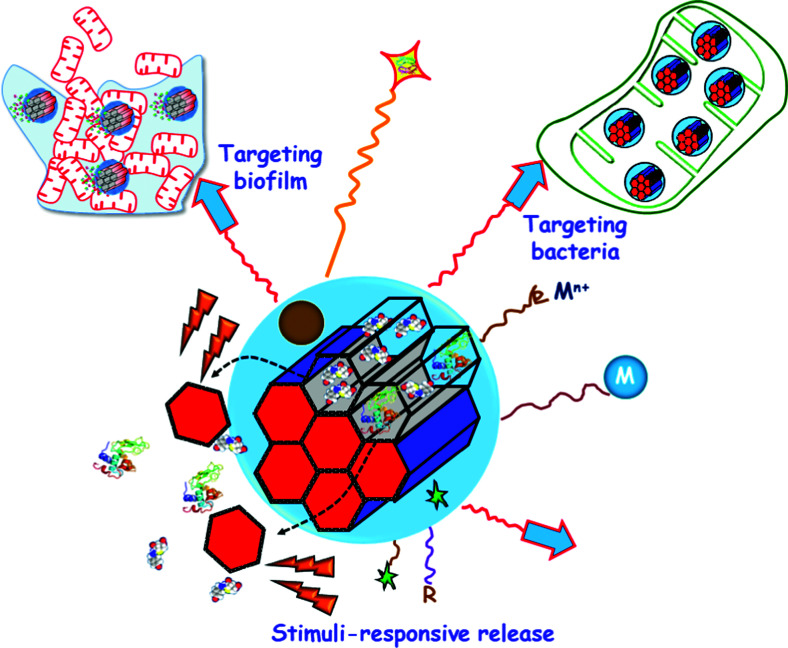
Multifunctionality in MSNs for bacterial infection treatment. Targeting ligands towards bacteria and/or biofilm (blue arrows) can decorate the external surface. Antimicrobial drugs (antibiotics) and/or antibiofilm agents (proteins and peptides) can be either adsorbed into the mesopores or else grafted to the silica walls. Stimuli-responsive pore capping agents (red nanocaps) can close the pores to prevent premature cargo release. The presence of internal (*e.g.* bacteria, pH, enzymes, redox potential) or external (*e.g.* heat, light, alternating magnetic fields (AMF)) stimuli (orange rays) provokes pore opening and cargo release. Antimicrobial metal NPs (M) and ions (M^*n*+^) can be embedded into the mesoporous structure or grafted to the outer surface of MSNs. Biocompatible hydrophilic polymers (in orange) can be grafted to the external surface to provide “stealth” properties. External grafting of organic groups (R) permits tuning the surface charge. Magnetic NPs and fluorescent molecules (green stars) can be also incorporated.

Finally, we will focus on bacterial infections, which are caused by different pathogenic bacteria and have threatened the health and lives of people all over human history, leading to plague and tuberculosis that caused many human deaths over centuries. During the last century, the development of diverse antibiotics noticeably decreased mortality, but the extensive inappropriate and long-term use of antibiotics has produced the rapid global rise and expansion of pathogenic multi- and pan-resistant bacteria (also known as “superbugs”) that cause infections untreatable with common antibiotics.^681,699^ In addition to the acquired AMR, bacteria have a natural defence mechanism called biofilms, which consist of bacterial communities that grow adhered to a surface and then are embedded in a protective self-produced extracellular matrix mainly composed of secreted polysaccharides.^700,701^ The bacterial biofilms provide protection to the inner bacteria from hostile environments, including antimicrobial agents and immune system.^702,703^ Considering that over 60–80% of chronic infections are associated with biofilms, the combination of AMR and bacterial biofilms becomes a severe clinical concern.^704^ Another big concern is the absence of new classes of antibiotics in the pipeline,^705^ which forces to treat the current bacterial infections with the antibiotics discovered until the early 1980s.^706^ To achieve antimicrobial effectivity of the existing antibiotics, high doses or several administrations of antibiotics are required, which does not only increase the toxicity and side effects but also elicit AMR. In this scenario, there is an urgent need of developing new therapeutic approaches based on drug delivery carriers with great membrane permeability and biofilm penetrability, to achieve high local antibiotic concentrations and prolonged circulation time. Nanotechnology has entered this arena, providing potent tools to engineer nanocarriers as efficient nanoformulations to fight bacterial infections. These NPs are foreseen as targeted nanomedicines for local treatments exhibiting high antimicrobial effect at low doses, and minimizing toxicity and side effects. Among different nanocarriers, MSNs exhibit unique properties for the assembly of multiple functions to treat bacterial infection. These elements include targeting elements for selective transport of antimicrobial agents to the site of infection, stimuli-responsive release capability without premature cargo leakage and the possibility of combination with other therapeutic approaches, such as photodynamic therapy (PDT), photothermal therapy (PTT), *etc.* as will be discussed in the following sections (Fig. 25).

#### 4.3.1. Targeted MSNs for bacterial infection

The use of targeted MSNs represents a powerful alternative in the management of bacterial infection. The challenge is to circumvent the two main problems associated with such diseases, antimicrobial bacterial resistance and biofilm formation. Delivering antimicrobials only at the target site without affecting healthy cells aims to increase the selectivity and efficacy of the treatment by reducing antibiotic doses, frequency of the treatment and side effects. Although insights on the EPR effect in bacterial infection have been described,^707,708^ passive targeting of antimicrobial nanosystems has been scarcely exploited. On the other hand, active targeting strategies based on decorating the outermost surface of MSNs with targeting ligands that provoke selective accumulation in the bacteria wall or the biofilm are acquiring growing relevance. The major breakthrough of this approach is the possibility to improve the efficacy of antimicrobials due not only to the specific interaction of the MSNs with bacteria or biofilm, but also to the combination with additional action mechanisms ascribed to the nanocarrier itself, such as the destabilization of the bacteria wall or the boosting of biofilm penetrability.^709^ This section describes the recent scientific developments in MSNs to address the two main targets related to bacterial infection treatment, the bacterium and the biofilm.

##### Targeting bacteria

Bacteria targeting strategies encompass free floating or planktonic bacteria, *i.e.* isolated free-living bacteria. Active targeting provides MSNs of specificity to the infection site, which can be used to distinguish between bacteria and healthy cells. This is of leading relevance in the case of intracellular infections, where bacteria overcome host immune defences and ensure their own survival within human host cells.^710^ Different strategies have been proposed to decorate the outermost surface of MSNs with target specific ligands that recognize bacteria but that do not recognize human host cells. The principal difference between bacterial and human cells is that bacteria generally have a cell wall. The bacterial cell wall is a protective, resistant and flexible layer, which participates in the bacterial growth, and this is mainly composed of peptidoglycan and other glycolipids exclusive of bacteria. These distinctive components make them great targets in bacteria.^711^ Furthermore, such components are so exclusive that they even are different depending on the type of bacteria. Thus, bacteria are classified in two great groups, Gram-positive (G^+^) and Gram-negative (G^−^) bacteria, corresponding to the structure of their cell wall. The cell wall of G^+^ bacteria consists of a double layer composed of the cytoplasmic membrane and a thick layer of peptidoglycan containing teichoic acids.^712^ In the case of G^−^ bacteria, there is a triple protection: the cytoplasmic membrane; a thinner but more rigid peptidoglycan layer with shorter cross-links; and an extra hydrophobic lipid bilayer, termed as outer membrane, composed of lipopolysaccharides (LPS). This outer membrane is the reason of the great resistance of G^−^ bacteria to numerous antimicrobials.^713^ Accordingly, selecting a suitable targeting moiety does not only permit to distinguish between bacteria and human cells, but also to direct the MSNs to a specific type of bacteria. The bacteria-targeted MSN-based drug delivery systems reported to date are summarized in Table 4.

**Table 4:** Bacteria-targeted MSNs as antimicrobial delivery systems

Targeting ligand^a^	Bacteria^b^	Drug loaded^c^	Nanocarrier^d^	Assay	Ref.
FB11	*F. tularensis*	Model drugs (Fluorescein, Hoechst 33342)	MCM-41 FB11mFt LPS-MSNs	*In vitro*	529
Anti-*S. aureus* Ab	*S. aureus*	Vancomycin	Ab@S-HA@MMSNs	*In vitro*	714
SA20hp	*S. aureus*	Vancomycin	MCM-41 SA20hp-MSNs	*In vitro*	715
UBI_29–41_	*S. aureus*	Gentamicin	MSN-LU	*In vitro* and *in vivo*	716
LL-37	*P. aeruginosa*	Colistin	MSN@LL-(LL-37)	*In vitro*	717
Trehalose	*M. smegmatis*	Isoniazid	M-PFPA-Tre	*In vitro*	718
Trehalose	*M. smegmatis*	Isoniazid	Tre-HOMSNs	*In vitro*	719
Arginine	S. *typhimurium*	Ciprofloxacin	Arg-MSNs	*In vitro* and *in vivo*	720
Folic acid	*E. coli*, *S. aureus*	Ampicillin	MSN@FA@CaP@FA	*In vitro* and *in vivo*	721
Vancomycin	*S. aureus*	Vancomycin (grafted)	MCM-41 MSNs⊂VAN	*In vitro*	722
OMV	*E. coli*	Rifampicin	OMV@MSN	*In vitro* and *in vivo*	723
ε-pLys	*E. coli, S. typhi*, *E. Carotovora*	Vancomycin	MCM-41 ε-pLys-MSNs	*In vitro*	724
ε-pLys	*E. coli*, *S. marcescens*	HKAIs	MCM-41 ε-pLys-MSNs	*In vitro* and *in vivo*	725
LYS	*E. coli*, *B. safensis*	KANA	MSN–AuNC@LYS	*In vitro*	726
G3	*E. coli*	Levofloxacin	MCM-41 G3-MSNs	*In vitro*	727
G3	*E. coli*	Levofloxacin	MCM-41 M^*n*+^-G3-MSNs	*In vitro*	625

a FB11: FB11 antibody for lipopolysaccharide (LPS) present in *Francisella tularensis* (*Ft*); Anti-*S. aureus*: *S. aureus* antibody; SA20hp: SA20 aptamer with hairpin structure; UBI_29–41_: Ubiquicin; LL-37 peptide: human cathelicidin peptide; Arg: arginine; OMV: outer membrane vesicle isolated from *E. coli*; ε-pLys: ε-poly-l-lysine cationic polymer; LYS: lysozyme, and G3: poly(propyleneimine) third-generation dendrimer.

b *E. coli*: *Escherichia coli*; *S. marcescens*: *Staphylococcus marcescens*; *F. tularensis*: *Francisella tularensis*; *S. aureus*: *Staphylococcus aureus*; *P. aeruginosa*: *Pseudomonas aeruginosa*; *M. smegmatis*: *Mycobacterium smegmatis*; *S. typhi*: *Salmonella typhimurium*; *E. carotovora*: *Erwinia carotovora*; and *B. safensis*: *Bacillus safensis*.

c HKAIs: histidine kinase authophosphorylation inhibitors; and KANA: Kanamycin.

d MCM-41 FB11mFt LPS-MSNs: MCM-41 type MSNs functionalized with FB11 antibody through a derivative of the O-antigen of Ft LPS; Ab@S-HA@MMSNs: sulfonated-hyaluronic acid (S-HA) terminated magnetic MSNs modified with Anti-*S. aureus* (Ab); MCM-41 SA20hp-MSNs: MCM-41 type MSNs functionalized with SA20hp; MSN-LU: MSNs modified with a lipidic bilayer surface shell and conjugated with UBI_29–41_; MSN@LL-(LL-37): MCM-41 type MSNs coated with a lipidic layer and conjugated with LL-37; M-PFPA-Tre: perfluorophenylazide-functionalized decorated with α,α-trehalose; Tre-HOMSNs: trehalose-functionalized hollow oblate MSNs; Arg-MSN: MCM-41 type MSNs functionalized with l-Arg; MSN@FA@CaP@FA: MSNs covered by double folic acid (FA) and calcium phosphate (CaP) layers; MCM-41 MSNs⊂VAN: MCM-41 type MSNs functionalized with vancomycin, MCM-41 OMV@MSN: MCM-41 type MSN core coated by an OMV as shell; ε-pLys-MSNs: MCM-41 type MSNs functionalized with pLys; MSN–AuNC@LYS: MSNs capped with LYS-functionalized gold nanoclusters. MCM-41 G3-MSNs: MCM-41 type MSNs functionalized with G3; MCM-41; and MCM-41 M^*n*+^-G3-MSNs: MCM-41 type MSNs functionalized with G3 coordinated to M^*n*+^ (M^*n*+^ = Zn^2+^, Ag^+^).

Different strategies have been focused on the “ligand–receptor binding” concept to develop highly specific bacteria-targeted nanosystems by decorating the outmost surface of MSNs with ligands that specifically bind surface receptors or molecules overexpressed in the bacteria cell wall. These ligands consist of antibodies,^529,714^ aptamers,^715^ peptides,^716,717^ carbohydrates^718,719^ or small molecules, such as amino acids,^720^ vitamins^721^ and certain antibiotics.^722^

As an alternative approach to target specific surface receptors of bacterial cell walls, the different adsorption pathways of NPs have been also exploited.^728^ The group of Malmsten has studied the lipid membrane interactions of virus-like MSNs, which present a biomimetic “spiky” external surface.^729^ The results proved that topography strongly influences the interaction of the NPs with bacteria-mimicking lipid bilayers, as well as with bacteria, provoking membrane binding and destabilization. Finally, virus-like MSNs were loaded with the antimicrobial peptide LL-37 and tested against *E. coli* bacteria, resulting in an accentuated membrane-disruptive effect than either peptide-loaded smooth NPs or free peptide. Wang *et al.* applied the same concept to fabricate Ag nanocubes with biomimetic virus-like mesoporous silica coating loaded with gentamicin able to be efficiently adsorbed on the cell wall of both *E. coli* and *S. aureus* bacteria.^730^ The virus-like surface of this core–shell nanostructure allowed the efficient adsorption on the rigid bacteria cell wall, overcoming the low cell wall adhesion properties of antibacterial Ag NPs. Very recently, Wu *et al.* went a step further and developed an innovative biomimetic delivery nanosystem by coating rifampicin-loaded MSNs with outer membrane vesicles (OMVs) isolated from *E. coli*. Rifampicin is conventionally used to treat G^+^*S. aureus* or *tuberculosis* infections, since it hardly crosses the double-membrane barrier of G^−^ bacteria, which is associated with low antimicrobial effect. Since bacterial vesicles usually enter the same type of bacteria, and due to the similitude in their membrane structures, antimicrobial activity of OMV-coated rifampicin-loaded MSMs was evaluated *in vitro* against G^−^*E. coli*, showing enhanced uptake and antimicrobial activity.^723^

It is also feasible to take advantage of the electrostatic attractive interactions between positively charged NPs and negatively charged bacteria cell walls to favour accumulation of the former stuck to the bacteria wall, perturbing metabolic pathways or producing perforation and even membrane leakage.^731,732^ Besides, diverse studies have demonstrated that positively-charged NPs prompt internalization in both G^+^ and G^−^ bacteria.^733^ These findings opened the way to develop innovative approaches based on using positively charged MSNs as antibiotic nanocarriers to penetrate the bacteria cell wall and increase antimicrobial efficacy. For instance, Ruiz-Rico *et al.* proved that polyamine-decorated MSNs prompt cell membrane disruption in G^+^*Listeria monocytogenes*, exhibiting 100 times more antimicrobial power than free polyamines.^734^ The use of cationic polymers, such as poly-l-lysine (ε-pLys), as capping elements of MSNs, enhanced the toxicity of antimicrobials towards G^−^ bacteria. This is explained by the bacterial wall damage produced by positively-charged lysine residues, which permits loaded cargo to get access into the bacteria.^724,725^ A similar approach was developed by Alsaiari *et al.*, who reported a sophisticated smart nanosystem comprising different elements, among them the cationic lysozyme (LYS), for the detection and inhibition of bacteria.^726^ The mechanism of action relied on the electrostatic interaction between positively charged LYS and the negatively charged bacterial cell wall, as it will be described in the section of stimuli-responsive systems.

**Fig. 26:**
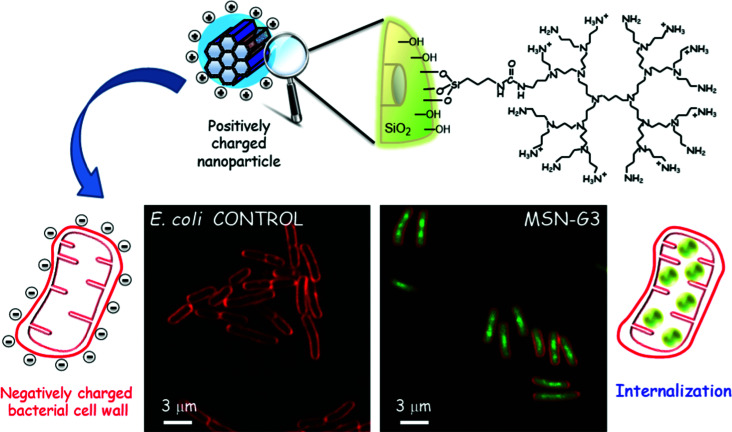
Top: schematic depiction showing positively charged nanosystem consisting of MSNs externally decorated with the poly(propyleneimine) dendrimer of third generation (G3) by covalent grafting and its interaction with G^−^*E. coli* bacteria. The electrostatic attractive forces between a positively charged dendrimer on the surface of MSNs and negatively charged bacterial cell wall provokes cell membrane disruption and internalization of the nanosystem. Bottom: confocal microscopy images showing *E. coli* control culture in the absence (control, left) or presence of 10 μg mL^−1^ of the MSN-G3 nanosystem (right) during 90 min of incubation time. The *E. coli* cell membrane is stained in red and the MSNs are labelled in green using a fluorescent dye.^727^ Adapted with permission from ref. 727. Copyright 2018, Elsevier Ltd.

In this context, Vallet-Regí's group proposed a new approach to develop innovative “nanoantibiotics”. Thus, MSNs were loaded with levofloxacin (LEVO) and then externally functionalized with a polycationic poly(propyleneimine) dendrimer of third generation (G3) as the targeting ligand.^625,727^ Internalization studies demonstrated that the polycationic G3 dendrimer grafted to MSNs prompted penetrability throughout the G^−^*E. coli* bacterial membranes (Fig. 26). The flexibility and high density of positive charges provided by G3 on the surface of MSNs permit a close attractive interaction with the negatively charged bacterial wall, producing membrane permeabilization and thus favouring nanoantibiotic internalization. These studies demonstrated that merging the cell wall disruption ability of G3 with the bactericide effect of antibiotics,^727^ or antibiotics and some metal cations such as Zn^2+^ or Ag^+^,^625^ leads to synergistic antimicrobial effects on G^−^ bacteria.

##### Targeting biofilms

Another crucial challenge that society has to face in the fight against bacterial infections is the capability of bacteria to associate in communities forming biofilms, which hampers conventional treatments for chronic infections and has severe socio-economic consequences. Bacteria in biofilms are embedded in a self-produced protective exopolysaccharide (EPS) matrix composed of extracellular DNA, polysaccharides, proteins, glycolipids and other ionic molecules.^700^ The EPS matrix is responsible for intercellular interactions and defense of bacterial cells from harsh environmental conditions, making bacteria in biofilms up to 1000 times more resistant to antibiotics than planktonic bacteria.^735,736^ Biofilm formation on any surface involves multiple stages. Firstly, planktonic bacteria adhere to a surface. Then, bacteria multiply and form well-defined 3D microcolonies while secreting the EPS matrix around them. Every so often, the biofilm matrix breaks and disperses cells into the environment to colonize new sites and spread the infection.^737,738^

Nanotechnology-based strategies aimed at treating biofilm-related infections are different from those directed to bacteria in the planktonic state. Targeting bacterial biofilms with nanocarriers capable of disrupting the EPS barrier, penetrating bacterial biofilm and releasing the antimicrobial cargo, constitutes a promising alternative against bacterial biofilms. This research field is still in its infancy, and there are relatively few publications on this topic. Table 5 summarizes biofilm-targeted MSNs as antimicrobial release systems.

**Table 5:** Bacterial biofilm-targeted MSNs as antimicrobial delivery systems

Targeting ligand^a^	Bacterial biofilm^c^	Drug loaded	Nanocarrier^b^	Assay	Ref.
DAMO	*S. aureus*	Levofloxacin	MCM-41 DAMO-MSNs	*In vitro*	727 and 739
G3	*E. coli*	Levofloxacin	MCM-41 G3-MSNs	*In vitro*	727
G3	*E. coli*	Levofloxacin	MCM-41 M^*n*+^-G3-MSNs	*In vitro*	625
ConA	*E. coli*	Levofloxacin	MCM-41 ConA-MSNs	*In vitro*	740
Arabic gum	*E. coli*	Moxifloxacin	MCM-41 AG-CO@MSNs	*In vitro & in vivo*	741

a DAMO: *N*-(2-aminoethyl)-3-aminopropyltrimethoxy-silane; G3: poly(propyleneimine) third-generation dendrimer; and ConA: concanavalin A.

b MCM-41 DAMO-MSNs: MCM-41 type MSNs functionalized with DAMO; MCM-41 G3-MSNs: MCM-41 type MSNs functionalized with G3; MCM-41 M^*n*+^-G3-MSNs: MCM-41 type MSNs functionalized with G3 coordinated to M^*n*+^ (M^*n*+^ = Zn^2+^, Ag^+^); MSNs-ConA: MCM-41 type MSNs decorated with ConA; and AG-CO@MSNs: MSNs coated with Arabic gum containing colistin.

c *E. coli*: *Escherichia coli*; and *S. aureus*: *Staphylococcus aureus*.

Some strategies are focused on the design of MSNs as delivery systems of antibiofilm agents such as certain enzymes, for instance lysozyme^742^ or DNase I,^743^ able to decrease EPS cohesiveness and thus reduce and disperse the established biofilm mass. In addition, since the EPS constituents are typically negatively charged, increasing the nanoparticle-biofilm interactions by tuning the surface charge of MSNs is an interesting option. For example, vancomycin-loaded MSNs exhibiting a positive surface charge were more efficiently located on the surface of biofilm cells and were more active in reducing biofilm cell viability than negatively charged MSNs, even though the amount of vancomycin loaded was higher in the latter.^744^ Vallet-Regí and co-workers also applied this concept to design and engineer new nanoantibiotics consisting of LEVO-loaded MSNs externally decorated with ligands bearing amino groups as biofilm-targeting ligands.^727,739^ Amine-functionalization provided MSNs of positive charges in the physiological medium, prompting the capability of the nanoantibiotic to target and penetrate bacterial biofilms. In this context, the functionalization with N-(2-aminoethyl)-3-aminopropyltrimethoxy-silane improved the affinity of the nanosystem towards G^+^*S. aureus* bacterial wall and biofilm, provoking an almost complete biofilm eradication when the antibiotic and the targeting agent are combined in the same nanoplatform (Fig. 26).^739^ On the other hand, great antibiofilm efficacy against G^−^*E. coli* was attained through the synergistic combination of polycationic dendrimers (G3), as bacterial membrane permeabilization and biofilm-targeting agents, and LEVO molecules as the antibiotic cargo in MSNs.^727^

**Fig. 27:**
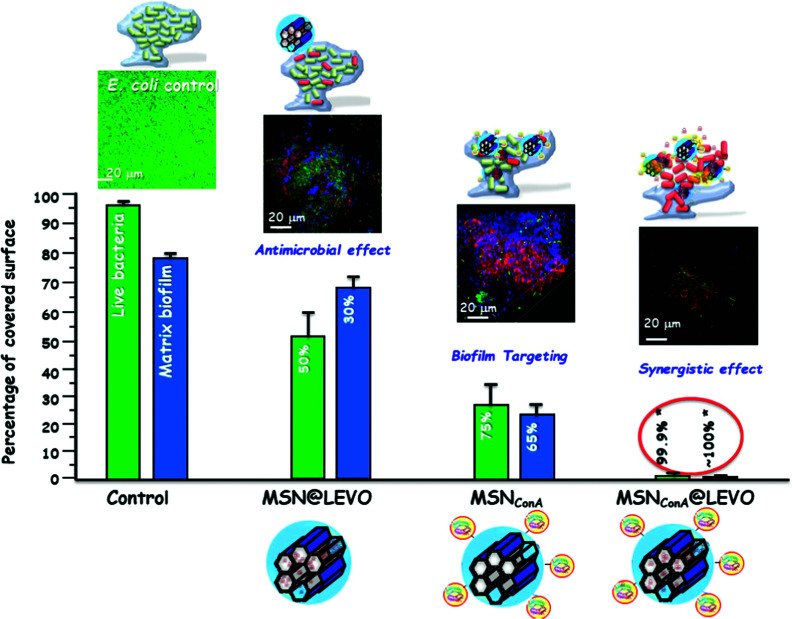
Antimicrobial efficiency of levofloxacin (LEVO) loaded and concanavalin A (ConA) decorated MSNs against the *E. coli* biofilm. The histograms represent the reduction in the percentage of covered surface of live bacteria (green bars) and mucopolysaccharide matrix (blue bars), determined from the confocal microscopy images (top). The images were acquired after the exposure of a preformed *E. coli* biofilm to the different nanosystems for an incubation period of 90 min. Live bacteria are stained in green, dead bacteria in red, and the protective matrix biofilm in blue. A synergistic antibiofilm effect is observe when the MSNs are loaded with LEVO and decorated with ConA, this latter acting as targeting ligand towards the mucopolysaccharide matrix and allowing the release of the antibiotics inside the biofilm.^740^ Adapted with permission from ref. 740. Copyright 2019, Elsevier Ltd.

Another biofilm-targeting approach was based on decorating the external surface of MSNs with molecules exhibiting affinity towards certain constituents of the EPS. For instance, concanavalin A (ConA) lectin, which is a protein that recognize and binds to glycan-type polysaccharides present in the biofilm EPS, was used as the targeting agent.^740^ The outer surface of MSNs was functionalized with carboxylic acid groups, decorated with ConA and loaded with LEVO. The targeting capability of the nanosystem was evaluated in *E. coli* biofilms proving that the presence of ConA decorating the external surface of the nanosystem favors its internalization into the biofilm matrix in a dose dependent manner. The release of the antibiotic cargo from MSNs into the biofilm was prompted by ConA, which increased the antimicrobial power of the antibiotic. Actually, the synergistic combination of ConA and LEVO in a unique nanoplatform resulted in total biofilm destruction (Fig. 27).^740^

Very recently, a similar biofilm-targeting strategy was reported using Arabic gum (AG) to coat MSNs.^741^ AG is a branched-chain, complex polysaccharide composed of 1,3-linked beta-d-galactopyranosyl monomers linked to the principal chain through 1,6-linkages [27] whose degradation by secreted bacterial enzymes enhances the retention of MSNs on the biofilm. The nanosystem demonstrated high affinity toward an *E. coli* biofilm matrix, thanks to the AG shell, and remarkable antibacterial activity due to the combined effect of two antibiotics: moxifloxacin loaded in MSNs, with bactericidal effect, and colistin, entrapped into the AG coating, with disaggregating effect. More importantly, this nanosystem shows substantial efficacy *in vivo* against an infection provoked by *E. coli* in a rabbit model of implant-associated osteomyelitis, where the nanosystem was able to eliminate more than 90% of the bacterial load within the infected bone.^741^

#### 4.3.2. Stimuli-responsive MSNs for bacterial infection treatment

When focusing on bacterial infection treatment, MSN-based nanocarriers should load, protect and transport the antimicrobial cargo to the target infection site and once there, upon exposure to a certain trigger, release the antimicrobial payload. Although stimuli-responsive MSN-based nanosystems have been widely exploited for antitumor therapy, their application in the treatment of bacterial infection is still at its infancy.^175,461,466,745–751^

Both, internal stimuli such as certain biological cues characteristic of the bacterial infected microenvironment, and external non-invasive remote stimuli have been explored as release triggers of antimicrobials from MSNs. The emerging, innovative and talented nature of these smart nanosystems make them worth of being reviewed in detail, and it will be the scope of the following sections.

#### 4.3.3. Internal stimuli-responsive MSNs

##### Presence of bacteria

The presence of bacteria is an internal stimulus exclusively proposed for infection treatment and provides the advantage of using the pathogen agent responsible for the disease itself to trigger antimicrobial release from MSNs.

**Fig. 28:**
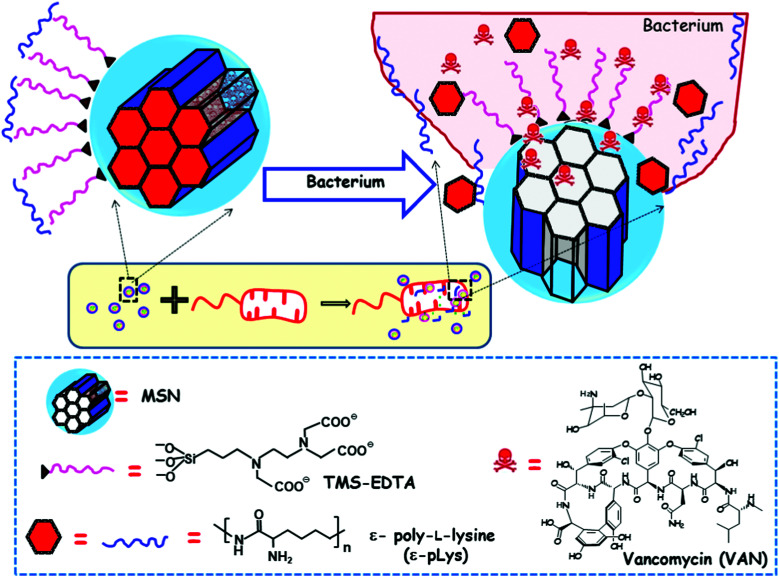
Schematic illustration of the mechanism of action of bacterial-responsive antimicrobial nanosystem consisting of MSNs loaded with vancomycin (VAN), externally decorated with *N*-[(3-trimethoxysilyl)propyl] ethylenediamine triacetic acid trisodium salt (TMS-EDTA) and capped with polycationic polymer ε-poly-l-lysine (ε-pLys). ε-pLys acts as the gatekeeper by interacting electrostatically with the negatively charged TMS-EDTA in the surface of MSNs. Upon exposure to bacteria, ε-pLys adheres to the negatively charged bacterial cell wall, provoking pore uncapping and VAN release.^725^ Adapted with permission from ref. 725. Copyright 2013, Wiley-VCH GmbH.

In this context, Velikova *et al.* reported an innovative approach consisting of grafting *N*-[(3-trimethoxysilyl)propyl] ethylenediamine triacetic acid trisodium salt (TMS-EDTA) to the outer surface of vancomycin (VAN)-loaded MSNs to provide them with negative surface charges.^725^ Then the positively charged cationic polymer poly-l-lysine (ε-pLys) was incorporated into the system through electrostatic interactions, acting not only as a capping agent but also as a bacteria targeting ligand, as discussed above.^724^ The stimuli-responsive release mechanism, schematically illustrated in Fig. 28, relies on the affinity of the positively charged ε-pLys blocking caps towards the negatively charged bacterial cell wall, whose adhesion triggers pore opening and cargo release. The nanosystem was *in vitro* evaluated against G^−^*E. coli*, *S. typhi and E. carotovora*, showing a synergistic antimicrobial effect in terms of growth inhibition and cell viability due to the combination of ε-pLys and VAN in a unique nanoplatform, which is not observed when these elements are independently tested. Furthermore, the bacterial cell wall damage produced by ε-pLys adhesion favors VAN penetration and broadens the antibacterial spectrum of such antibiotic, whose sole administration usually triggers bacterial resistance in G^−^ bacteria.

Analogous stimuli-responsive nanosystem design was applied by the same research group to evaluate the antimicrobial activity of histidine kinase autophosphorylation inhibitors (HKAIs) loaded into the mesopores, against *E. coli* and *S. marcescens* G^−^ bacteria.^725^ The observed bactericidal activity against G^−^ bacteria was attributed to the enhanced delivery and internalization of HKAIs. Besides, this nanosystem did not exhibit adverse effects on mammalian cells or the immune function of macrophages *in vitro* and showed no signs of toxicity to zebrafish larvae *in vivo*.

**Fig. 29:**
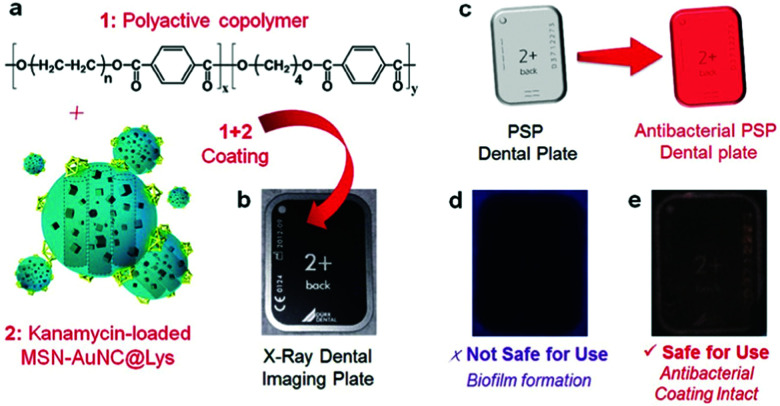
(a) Fabrication of the polyactive copolymer embedding the KANA-loaded MSN–AuNC@LYS nanofiller (b) for coating an X-ray dental plate device (photostimulable phosphor plate, PSP). This coating provides the device with (c and d) antibacterial and (d and e) bacterial contamination detection capabilities. The bacterial contamination of the medical device is easily detected by the direct visualization of the color change of the PSP dental plate upon exposure to UV light.^726^ Reproduced with permission from ref. 726. Copyright 2018, Wiley-VCH GmbH.

In another innovative research, Alsaiari *et al.* reported the development of a smart mixed-matrix membrane coating for X-ray dental imaging devices, with the capability of detecting and inhibiting healthcare-associated infections without particle leaching (Fig. 29a and b).^726^ This coating consisted of a poly(ethylene oxide)/poly(butylene terephthalate) (PEO-PBT, commercial name “polyactive”) comprising uniformly dispersed nanofillers. The nanofillers consisted of MSNs bearing positive surface charges loaded with kanamycin (KANA) and capped with negatively charged gold nanocluster–lysozyme (AuNC@LYS) colloids, which exhibit an intense red fluorescence under UV light. The presence of *E. coli* resulted in detachment of clusters from silica particles, due to the interaction of LYS with the bacteria cell wall, causing quenching of the red fluorescence and simultaneously releasing the antimicrobial cargo. Bacteria-responsive antibacterial efficacy was evaluated against G^−^*E. coli* and G^+^*B. safensis*, showing reproducible inhibition of bacterial growth. The practical utility of this coating was to provide common X-ray dental imaging plates, which are prone to oral bacteria contamination, with sensing and antimicrobial capabilities (Fig. 29c). The lack of blue fluorescence of the plate surface under UV light signals bacterial contamination (Fig. 29d). On the other hand, red fluorescence surface indicates a bacteria-free environment (Fig. 29d). It should finally be mentioned that this innovative strategy could be leveraged to diverse medical devices without disturbing their function.

The major limitation of the nanosystems described above is their non-specificity, which hinders their application in the detection and treating of a specific pathogen. As an alternative approach, Kavruk *et al.* designed aptamer-gated VAN-loaded MSNs capable of releasing the antibiotic cargo upon ligand–receptor interactions with specific antigens present on the surface of *S. aureus* bacteria.^715^ The gatekeeping procedure consisted of grafting *S. aureus* specific SA20 aptamer to VAN-loaded MSNs and then converting it to the hairpin structure (SA20hp). The interaction of SA20hp with the surface antigens present on the surface of *S. aureus* produces a rearrangement of the aptamer structure, triggering pore uncapping and VAN release. The antimicrobial efficiency of the nanosystems was evaluated against *S. aureus* and *S. epidermidis*, as targeted and non-targeted bacteria, respectively. This *in vitro* test revealed a 15-fold higher antimicrobial efficacy of the nanosystem against *S. aureus* than against *S. epidermidis*, which is due to the targeting and appropriate for the former bacteria with their higher toxicity. Actually, *S. epidermidis* was harmed by using relative high doses of the nanosystem (6.295 μg mL^−1^). This study reveals that it is feasible to administer appropriate doses (*e.g.*, 0.420 μg mL^−1^) to selectively eradicate the target pathogen (*S. aureus*) without damaging the non-target bacteria (*S. epidermidis*).

Ruehle *et al.* also reported an attractive approach to engineer a pathogen-targeted detection and release nanoplatform based on highly specific antigen–antibody interactions.^529^ During this study, they chose the FB11 antibody, with high affinity towards antigens of the LPS of pathogenic *Francisella tularensis* (*Ft*), for the treatment of lethal pneumonic tularemia. Thus, MSNs were loaded with fluorescein, as model molecule, decorated with the O-antigen of the LPS of *Ft*, used as a model molecule, and finally capped with the FB11 antibody. Upon exposure to the target *Ft* bacteria, the native antigen of the LPS shows greater affinity towards the capping FB11 antibody. Therefore, the antibody capping agent is competitively displaced, and the release of the cargo loaded into the mesopores takes place. Fluorescein allowed monitoring the behavior of the nanosystem by measuring the fluorescence intensity after incubation with target *Ft* and non-target *Francisella novocida* (*Fn*) bacteria. The results revealed that the intensity detected in *Ft* was 5 times higher than that in *Fn*, demonstrating the good selectivity of this nanosystem.

##### Bacterial toxins

**Fig. 30:**
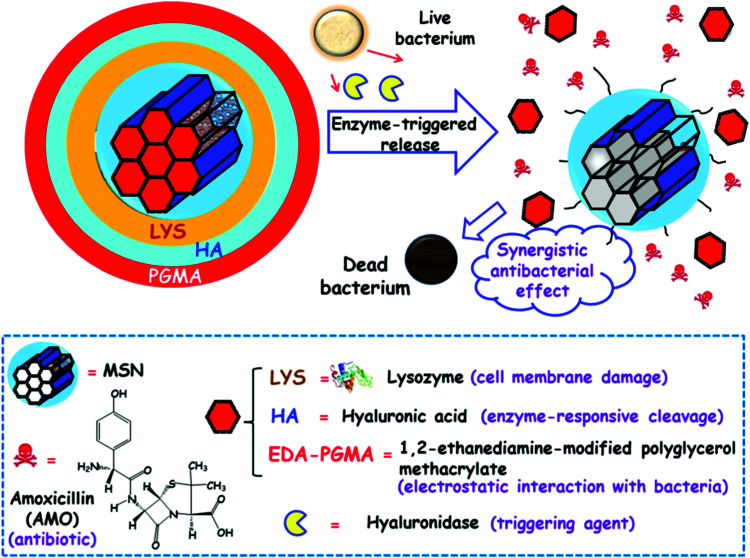
Schematic depiction of the mechanism of action of an enzyme-responsive antimicrobial MSN-based biohybrid nanomaterial. The nanosystem was crafted by loading amoxicillin (AMO) into the pores of MSNs and a subsequent coating by layer-by-layer (LBL) self-assembly of lysozyme (LYS), hyaluronic acid (HA) and 1,2-ethanediamine-modified polyglycerol methacrylate (EDA-PGMA). The cationic polymer incorporated into the nanosystem favours its interaction with bacteria. As the nanosystem comes across the bacteria, the presence of hyaluronidase secreted by bacteria triggers the cleavage of the HA protective coating. Then, LYS and AMO can bind to bacteria efficiently and quickly harming the cell membrane of bacteria and synergistically causing bacterial death.^753^ Adapted with permission from ref. 753. Copyright 2015, The American Chemical Society.

Other research efforts were directed to the design of MSNs that release the antimicrobial cargo upon exposure to high levels of certain toxins produced and secreted by bacteria in septic microenvironments.^752^ In this context, Wu *et al.* engineered biohybrid nanomaterials that release the antimicrobial payload in the presence of hyaluronidase (Hyal), an enzyme produced by several pathogenic *S. aureus* bacteria.^753^ The biohybrids consisted of antibiotic-loaded MSNs that were sequentially coated with different functional protective layers using the layer-by-layer LBL self-assembly method (Fig. 30). Amoxicillin (AMO)-loaded MSNs were externally decorated with carboxylate groups for the later adsorption through electrostatic interactions with the positively charged LYS. This enzyme is mainly bacteriolytic towards G^+^ bacteria, because of the protective LPS layer surrounding the outmost membrane of G^−^ bacteria. Then, the resulting nanosystem was coated with negatively charged hyaluronic acid (HA), as the enzyme responsive layer. Finally, the outermost layer was added, consisting of the cationic polymer 1,2-ethanediamine-modified polyglycerol methacrylate (EDA-PGMA). This does not specifically target the bacteria, but it is able to undergo electrostatic attracting interactions with the negatively charged bacterial cell wall. The EDA-PGMA polymer and LYS incorporated in the nanosystem allow for the efficient binding onto the cell membrane of both G^−^*E. coli* and G^+^*S. aureus* bacteria, owing to a multivalent interaction. The HA layer acts as a Hyal-responsive valve for AMO release. The combination of AMO, LYS, HA and EDA-PGMA into a unique mesoporous silica nanoplatform revealed a strong synergistic effect and efficient antibacterial capability to AMO-resistant bacteria compared to the free antibiotic *in vitro*. Moreover, *in vivo* assays in a mouse wound model infected with *S. aureus* demonstrated superior antimicrobial inhibition, adequate biocompatibility and negligible hemolytic side effects.

In another report, Xu *et al.* exploited the Hyal-triggered antibiotic release concept in the design and construction of an “on-demand” integrated platform for the diagnosis and treatment of *S. aureus* bloodstream infection.^714^ In this study, magnetic MSNs were loaded with VAN, functionalized with sulfonated-HA, and superficially decorated with a *S. aureus* antibody as the targeting agent. With the aid of magnetic interaction, the resulting nanodevice was then dropped onto the surface of magnetic glassy carbon electrode (MGCE). In presence of the *S. aureus*, specific antigen–antibody binding takes place between *S. aureus* in solution and the antibody-modified MGCE. This results in variations of the electrochemical signals, which allowed the accurate detection of the amount of the target bacteria in solution. Sulfonate-HA functionalizing magnetic MSNs has anticoagulant properties, providing the resulting platform with antiadhesion properties and allowing the detection of the amount of *S. aureus* in whole blood. In addition, as the amount of *S. aureus* arriving at the MGCE increased, the HA-based capping agent degraded by action of Hyal secreted by bacteria. This triggered pore uncapping and subsequent VAN release, which eventually kills *S. aureus*.

**Fig. 31:**
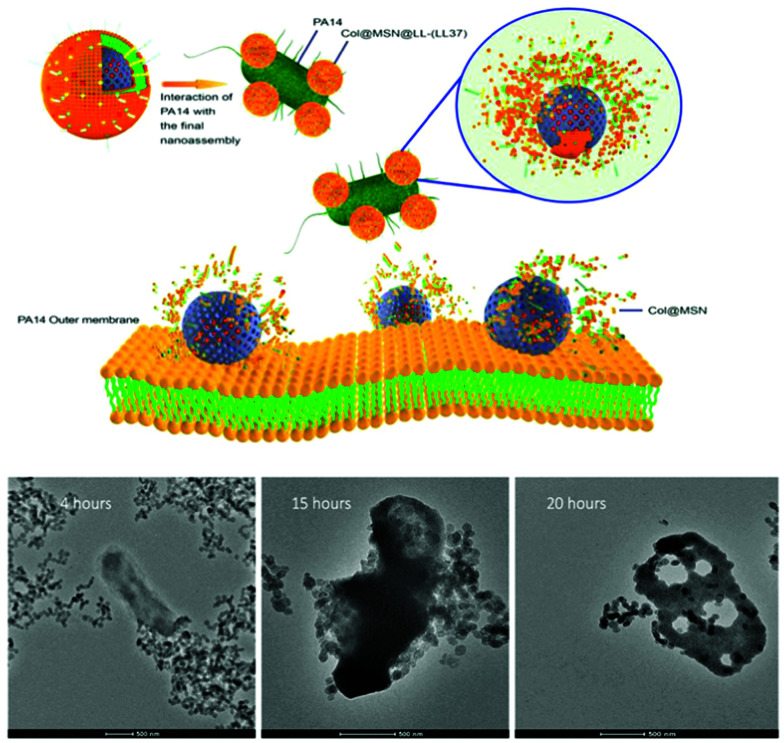
Up: schematic depiction of the hypothetical mechanism of action of the bacteria-targeted and enzyme-responsive nanosystem denoted as Col@MSN@LL-(LL-37). The nanoassembly consists of colistin (Col)-loaded MSNs wrapped by a liposomal layer (LL) and decorated with the antimicrobial peptide LL-37, which is able to recognize the outer membrane of G^−^*Pseudomonas aeruginosa* (PA). The liposomal shell prevents premature Col release before the nanosystem approaches the target bacteria, the clinical strain PA14, and degrades upon excreted lipase present in the local environment of PA, releasing encapsulated Col. Bottom: TEM images showing the interaction of Col@MSN@LL-(LL-37) with PA14 monitored for 4–20 h (scale bars represent 500 nm). This *in vitro* study shows significant bacterial PA14 degradation within 20 h following interaction. Adapted with permission from ref. 717. Copyright 2020, The American Chemical Society.

Furthermore, other enzymes secreted by bacteria, such as lipases, phosphatases and phospholipases, have been proposed as release triggers in the design of new smart nanodevices to fight intracellular bacteria.^716,717^ The original design of these intracellular antibiotic delivery nanosystems relied on coating antibiotic-loaded MSNs with a lipid bilayer shell and decorating the outermost surface with a specific bacteria-targeting antimicrobial peptide. The targeting agent permits the recognition of the specific bacterial cell, whereas the lipid material acts as the capping component that protects antibiotic molecules from inactivation and prevents their release before MSNs reach the target bacteria. Once there, bacterial toxins degrade the lipid bilayer that quickly releases the drug, efficiently killing the bacteria (Fig. 31).^717^

##### pH

In the same way as that in some tumor tissues, bacterial infection causes a local pH drop through anaerobic fermentation, triggered by low oxygen concentrations^754^ and inflammatory responses elicited by the host immune system.^755^ Since the physiological pH of 7.4 can drop to values as low as 5.5 at the infection site,^756^ this fact has been exploited to develop new nanotherapeutics against bacterial infection.

Kuthati *et al.* reported the design and synthesis of pH-triggered nanoconjugates through the grafting of a silver complex (silver-indole-3 acetic acid hydrazide, IAAH-Ag), as a model drug, to the inner mesopore surface of MSNs, *via* pH-sensitive hydrazone bonds.^757^ When the nanoconjugates were exposed to acidic pH (near pH 5.0), cleavage of hydrazone bonds allowed the release of significant silver ions (70%) in a controlled fashion over up to 12 h. In contrast, lower levels of ions (about 25%) were released in physiological buffer (pH 7.4). In addition, enhanced antimicrobial efficacy was obtained against MDR G^+^ and G^−^ planktonic bacteria (*E. coli* and *S. aureus*) and biofilm (*E. coli*, *B. subtilis*, *S. aureus* and *S. epidermidis*) *in vitro*. Furthermore, good *in vivo* antibacterial efficacy was demonstrated by an intraperitoneal *E. coli* infection route in an adult mouse model. While promising results were obtained using IAAH-Ag as the model drug, the versatility of the immobilization method suggests the possibility to extend this approach to a wide variety of antimicrobial cargo for pH-sensitive drug release.

**Fig. 32:**
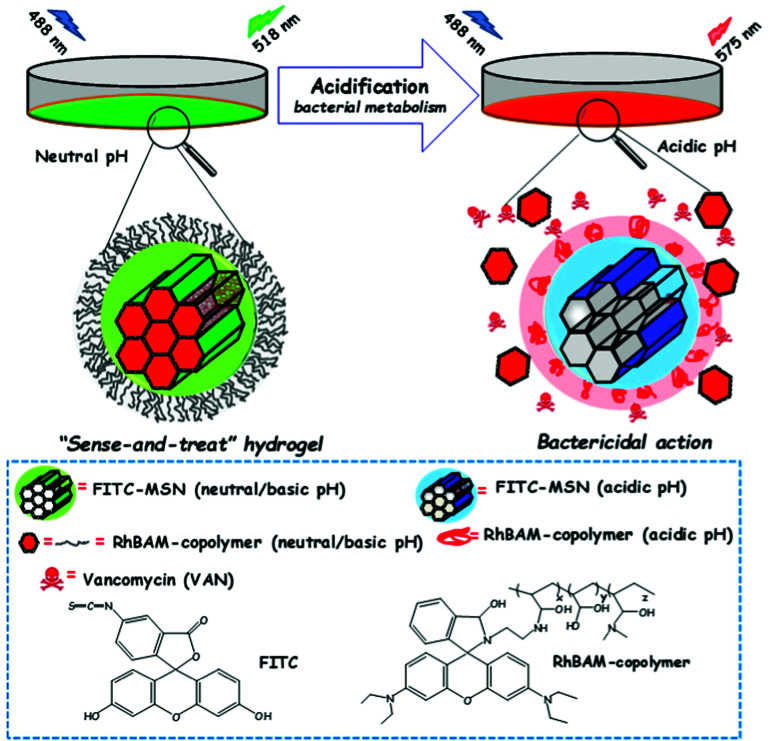
Schematic depiction of the operating mechanism of the pH-responsive “sense-and-treat” hydrogels for sensing and killing bacteria.^736^ Adapted with permission from ref. 758. Abbreviations: FITC: fluorescein-isothiocyanate, RhBAM: rhodamine B-derivative, PNIPAAm: poly(*N*-isopropyl acrylamide-*co*-acrylic acid).

In another study, Yan *et al.* developed a sophisticated “sense-and-treat” hydrogel for the detection and killing of bacteria.^758^ Initially, fluorescein isothiocyanate (FITC)-labeled MSNs were loaded with VAN. The resulting VAN-loaded FITC-MSNs were then grafted with a copolymer synthetized by copolymerization of rhodamine B-based derivative (RhBAM) with the pH-sensitive polymer poly(*N*-isopropyl acrylamide-*co*-acrylic acid) (PNIPAAm). In this nanosystem, FITC and RhBAM make up the radiometric fluorescent probes. Thanks to the pH-sensitive properties of FITC, the nanosystem has a strong green fluorescence at 518 nm under basic or neutral pH conditions, while the emission decreases as the pH drops to acidic values (Fig. 32). In contrast, the RhBAM moiety exhibits no fluorescence at neutral or basic pH, whereas it emits strong red fluorescence at 575 nm under acid pH. On the other hand, the copolymer on mesoporous silica acted as the pH-responsive pore-blocking agent. Thus, it first swelled at the physiological pH and then it started to shrink in the acidic environment, allowing pore uncapping and VAN release. (Fig. 32). Finally, the designed nanosystems were immobilized in a conventional layer of agarose matrix, giving rise to the so-called “sense-and-treat” hydrogel. The *in vitro* capability of this hydrogel to simultaneously detect and inhibit bacterial growth was tested in *E. coli* cultures in Petri dishes containing the hydrogel. The protons produced by bacteria not only produced a color change of the hydrogel from green to red, but also triggered VAN release that inhibited longer bacterial growth after 36 hours. Finally, as an example for monitoring and preserving the freshness of real samples, an *E. coli* infected tomato model was built and cultured on the surface of the designed hydrogel. Initially, the hydrogel exhibited green color under UV irradiation, whereas after 1 day in contact with the infected tomato, the hydrogel gave red luminescence under UV irradiation. After 4 days of incubation time, many colonies of bacteria appeared on the surface of the untreated control tomato. In contrast, tomato treated with the designed hydrogel was not affected; about 95% bacterial growth inhibition was estimated by counting of the colony forming units (CFUs).

The above-mentioned pH-gatekeeping mechanisms are mainly focused on using pH-cleavable linkages or polymers that can undergo pH-dependent conformational changes. However, other research groups have exploited the possibility of using pH-degradable blocking caps. For instance, Duan *et al.* described an innovative approach for the development of a robust β-lactam antibiotic (carbenicillin, CAR) and β-lactamase inhibitor (sulbactam, SUL), a pH-responsive co-delivery system able to disrupt the biofilm and attain better eradication of methicillin-resistant *S. aureus* (MRSA).^759^ To this aim, large-pore MSNs were loaded with SUL and then coated with a metal-CAR framework as pH-sensitive pore blocking units. CAR played a dual role, as an antibiotic to kill the bacteria and an organic ligand that coordinated with Fe^3+^ to form a pH-degradable metal organic framework (CARMOF) nanolid. Drug release experiments with the resulting MSN-Sul@CARMOF nanosystem at different pH values indicated a negligible SUL and CAR release at pH 7.4 and a fast release of both drugs at pH 5.0, which confirmed the acid-triggered dissolution of CARMOF. *In vitro* antimicrobial assays at different pH values indicated that the nanosystem inhibited MRSA growth at acidic pH thanks to the co-delivery of CAR (from the degradation of CARMOF) and SUL (loaded into the MSNs). Moreover, *in vitro* experiments in MRSA biofilms demonstrated enhanced penetration and efficient killing capability in the acidic environment inside the biofilm. Furthermore, no cytotoxicity was observed after incubation with RAW 264.7 cells for 48 h. Finally, *in vivo* assays in MRSA skin-infected mouse models demonstrated that the nanosystem effectively reverses MRSA infection.

In another report, Cheng *et al.* designed a new pH-responsive nanosystem, denoted MSN@FA@CaP@FA. The surface of ampicillin (AMP)-loaded MSNs was covered with double folic acid (FA) and acidic degradable calcium phosphate (CaP).^721^ Initially, AMP-loaded MSNs were decorated with FA *via* electrostatic interaction. Then the NPs were coated with CaP *via* a chelate effect and biomineralization. Finally, an additional FA layer was electrostatically immobilized onto the external surface of the nanosystem. The FA targeting ligand specifically guided the nanosystem to the bacterial infection site *in vitro*, increasing the uptake and diminishing the efflux pump effect in drug resistant *E. coli* and *S. aureus* bacteria. *In vitro* assays in mammalian cell cultures proved the biocompatibility of this nanosystem. Besides, the NPs exhibited no significant hemolytic activity in human blood at concentrations as high as 160 μg mL^−1^. *In vivo* studies in a drug-resistant bacterial infection mice model probed the excellent antibacterial effectiveness of this nanosystem, which decreased the mortality of drug resistant *E. coli* infection and prompted the wound healing process in drug-resistant *S. aureus* infection.

Abdelbar *et al.* used pH-degradable polylactic acid nanoflowers (PLA-NFs), whose structure was highly dependent on the environmental pH, for coating LEVO-loaded MSNs.^760^ Thus, at neutral pH PLA-NFs were insoluble, creating a compact capping layer on MSNs that prevented the premature LEVO release. In contrast, at acidic pH the nano-shell was hydrolyzed and degraded, allowing pore uncapping and LEVO release. The antibacterial efficiency of this nanosystem against *S. aureus* and *E. coli* bacterial strains was demonstrated. Furthermore, the nanosystems were cytocompatible and non-toxic, confirmed in *in vitro* studies on human osteoblast cells.

Another interesting strategy to treat bacterial infection relies on the combination of antiseptics, such as antibiotics and antimicrobial metal cations, with a unique MSN nanoplatform. Actually, this approach not only reduces the risk of antibacterial resistances but also produces synergistic bactericidal efficacy and multiple functions depending on the chosen cation, as reported for G3-targeted LEVO-loaded MSNs incorporating M^*n*+^ ions (M^*n*+^ = Ag^+^, Zn^2+^), as previously mentioned.^625^ It would also be desirable to provide this type of nanosystems with pH-responsive dual release capability. With this goal in mind, Lu *et al.* chose an antiseptic combination consisting of chlorhexidine (CHX), a broad-spectrum antibiotic, and Ag^+^ ions, whose joint administration yields synergistic bactericidal efficacy, to develop pH-responsive MSN co-delivery systems.^761^ Firstly, MSNs bearing amino groups were synthetized by a co-condensation route, and then reacted with the silver diammine cation [Ag(NH_3_)_2_]^+^ to obtain nanosilver-decorated MSNs. The further functionalization of the resulting NPs with carboxylate groups allowed the loading of the positively-charged CHX molecules by electrostatic interaction. Under acidic pH, protonation of carboxylate groups reduces electrostatic interactions, thus triggering CHX release. At the same time, protonation could break Ag–O bonds on the surface of MSNs, promoting Ag^+^ release. *In vitro* assays demonstrated that the nanosystem exhibited excellent bactericidal activity on G^+^*S. aureus* and G^−^*E. coli* bacteria at a low concentration, along with good biocompatibility on normal cells at the efficient antibacterial doses.

Another important challenge that can be tackled by pH-responsive MSNs is the treatment of intracellular infections, such as tuberculosis^762,763^ or tularemia,^438,764^ where macrophages are the primary bacteria-infected cells. As previously discussed, MSNs are usually well taken up by the cells from the mononuclear phagocyte system and, where this is not desired, such as in cancer therapy, special surface modifications are required to reduce their uptake by macrophages. Nonetheless, when aiming at treating intracellular infections, MSN uptake by macrophages and traffic to acidified endosomes is desired, since it brings up the possibility to intracellularly deliver antibiotics selectively inside the acidic endosomal/lysosomal compartments in cells. For example, Zink's research group designed pH-gated MSNs as delivery platforms of isoniazid (INH),^762^ one of the first-line drugs to treat tuberculosis. However, it is associated with serious toxic side effects, such as hepatotoxicity, which limit the doses that can be used clinically. To address this problem, MSNs were equipped with pH-dependent beta-cyclodextrin (β-CD) nanovalves, which open and release the payload in response to endosomal acidification, as already reported for anticancer purposes.^435^*In vitro* studies on human macrophages infected with *M. tuberculosis* proved that INH released by pH-gated nanosystems killed 1.5 logs more intracellular *M. tuberculosis* than an equivalent amount of free INH.^762^ Although efficacy was *in vitro* demonstrated, the low amount of INH loaded may be a handicap for application *in vivo*. Thus, the same group proposed an alternative strategy consisting of covalently linking INH to MSNs *via* a pH-cleavable hydrazone bond to form a prodrug nanoparticle-based system, which was well-tolerated *in vivo* and exhibited greater efficacy than could be reached with equivalent doses of free INH in a mouse model of pulmonary tuberculosis.^763^

However, moving back to the above-described approach involving β-CD as blocking caps in pH-responsive MSNs, it provided proof-of-concept for further development of MSNs comprising analogous molecular nanovalves for the delivery of the broad-spectrum fluoroquinolone moxifloxacin (MXF). The nanosystem was *in vivo* evaluated, showing not only lower toxicity but also much more efficacy than an equivalent amount of free MXF in the treatment of *F. tularensis* infection in a mouse model of pneumonic tularemia.^438^

##### Redox potential

**Fig. 33:**
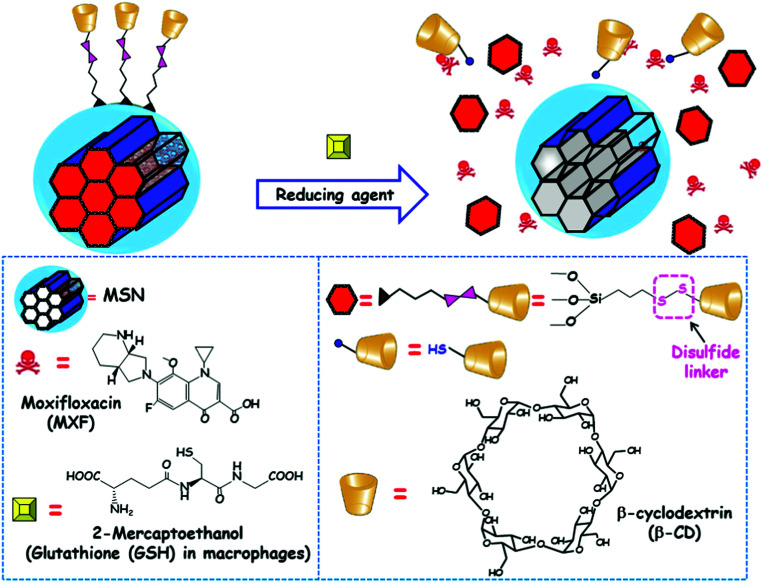
Schematic illustration of the functional mechanism of redox-responsive disulfide snap-top MSNs as a MXF release system for killing *F. tularensis* in infected macrophages. The exposure to reducing milieu (2-mercaptoethanol in the lab or GSH inside the macrophages) triggers the cleavage of the disulfide bond, leads to the removal of the β-CD blocking caps, and allows the release of the loaded MXF.^766^ Adapted with permission from ref. 766. Copyright 2021, MDPI.

As previously explained, living cells have more reducing power than the extracellular medium or plasma, owing to the high number of redox pairs that are kept primarily in the reduced state by diverse metabolic processes. Among the different redox couples, the glutathione (GSH)/GSSG couple is the most abundant inside the cells.^765^ This fact was exploited by Zink and co-workers, who developed redox-responsive disulfide snap-top MSNs as MXF release systems for killing *F. tularensis* in infected macrophages.^766^ The synthetic procedure involved the functionalization of MSNs with (3-mercaptopropyl)trimethoxisilane and further reaction with adamantanethiol, forming a disulfide bond. Then, MXF was loaded into the nanosystem, and β-CDs were added, which acted as the capping agents through the formation of an inclusion complex with adamantanethiol, as schematically illustrated in Fig. 33. The exposure to a reducing milieu, 2-mercaptoethanol in the laboratory or GSH inside cells, results in the cleavage of the disulfide bond, removing the bulky β-CD cap and releasing MXF from the mesopores. To evaluate the capability of these MSNs functionalized with disulfide snap-tops to release the cargo in response to the intracellular GSH levels, Hoechst fluorescent dye was loaded into the nanosystems. *In vitro* assays confirmed that these nanosystems released Hoechst exclusively intracellularly and stained the nuclei of macrophages. Moreover, MXF-loaded nanosystems were efficient at killing *F. tularensis* in macrophages in a dose-dependent manner. Furthermore, *in vivo* assays in a mouse model of lethal pneumonic tularemia indicated that these redox-responsive MXF MSNs significantly reduced the burden of *F. tularensis* in the lungs, liver and spleen, being more efficacious than a comparable amount of free antibiotic, meanwhile preventing weight loss, illness and death *in vivo*.

Very recently, Li *et al.* reported an alternative approach consisting of the design of GSH-degradable mesoporous organosilica NPs (MONs) for the co-delivery of gentamicin (GEN) and silver ions for the synergistic treatment of antibiotic-resistant bacteria.^767^ In this study, the authors prepared disulfide-bridged Ag-MONs using a one-pot sol–gel method that required the addition of tannic acid, which acted not only as a non-surfactant template for the formation of MONs but also as a reductant for the formation of nanoAg. The subsequent loading of GEN afforded the Ag-MONs@GEN nanoplatform. The GSH-responsive matrix degradation of this nanosystem allowed the simultaneous release of GEN and Ag^+^. This was explained by promotion of the dissolution of nanoAg by GEN, as reported elsewhere,^768^ which not only favoured the attachment of nanoAg to the bacterial surface but also aided the release of Ag^+^: this induced the generation of reactive oxygen species (ROS) that eventually kill bacteria. The synergistic antibacterial effect of the Ag-MONs@GEN nanosystem through inducing ROS generation was confirmed for four antibiotic-resistant bacteria, *i.e. E. coli*, *P. aeruginosa*, *S. aureus*, and *E. faecalis*. Moreover, the biocompatibility of the nanosystem was demonstrated in L929, HUVECs, and Raw 264.7 cells. This report opens new insights into the management of antibiotic-resistant bacteria through integration of GEN and nanoAg into GSH-biodegradable MSN-derived nanoplatforms. In this research line, Lu *et al.* developed dual redox/pH responsive MSNs for the delivery of CHX and Ag^+^ ions from biodegradable MSNs against oral pathogenic biofilms.^769^ In this case, disulphide-bridged MSNs were decorated with nanoAg and then functionalized with carboxylate groups for CHX loading *via* electrostatic interactions. Release experiments indicated that the nanosystem showed dual redox/pH-response of CHX and Ag^+^, which was ascribed to the GSH-triggered mesoporous matrix degradation, protonation of carboxylate functional groups, and accelerated dissociation of nanoAg in the acidic milieu. *In vitro* assays showed that the nanosystem exhibited improved capability to inhibit the growth of *S. mutans* and its biofilms. Importantly, the nanoformulation was more efficient than an equivalent amount of free CHX in limiting the formation of *S. mutans* biofilm by inducing bacterial cell death. Finally, the nanosystem prominently showed reduced toxicity as compared to free CHX in oral epithelial cells, and produced a non-anomalous effect in mice following oral exposure.

**Fig. 34:**
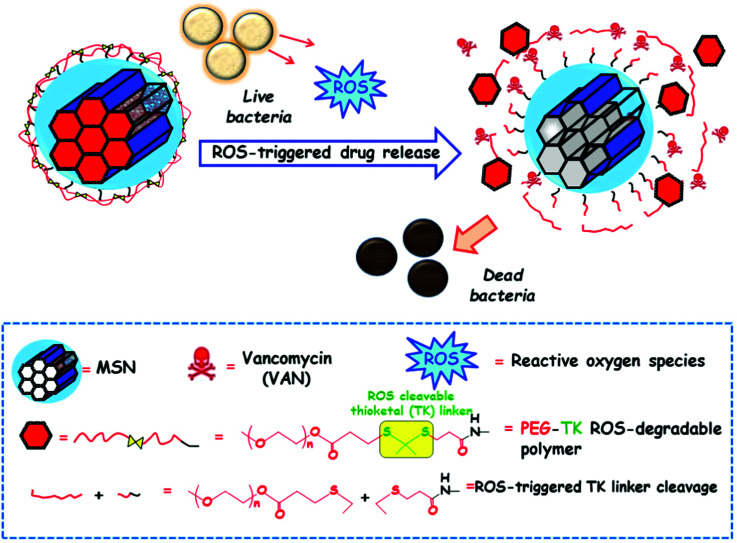
Schematic depiction of the mechanism of action of reactive oxygen species (ROS)-responsive MSN-based antimicrobial nanosystems. MSNs were loaded with vancomycin (VAN) and functionalized with a ROS-degradable thioketal grafted methoxy poly(ethylene glycol) (mPEG-TK) capping shell. The presence of ROS in the bacterial microenvironment produces the cleavage of TK linker and polymer degradation. This leads to pore uncapping, VAN release and eventually bacterial death.^771^ Adapted with permission from ref. 771. Copyright 2020. Elsevier Ltd.

Among the redox stimuli, the just-mentioned ROS is one type of redox stimuli that are present in living organisms usually. The stimuli principally include superoxide (O_2_^−^), hydrogen peroxide (H_2_O_2_), hypochlorite (OCl^−^), peroxynitrite (ONOO^−^) and the hydroxyl radical (–OH). ROS play an important role in pathogen resistance and cellular signaling.^714^ Actually, ROS are highly toxic to pathogens, acting as potent defensive tools to prevent tissue colonization by pathogenic microorganisms.^770^ Taking advantage of the ROS overproduction at the bacterial infection sites, ROS-responsive MSNs bring up new opportunities for the treatment of bacterial infection. Very recently, Li *et al.* developed a ROS-responsive nanosystem based on amino-functionalized MSNs loaded with VAN and linked to thioketal (TK) functionalized methoxy poly(ethyleneglycol) (mPEG-TK) as the ROS-responsive gatekeeper (Fig. 34).^771^ The interaction with the ROS-rich microenvironment results in the cleavage of the TK linker and degradation of the surface polymer, triggering pore uncapping and VAN release. *In vitro* evaluation of the antimicrobial efficiency demonstrated that, compared to VAN directly loaded into MSNs, the full nanosystem exhibited remarkably better controlled release and antibacterial activity against *S. aureus* due to the strong influence on the bacterial membrane's disintegration. Furthermore, good biocompatibility in osteoblast cell cultures *in vitro* and significant antibacterial efficiency in healing of skin wounds in rats exposed to *S. aureus* availed the potential of these nanosystems for topical applications, *i.e.* the treatment of infected wounds.

#### 4.3.4. External stimuli-responsive MSNs

External stimuli are gaining increasing interest, since they provide the clinician with higher spatiotemporal remote control over antimicrobial release. The addition of certain chemical species or the application of physical triggers (temperature, light or alternating magnetic fields, AMF) has been used to design and develop sophisticated smart MSN-based nanodevices.

##### Chemical species

An original research study by Li *et al.* reported the fabrication of a novel supramolecular nanoassembly for effective bacterial detection and adamantaneamine (AD)-responsive bacterial elimination.^772^ First, MSNs were loaded with the antibiotic AMO, and surface coated with 1,2-ethanediamine (EDA)-modified polyglycerol methacrylate (PGEDA). Then, cucurbit[7]uril (CB[7]) was anchored to the resulting NPs through the formation of inclusion complexes with EDA involving ion–dipole interactions. In the final step, negatively charged tetraphenylethylene carboxylate derivative (TPE-(COOH)_4_) was further linked to positively charged supramolecular polymers on the surface of MSNs *via* electrostatic interactions, affording a LbL supramolecular nanoassembly. When bacteria get in contact with this nanoassembly, the binding of the negatively-charged bacterial surface towards the cationic PGEDA layer of this nanosystem can decrease or disrupt the interactions between the PGEDA layer and the TPE-(COOH)_4_ layer, producing an attenuated TPE-(COOH)_4_ release, which is attributed to the weakening of aggregation-induced emission (AIE). Thus, a bacterial detection limit of 2.5 × 10^6^ CFU mL^−1^ was obtained for *E. coli.* In addition, moderate antibacterial activity was observed when the nanosystem was tested against both *E. coli* and *S. aureus*. However, the addition of AD produced a much higher antibacterial ability of the nanoassembly. This fact was attributed to a competitive replacement to form a more stable AD⊂CB[7] complex, which disrupted the nanoassembly, resulting in the delivery of PGEDA and TPE-(COOH)_4_ and allowing AMO release from the mesopores.

##### Temperature

**Fig. 35:**
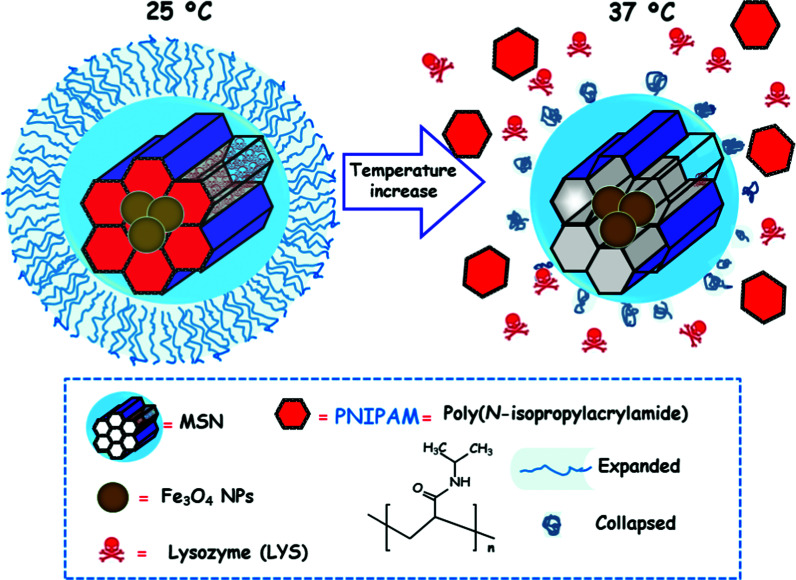
Schematic illustration of the temperature-responsive release behaviour of nanosystems consisting of Fe_3_O_4_ cores and large-pore mesoporous silica shells, which were loaded with lysozyme (LYS) and capped with the thermo-responsive polymer poly(*N*-isopropylacrylamide) (PNIPAM). At 25 °C PNIPAM is in extended conformation, closing the mesopores and impeding LYS release. When the temperature increases to 37 °C, PNIPAM adopts a globular conformation that produces pore opening and allows LYS release.^773^ Adapted with permission from ref. 773. Copyright 2015, Elsevier Ltd.

Temperature is an appealing physical trigger that has been the focus of applications in antimicrobial delivery from MSNs. For instance, Yu *et al.* designed and developed temperature-responsive core–shell protein delivery nanosystems. The nanosystems consist of iron oxide (Fe_3_O_4_) cores, and large-pore mesoporous silica shells, which were loaded with bacteriolytic enzyme LYS and capped with the thermoresponsive polymer poly(*N*-isopropylacrylamide) (PNIPAM).^773^*In vitro* experiments to evaluate the capping efficiency revealed that negligible LYS release occurred at 25 °C, since PNIPAM adopts an extended linear conformation at this temperature, blocking the pores and preventing the cargo release (Fig. 35). In contrast, upon increasing the temperature up to 37 °C, which is above the lower critical solution temperature (LCST) of PNIPAM (32 °C), the polymer undergoes a conformational change to a globular or collapsed form, uncapping the pore entrances and allowing LYS release. Antimicrobial assays of the nanosystem at different temperatures against G^+^*Bacillus cereus* and *Micrococcus luteus* revealed a marked temperature-dependent behavior at a nanosystem concentrations of 0.50 mg mL^−1^. Hence, bacterial growth showed a near 60% reduction for *B. cereus* and 45% for *M. luteus* of the initial CFUs count at 37 °C after 24 hours of assay, whereas no antimicrobial effect was detected at 25 °C.

##### Light

Among the different physical stimuli, light is receiving noticeable interest owing to the possibility to combine photo-triggered release of antimicrobials and phototherapy to fight bacterial infection. In a pioneering study, Kuthati *et al.* designed mesoporous silica trio-nanohybrids, consisting of Cu^2+^-impregnated MSNs loaded with curcumin (CUR) and superficially decorated with Ag NPs.^774^ Upon blue-LED light irradiation, the nanosystem exhibited outstanding photodynamic inactivation (PDI) capability against antibiotic-resistant *E. coli* due to the synergistic antibacterial effects of Ag, Cu^2+^ and CUR. Ag^+^ ion release controlled by light and reactive oxygen species (ROS) generation by both Ag and CUR provided the nanosystem with excellent antibacterial properties. Moreover, the positive surface charge provided by Cu^2+^ covering the MSN surface favored antimicrobial response *via* attractive electrostatic interactions with negatively charged bacterial cell membranes. Thus, the full nanosystem at 1.5 μM concentration produced *ca.* 90% bacterial cell eradication, which explained the improvement in bacterial killing around 5 log and 4 log in comparison to bacterial cells treated with free CUR and Ag NP-decorated Cu^2+^-impregnated MSNs, respectively, during visible light irradiation. Total eradication of bacterial cells occurred upon increasing the nanosystem concentration to 20 μg mL^−1^ (equivalent to 3 μM of pure CUR).

**Fig. 36:**
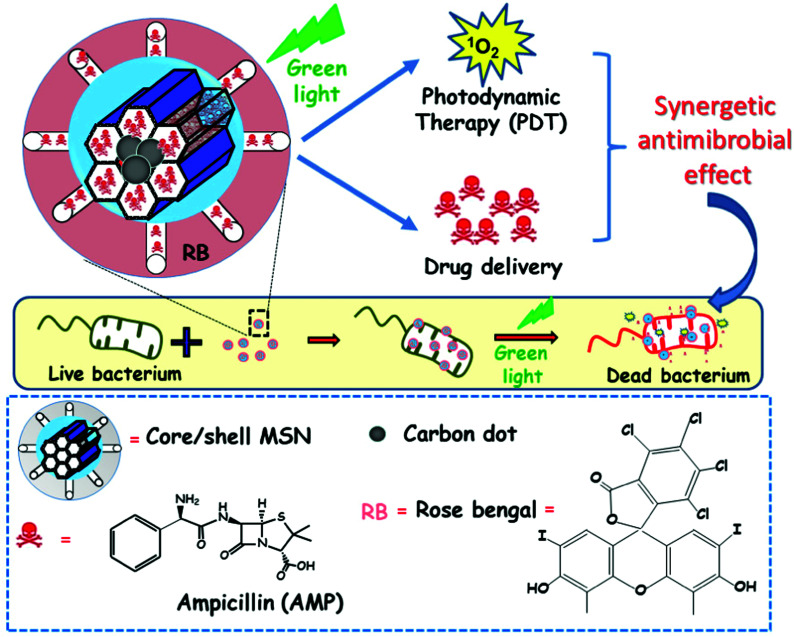
Illustration of the operative mechanism of light-responsive antimicrobial multifunctional nanoplatform consisting of core/shell structured MSNs embedding carbon dots (C-dots) and rose bengal (RB), respectively, and loaded with ampicillin (AMP). Under green light irradiation the RB photosensitizer facilitates the production of singlet oxygen species (^1^O_2_) to achieve photodynamic therapy (PDT), which in combination with AMP release led to synergetic antibacterial effect.^775^ Adapted with permission from ref. 775. Copyright 2017. Royal Society of Chemistry.

In another study, Liu *et al.* reported the development of a MSN-based multifunctional nanoplatform for imaging-guided antimicrobial/photodynamic synergetic therapy.^775^Fig. 36 schematically illustrates the composition of the nanosystem and its operating mechanism. The nanosystem, denoted as MSN@C-dots/RB/AMP, consisted of core/shell structured MSNs embedding carbon dots (C-dots) and a photosensitizer, rose Bengal (RB), respectively; an antibiotic, ampicillin (AMP), was loaded into the mesopores. C-dots serve as fluorescence probes to achieve cell fluorescence imaging; whereas RB allows generating singlet oxygen to accomplish effective photodynamic therapy (PDT); AMP loading aimed at enhancing the bacterial growth inhibition capability for antimicrobial purposes. *In vitro* assays against *E. coli* demonstrated that under green light irradiation, 100 μg mL^−1^ of the antibiotic-free nanosystem (MSN@C-dots/RB) produced significant reduction in the number of CFUs compared to the control (without light), which confirmed the generation of ^1^O_2_ under light exposure. Furthermore, complete *E. coli* growth inhibition was achieved after treating with 100 μg mL^−1^ of the MSN@C-dots/RB/AMP nanosystem under green light irradiation, which confirmed the boosted synergetic bacteria inhibiting effect.

Very recently, García *et al.* developed light-sensitive gold core@shell based MSNs with photothermal (PTT) and antimicrobial release capabilities.^776^ PTT has shown a bactericidal mechanism based on the efficient conversion of light, mainly in the 650–900 nm near infrared (NIR) spectral range, into local heating through the strong absorption of some metallic nanostructures.^777,778^ This nanosystem allows combining, locally at the biofilm, antimicrobial release and PTT effects. This does not only minimize the side effects associated with the systemic administration of antimicrobials,^747,749^ but also prevents healthy tissues from injuries accompanied by non-localized heating.^778^

**Fig. 37:**
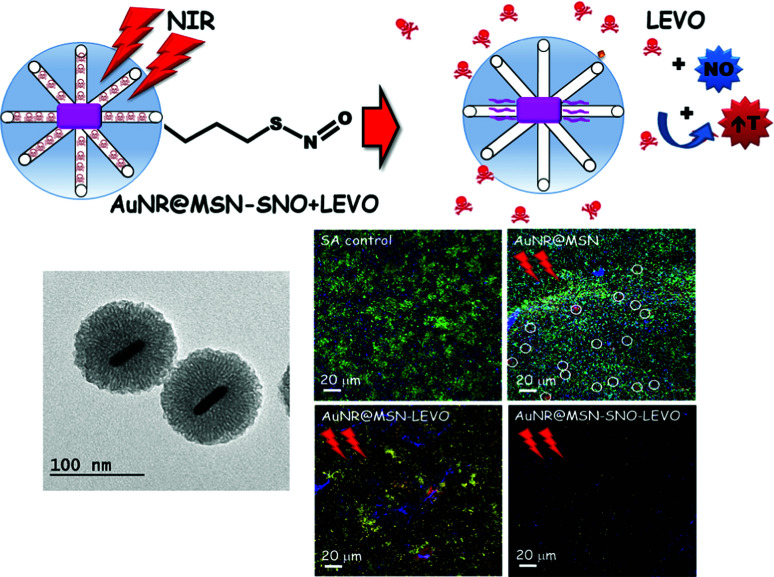
Top: schematic depiction of the design of the AuNR@MSN-SNO + LEVO nanosystem and the mechanism of action in response to near infrared (NIR) irradiation. Bottom left: transmission electron microscopy (TEM) images of AuNRs coated by a mesoporous silica shell and stabilized with a PEG (AuNR@MSN-PEGext nanosystem). Bottom right: Confocal microscopy images showing the antimicrobial action of the different nanosystems on G^+^*S. aureus* mature biofilms. The images show the preformed biofilm without any treatment (SA control), and after incubation with AuNR@MSN, AuNR@MSN + LEVO and AuNR@MSN-SNO + LEVO nanoassemblies followed by NIR irradiation. Live bacteria are stained in green, dead bacteria in red, and the protective polysaccharide matrix biofilm in blue. Ablation areas after incubation with the AuNR@MSN nanosystem are emphasized with white circles.^776^ Adapted with permission from ref. 776. Copyright 2021, Elsevier Ltd.

The synthetic procedure consisted of the nucleation of gold nanorods (AuNR) as cores for the subsequent growth of silica shells, resulting in the formation of an AuNR@MSN nanosystem exhibiting PTT properties.^776^ The incorporation of nitrosothiol groups (-SNO) with a heat responsive linker allowed an enhanced nitric oxide (NO) release by the temperature increase induced by NIR irradiation. The final step consisted of loading of the LEVO antibiotic into the mesoporous channels (Fig. 37). The release of exogenous NO from such a composite has demonstrated potential therapeutic application in bacterial infection treatments, being a key regulator of biofilm dispersal and an antibacterial action trigger through generating by-products that influence oxidative and nitrosative stress to kill the bacteria by multiple pathways.^779^ Moreover, its combination with conventional antibiotics was revealed to be a powerful anti-biofilm strategy, since biofilm dispersion aided by NO makes bacteria more susceptible to the antibiotic action.^780^ Therefore, the clever design of this multicomponent nanoassembly was considered as a potent nanotherapeutic able to affect the architecture of the *S. aureus* bacterial biofilm and later on inhibit its growth.^776^ The assays consisted of incubating mature *S. aureus* biofilms with different suspensions of the nanosystems at a concentration of 50 μg mL^−1^ at 37 °C for 90 min before the first NIR laser irradiation (808 nm, 1 W cm^−2^, 10 min). This process was repeated after another 90 min of incubation, and then all samples were incubated for further 24 h at 37 °C. The results indicated that the full nanosystem AuNR@MSN-SNO + LEVO provoked only a 31.4% reduction of CFU mL^−1^ in the biofilm in the absence of NIR laser irradiation, due to the sole effect of LEVO release. However, upon irradiation with the NIR laser a biofilm reduction of 88% was observed, which clearly proved that the local combination of antimicrobial (LEVO and NO) release and PTT improved the therapeutic efficacy of the nanosystem (Fig. 37).

##### Alternating magnetic field

**Fig. 38:**
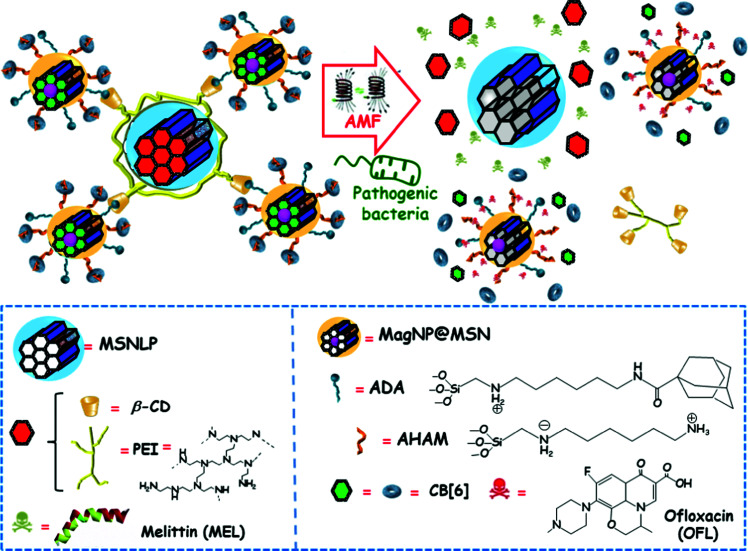
Schematic depiction of the mechanism of action of dual drug co-delivery and pathogen/alternating magnetic field (AMF)-responsive antimicrobial nanosystems constituted by the supramolecular co-assembly of heterogeneous MSNs. Host MSNs (H) are large-pore MSNs (MSNLP) loaded with melittin (MEL) and capped by β-cyclodextrin (β-CD)-modified polyethylenimine (PEI). Guest MSNs (G) consisted of superparamagnetic nanoparticles coated with a mesoporous silica layer (MagNP@MSN), loaded with ofloxacin (OFL) and grafted to both adamantine (ADA) (to interact with β-CD on the surface of H) and *N*-(6-*N*-aminohexyl)aminomethyl triethoxysilane (AHAM) (to interact with cucurbit[6]uril (CB[6])) for efficient pore capping. Dual antimicrobial drug release is triggered by the presence of pathogenic cells and the application of an alternating magnetic field (AMF).^781^ Adapted with permission from ref. 781. Copyright 2020, The American Chemical Society.

Magnetic fields exhibit the best penetration of tissue among the different external stimuli covered in this section. Under an AMF, superparamagnetic iron oxide NPs (SPIONs) produce heat. Therefore, MSNs loaded with antimicrobials, functionalized with thermosensitive blocking caps, and incorporating SPIONs are promising alternatives to design AMF-responsive nanosystems. In a pioneering study, Yu *et al.* designed an AMF-activated supramolecular nanoplatform for the co-delivery of antimicrobial agents for the synergetic eradication of pathogenic biofilms.^781^ This nanoplatform relied on the supramolecular co-assembly of heterogeneous MSN-based nanosystems (Fig. 38). Large pore MSNs (MSNLP) capped by β-CD polyethylenimine (PEI) became the host nanosystem (H, MSNLP@PEICD). A superparamagnetic nanoparticle core (MagNP) coated by a mesoporous silica layer, which was dually decorated with adamantine (ADA) (able to interact with β-CD on the surface of H) and *N*-(6-*N*-aminohexyl)aminomethyl triethoxysilane (AHAM) (able to interact with cucurbit[6]uril (CB[6])) for the effective mesopore capping, constituted the guest nanosystem (G, MagNP@MSNA-CB[6]). The host–guest interactions between β-CD and ADA result in spontaneous co-assembly of H and G, giving rise to a supramolecular cluster (H + G). The resulting nanoplatform was ideal to co-deliver antimicrobial agents with different molecular weights and charges. Thus, the large size and positively charged antimicrobial peptide melittin (MEL) was loaded into the pores of H, whereas the small-molecular weight and negatively charged antibiotic ofloxacin (OFL) was loaded in G. Under AMF application, the localized heating from SPIONs activated pore uncapping and OFL release from the G nanosystem. Moreover, the dual co-assembly (H-MEL + G-OFL) exhibited the positively charged PEICD cap, which provided the nanoplatform of bacterium-binding and -responsive antimicrobial delivery capabilities. This synergistic antimicrobial capability of the dual stimuli co-delivery responsive nanoassembly was *in vitro* evaluated against *P. aeruginosa* bacteria, showing a 97% biofilm mass removal and 100% pathogen cell killing. Finally, the dual drug-loaded co-assemblies demonstrated a strong capability to exhaustively eradicate *in vivo* pathogenic biofilms from implants, and they were very efficient at preventing host tissue infection and inflammation.

**Fig. 39:**
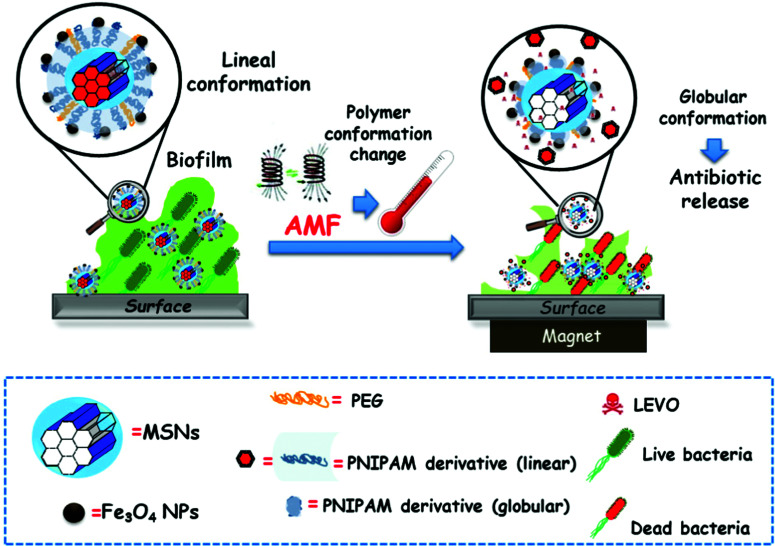
Schematic depiction of the operating mechanism of a superparamagnetic iron oxide nanoparticle (SPIONS) decorated mesoporous silica nanosystem for combined antibiofilm therapy.^782^ Adapted with permission from ref. 782, Copyright 2022, MDPI.

Very recently, Álvarez *et al.* reported the design of a new MSN-based magnetic nanosystem to combine the AMF-triggered release of antibiotics and magnetic hyperthermia against bacterial biofilms (Fig. 39).^782^ To this aim, MSNs were decorated with polyethyleneglycol (PEG), to improve colloidal stability, and with a thermosensitive poly-*N*-isopropylacrylamide (PNIPAM)-based polymer, which was able to experience a hydrophilic-to-hydrophobic (linear-to-globular) conformational change at a temperature between 40 and 43 °C. The external surface of the polymer-coated nanosystem was decorated with magnetite (Fe_3_O_4_) SPIONs, which acted as hot spots, provoking the contraction of the polymeric chains under the application of an AMF, as previously reported. When the nanosystem was loaded with a drug, LEVO in this case, this effect produced the release of the therapeutic cargo, as previously reported.^783^ The ingenious design of this nanosystem, where SPIONs decorate the outer surface, may allow the close contact of the magnetic NPs with the target biofilm, enhancing the bactericidal action since the heating is localized on the biofilm region and is not shielded by the silica structure. Besides, the slight antimicrobial effect of the SPIONs by themselves^784^ may be promoted by the particular design of the nanosystem. The microbiological assays revealed that the exposure of *E. coli* biofilms to 200 μg mL^−1^ of the nanosystem and the application of an AMF (202 kHz, 30 mT) reduced the number of viable bacteria by 4 log_10_ units compared with the control. This proof-of-concept, based on the combination of magnetic-hyperthermia therapy and thermo-responsive antibiotic delivery through the smart assembly of different functional building blocks into a unique MSN, constitutes a promising strategy for the efficient treatment of biofilm-associated infections.

### 4.4. Other therapies

#### 4.4.1. Multidrug approaches

As it has been mentioned above, chemotherapy is conventionally employed for the treatment of tumours with a tendency to metastasise. The use of nanocarriers to deliver appropriate drugs to the precise site of the disease can overcome some of the typical pitfalls of conventional chemotherapy, such as adverse side effects, low drug solubility and stability in physiological media, poor delivery kinetics, and multidrug resistance. Throughout this section, the use of MSNs has been demonstrated as a potential alternative to overcome these barriers. One of the advantages of using MSNs *versus* other types of nanocarriers is the ease by which two or more drugs can be incorporated into a single MSN. This was demonstrated for the case of delivering doxorubicin and cisplatin using hollow MSNs, where the anticancer activity was enhanced thanks to the simultaneous presence of both drugs.^785^ A similar approach was developed for the treatment of acute promyelocytic leukemia delivering paclitaxel and tanshinone IIA drugs,^786^ and to overcome the multidrug resistance of certain cancer stem cells by loading doxorubicin and tariquidar into MSMs.^787^

#### 4.4.2. Cancer immunotherapy

In the last few years, cancer immunotherapy has revolutionised the treatment of certain cancers aiming to improve anti-tumour immune responses with very few off-target effects. However, despite the great advances in this area, the clinical use of those immunotherapy approaches still presents several concerns regarding both efficacy and safety. Nevertheless, novel approaches for administering immunotherapy in a safer and more controlled manner is highly desirable. In this sense, the use of nanocarriers might improve the accumulation of immunotherapeutic agents within the site of the disease, allowing a better targeting and reducing the off-target adverse effects.^788^ In this sense, all cases of cancer immunotherapy, such as checkpoint inhibitors, cytokines, engineered T cells, co-stimulatory receptor agonists and cancer vaccines, can benefit from the employment of nanocarriers. Among all the possible NPs that can be employed for cancer immunotherapy, MSNs are promising candidates to improve this type of immunotherapy, because of their attractive properties to be used for delivery systems (high porosity, high biocompatibility, facile surface modification, and self-adjuvanticity).^789^ As has been repeatedly stated above, the interaction of MSNs with physiological environments can be tuned through their physical and chemical properties, including particle shape, size, porosity, and surface functionality. These properties have been observed to influence their interaction with immune cells. In fact, in a recent review of MSNs for cancer immunotherapy, the authors classify MSNs applied in immunotherapy into two groups based on particle size and how they interact with immune cells.^789^ In that excellent review, it is highlighted how the control of size, pore structure and potential functionalization of MSNs are critical factors in the improved immunogenic activity of antigen-presenting cells to induce an adequate immune response. Additionally, their capacity for delivering high amounts of different bioactive agents, such as drugs, proteins, peptides, genes, or even antibodies, can help to use the power of the body's own immune system to prevent, control, and eliminate cancer. In this regard, MSNs can transport immunomodulating agents, such as antigenic payloads and immune stimulators, in the absence of an adjuvant.

#### 4.4.3. Vaccines

Conventional vaccine technology has used a variety of adjuvants to improve immunogenicity through the activation of the dendritic cells and the generation of strong antigen specific immune responses. These adjuvants can be classified depending on their immunostimulatory effect over the antigen presenting cells and their capability as delivery systems to start the antigen uptake. In fact, finding an optimal adjuvant and antigen carrier combination is one of the most challenging milestones in vaccine development. Several nanocarriers have been evaluated to fulfil these requirements, such as polymeric or lipid NPs, although most of them are unstable and prone to degradation in the harsh gastric environment, leading to premature release of their cargo. The stability of mesoporous silica in the intestinal transit environment has fuelled its use as a vaccine adjuvant and/or antigen delivery system.^790–792^ Thus, MSNs have been investigated for delivering different types of antigens, such as goat IgG,^793^ vital antigen E2 from bovine viral diarrhoea virus^792^ or type 2 ORF protein from porcine circovirus,^698^ among others. Similarly, MSNs have been investigated as potential adjuvants in vaccine formulations with very promising results.^792,794,795^

#### 4.4.4. CRISPR delivery

Clustered regularly interspaced short palindromic repeats (CRISPR) is an adaptive immune system based on DNA sequences from bacteria that has been repurposed to be used in mammalian systems to edit the genome.^796^ Different viral vectors have been investigated as CRISPR delivery and gene editing systems with relative success, although those viral vectors might present some pitfalls in the clinical translation.^797^ Also other nanoplatforms have been investigated for CRISPR delivery with arguable success due to lack of stability and/or low loading capabilities^798–804^ Among them, MSNs have been investigated to transport CRISPR into different cancer cells, and the editing effects were demonstrated through a fluorescent reporter system.^805^ Since then, many different CRISPR systems have been loaded and released from MSNs with very promising results for future clinical applications.^340,806–810^

One of the reasons for the popularity of NPs in the area of clinical research is their ability to deliver drugs to diseased tissues in a controlled and selective manner, as discussed above, together with their ability to simultaneously provide information on the status of disease progression. These nanosystems capable of performing this dual function, drug delivery and diagnostics, are known as theranostic nanocarriers. Thus, it is possible to monitor the disease condition and simultaneously treat it with a single moiety known as theranostic nanomedicines.^811^ This is possible thanks to the incorporation of different types of imaging agents into the nanocarriers, such as fluorophores,^812^ magnetic^813^ or ultrasound^814^ contrast agents or radionucleotides.^815^ Among the different nanocarriers for theranostic purposes, MSNs have been widely employed, due to their physicochemical characteristics. For instance, contrast agents can be located in the silica network, and the therapeutic payload can be loaded in the pores of the particles. Excellent reviews on this type of nanocarriers have been published in the last few years,^816–818^ where more detailed information on the investigated MSN delivery and imaging systems can be found.

#### 4.4.5. Neurodegenerative diseases and the blood–brain barrier

The blood brain barrier (BBB) preserves the internal environment of the brain and its homeostasis, limiting the penetration of therapeutic drugs that are targeted to the central nervous system (CNS).^819^ BBB consists of tightly packed cerebral capillary endothelial cells and pericytes and is located between circulating blood and neural tissues. In diseases such as Parkinson's disease (PD), Alzheimer's disease (AD) or stroke, systemic administration of almost 100% of molecules larger than 500 Da and 98% of the remaining small molecules fail to cross the BBB, thus preventing their effect on the CNS.^820–822^ During intracerebral hemorrhage, infections or in neurodegenerative diseases, the BBB is altered, facilitating access to inflammatory molecules.^823^

In this sense, different approaches to drug delivery routes are needed to treat these diseases, avoiding the BBB. These alternatives include blood-to-brain and intranasal administration coupled with biological, chemical or physical stimuli, and intracerebral routes.^822^ However, all these alternatives have clear disadvantages such as high cost, hazardous nature and not adapting to neurological diseases without disrupting or damaging the BBB.^820,821^

Therefore, nanobiotechnology is an interesting and promising alternative to transport drugs in a controlled way across the BBB without damaging it. In addition to being functionalised and targeted specifically to the site of damage, these nanosystems are internalised by endothelial cells in brain capillaries through endocytosis and transcytosis.^820,821^ Liposomes and inorganic and polymeric nanoparticles are the most studied nanosystems in this field. In the case of inorganic nanoparticles, the most prominent are MSNs and carbon nanotubes.^822,824^ Inorganic NPs are more versatile and tractable by imaging techniques, while natural NPs bind more specifically to biological receptors located on endothelial cells.^821^

In this context, MSNs widely used in biomedical applications are under study as therapy in degenerative and inflammatory diseases of the CNS with respect to their effects on the BBB.^822,824^ The majority of studies implicated on the use of MSNs and their BBB pathology are focused on diseases such as AD,^825–827^ PD,^820,821^ glioblastoma,^820,828^ intracerebral hemorrhage^820^ and nerve agent detoxification.^829^

As we previously mentioned, transcytosis is a selective and non-invasive delivery mechanism through NPs, which can cross the BBB. Using this concept, different nanosystems based on MSNs have been developed that use this pathway to deliver therapeutic drugs without disrupting or altering the BBB.^820,821^ In this sense, in a study by Baghirov *et al.*,^830^ MSN functionalized with PEG–PEI was successfully internalized by RBE4 rat brain endothelial cells and Madin–Darby canine kidney epithelial cells, as *in vitro* models of the BBB. In the brain vasculature *in vivo*, these nanosystems were visualized after systemic injection and did not induce impairment to the BBB, indicating their good properties as a treatment vehicle.

Studies on the most effective MSNs targeting brain microvessel endothelial cells have been performed with transferrin, lactoferrin and insulin receptors.^820,821^ In this regard, PEG-MSNs with two different sizes (50 and 160 nm) were synthetized and conjugated with rat Ri7 transferrin receptor antibodies by Song *et al*.,^831^ showing high binding affinity and specificity *in vitro.* On the one hand, Ri7-MSNs were efficiently internalised (endocytosis) by brain neuronal and endothelial cells in a size- and time-dependent manner. The highest internalisation was observed for the 50 nm particles and, after systemic administration, the particles accumulated specifically in the endothelial cells of brain microvessels *in vivo.* On the other hand, of particular interest is a study by Huang *et al*, where in an *in vitro* co-culture system mimicking BBB, PEG-MSNs grafted with lactoferrin (Lf) were able to cross the BBB without affecting cell viability.^832^ The smaller the particle size, the more successful the transcytosis with maximum effectiveness detected for 25 nm. Lf is a cationic glycoprotein highly expressed in cerebral capillary endothelial cells and an excellent targeting agent due to low cost, exceptional biocompatibility and receptor-mediated transport efficacy.^832,833^ Based on these results it seems that Lf ligands could be the optimal candidate for targeting the BBB. In addition, Tamba *et al*.,^834^ using a microemulsion method, designed a MSN nanosystem functionalized with glucose (Glu) and glucose-poly(ethylene glycol) methyl ether amine (Glu-PEG) to penetrate the BBB in rodent brains. The nanosystems were efficiently internalized by neuronal cells due to the combination of PEG-amino groups and glucose. Biodistribution and penetration studies showed that Glu-PEG-MSNs administered systemically crossed the BBB through vascular endothelial cells *via* receptor-mediated endocytosis and transcytosis through several transporters such as glucose transporter and others.^834^

Although the present results are encouraging in terms of BBB targeting and efficiency in the treatment of a variety of brain alterations, MSN based nanosystems remain to be evaluated in further *in vivo* models that mimic different neurological diseases and to demonstrate their efficient penetrability as well as non-damaging nature to the BBB.

## 5. Perspectives for clinical translation

Any treatment applied to the clinic, especially for the treatment of cancer, is based on the search for personalised medicine capable of adapting to the specific tumour to overcome the many limitations of traditional treatments which, although they succeed in mitigating or curing the disease, in many cases fail.  Most of the drugs developed to the treatment of cancer have been evaluated using stratified studies. These type of studies should be applied to the test of NPs, which are usually evaluated in non-stratified studies. This seems an important point to overcome if NPs are to see accelerated evolution in clinical trials, incorporating specific stratified patient populations leading to a more homogeneous treatment response.

In this sense, NPs are ideal candidates for personalized therapies as they can neutralize factors such as heterogeneous biological barriers and comorbidities. Different approaches with nanoparticles have been used for the diagnostic detection of different diseases.^835^ In addition, they have been used within the tumour microenvironment to promote the accumulation and penetration of the particles and thus increase the efficacy of drugs.^836,837^

Different types of nanoparticles have been approved by the FDA for clinical applications, with more than 30 approved nanodevices and more than 100 in clinical trials.^247^ These include lipid nanoparticles such as Doxil for the treatment of ovarian cancer or for the cure of leukemia (Marqibo or Viseox, among others), polymer-based nanoparticles (Oncaspar and Copaxone, among others) or inorganic nanoparticles for the treatment of anemia (INFeD or DexFerrum) or kidney diseases (Venofer or Ferahem).^241^ In addition, with the emergence of COVID and lipid nanoparticle-based mRNA vaccines, these NPs have been shown to be safe treatments and have demonstrated their great potential for the treatment of different diseases.

Concerning MSNs, their advantages for further clinical studies over organic or other inorganic NPs have already been extensively discussed in this review. In this regard, the food additive E551 is composed of 100 nm MSNs and colloidal silica has been used in the manufacture of tablets as a glidant, both of which are FDA approved.^247^ As a result, several formulations based on silica nanoparticles are now in phase I and II clinical trials. The safety, efficacy and viability of MSNs in humans are evident from 11 clinical trials and 2 clinical studies, including oral drug delivery and diagnostics.^247^ Despite preliminary data pointing to high safety and efficacy in their treatment in clinical trials, the translation of these nanoparticles to the clinic is very slow. One of the possible reasons is that most of the preclinical studies have been focused on small animal models such as rodents, whereas a few studies have been performed in large model animals such as pigs, sheep or monkeys, much more human-like animals.

In our opinion it seems that the key to making the final leap to the clinic lies in demonstrating the long-term safety of MSNs, testing different routes of administration, being able to scale up their production and thus making the synthesis reproducible on a commercial scale. In addition, optimization of the design of MSN-based nanosystems using FDA-approved elements whenever possible could be desired to allow easier translation to clinical trials.

## 6. Conclusions

Although silica is usually employed as an excipient in many drug formulations, silica-based nanocarriers have not been accepted yet by the regulatory agencies of different countries to be used in the clinic. However, there are other types of silica NPs that are currently undergoing clinical trials, such as C-dots (7 nm silica NPs for imaging in metastatic melanoma). Nevertheless, MSNs for drug delivery are still in the preclinical stage. They have been evaluated for oral administration in humans, finding that they are well tolerated and safe.^838^ They have also been evaluated as food additives with very promising results for a potential clinical trial.

For formulations relying on MSNs as carriers to be translated to clinical use, approval of MSNs by the regulatory bodies as a carrier material for different modes of administration is of highest importance. This does not necessarily include other components of complex composites, such as luminescent reporter species, capping agents, or others, which impart the functions highlighted throughout the text. However, if the basic ingredient, the MSN, is not approved, all other studies are lacking a sound foundation.

The world has observed the super-fast development of COVID-19 vaccines, which has been undoubtedly a spectacular success of science in this area. However, the quick advances in the formulation of the mRNA vaccines was possible thanks to vast previous research activities and experience in the basic science on nanovehicles, including lipid NPs. Similarly, these last 20 years of basic research on MSNs are expected to pave the way for future clinical applications using this type of nanocarriers, which are expected to come sooner or later. It is now the time to take steps in resolving regulatory issues with one or the other promising formulation for a disease with a high medical need and high potential advantages on MSN-based drug formulations. Herein, the discussion of the different approaches clearly demonstrates that even multifarious systems can be constructed with high precision, and it is expected that, should the need arise, they can be produced at scale and in amounts to treat high number of patients suffering from complex diseases.

The present review has tried to collate in a comprehensive and systematic manner the development history and current achievements on MSN based drug delivery systems, covering different synthetic routes, property engineering and disease treatment. The significance of this review relies on the fact that the story has been told by those who were among the pioneers in mesoporous materials and their applications in drug delivery technologies. This angle provides a particular view on the early obstacles encountered, the thought processes in choosing specific approaches, and a deeply experienced-based perspective on potential future developments. Fresh minds, who use this overview as a starting point will for sure pursue new avenues, and we look forward to see the new directions this field will take in the future.

## Author contributions

All authors have contributed equally to this manuscript.

## Conflicts of interest

There are no conflicts to declare.

## Supplementary Material
